# Cav3.1 Is a Neuronal Leucine Sensor That Mediates Satiety and Weight Loss in Response to Dietary Protein

**DOI:** 10.1016/j.cmet.2026.03.017

**Published:** 2026-04-22

**Authors:** Anthony H. Tsang, Nicholas Heeley, Constanza Alcaino, Eunsang Hwang, Brian Y. Lam, Taufiq Rahman, Tamana Darwish, Danae Nuzzaci, Richard G. Kay, Amar Sarkar, Ruiyan Wang, Nihal Basha, Austin Punnoose, Peter Kirwan, Marcella Ma, Giles S. Yeo, Florian T Merkle, Fiona M. Gribble, Frank Reinmann, Kevin W Williams, Clémence Blouet

**Affiliations:** 1 Institute of Metabolic Science-Metabolic Research Laboratories, University of Cambridge, Cambridge, UK; 2 Department of Internal Medicine, Center for Hypothalamic Research, University of Texas Southwestern Medical Center at Dallas, Dallas, Texas, USA; 3 Department of Pharmacology, University of Cambridge, Cambridge, UK

**Keywords:** Hypothalamus, Arcuate Nucleus, POMC neurons, Voltage-Gated Calcium Channel, Nutrient Sensing, Appetite, leucine, dietary proteins, Metabolic diseases, Obesity

## Abstract

Dietary protein promotes satiety and weight loss but an understanding of appetite-regulating neurons sense dietary protein intake is lacking. Here we show that *Cacna1g*, encoding for the T-type voltage-gated calcium channel Cav3.1, is enriched in hypothalamic leucine-sensing neurons and mediates neuronal leucine sensing. Pharmacological inhibition of Cav3.1 blunts leucine-induced activation of POMC neurons in cultured neurons and brain slices, as well as the anorectic response to hypothalamic leucine sensing in vivo. Genetic deletion of *Cacna1g* in POMC neurons abolishes the appetite- and weight-suppressive effects of high protein feeding. Mechanistically, we show that leucine binds to a hydrophobic pocket located between the voltage-sensing S4 segments of Domain II and III of Cav3.1, and thereby reduces its threshold for voltage-dependent activation. Last, we show that pharmacological activation of mediobasal hypothalamic Cav3.1 promotes weight loss in diet-induced obese mice and potentiates the weight loss response to anorectic agents such as liraglutide. Together, these results highlight the role of hypothalamic Cav3.1 as a neuronal leucine sensor and its relevance as a target for anti-obesity treatment.

## Introduction

A rapid and efficient response to disturbances in nutrient levels is crucial for the survival of organisms from bacteria to humans. Cells have evolved a host of molecular pathways that can sense nutrient concentrations and quickly regulate their activity and gene expression to adapt to changes in nutrient availability. In complex organisms, distributed populations of specialised nutrient-sensing cells continuously monitor nutrient availability and orchestrate a range of systemic metabolic responses to optimise inter-organ nutrient fluxes, allowing the maintenance of nutritional homeostasis. These include enteroendocrine cells in the gut, or endocrine cells of the pancreas. In the brain, monitoring of nutrient availability by specialised nutrient-sensing neurons provides an additional level of integrated regulation through the autonomic nervous system, and directly engages behavioural components recruited for nutritional homeostasis, i.e. hunger and satiety or macronutrient preference.

While the responses to shortage or excess in carbohydrate and fat are facilitated by the presence of storage compartments for these macronutrients in the liver and adipose tissue respectively, deviations in protein availability are optimally addressed through behavioural strategies in the absence of storage compartment for protein. However, how the brain senses protein availability to regulate appetite is poorly understood. It is well established that dietary proteins trigger the secretion of appetite-suppressing incretins during the digestion process ^1^, but postprandial excursions in circulating levels of specific amino acids also reach the brain to directly activate appetite-suppressing neurocircuits ^2,3^. These include the branched-chain amino acid Leucine, recognised as a signalling molecule reporting amino acid availability to regulate a variety of anabolic processes and produce satiety. However, the molecular identity of leucine-sensing neurons is poorly characterised. Leucine-sensing neurons represent a molecularly diverse population of neurons which includes pro-opiomelanocortin (POMC) neurons of the arcuate nucleus of the hypothalamus (ARH) ^4–6^. Leucine-induced mTOR signalling mediates leucine’s anorectic action ^4,7,8^, but POMC neurons rapidly respond to leucine independently of mTOR signalling through a mechanism that remains unclear ^6^.

Here we used an interdisciplinary approach to establish the molecular identity of hypothalamic leucine-sensing neurons, the mechanisms of leucine-induced neuronal activation, and their relevance in the appetite- and weight-suppressive action of high protein feeding.

## Results

### Molecular profiling of hypothalamic leucine-sensing neurons identifies *Cacnag1* as a top marker

**Figure 1:**
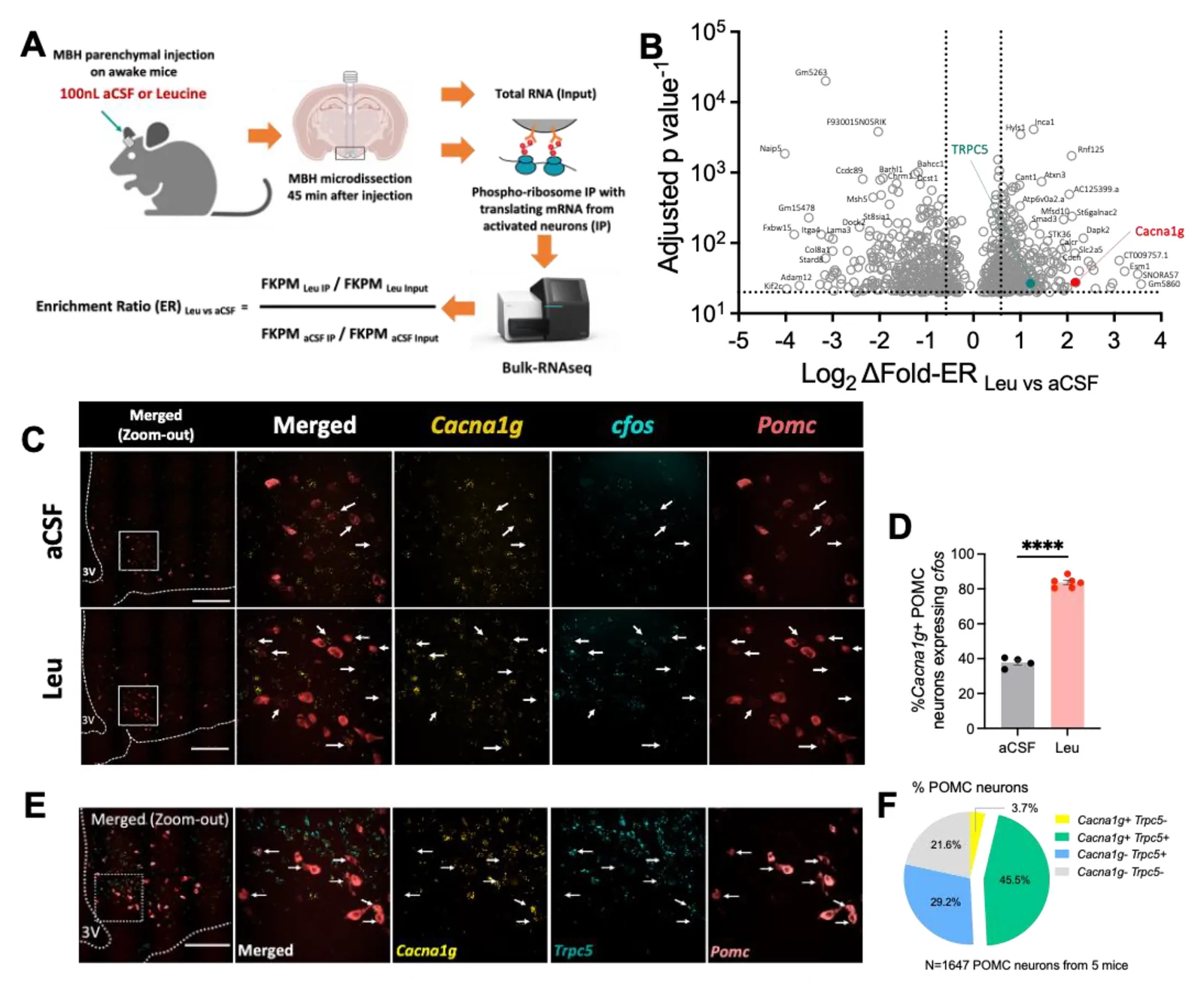
Molecular profiling of hypothalamic leucine-sensing neurons identifies *Cacnag1* as a top marker **(A)** Experimental design for PhosphoTRAP assay. **(B)** Transcripts differential enrichment in MBH leucine-responsive neurons. **(C-D)** Representative image **(C)** and quantification **(D)** of multiplexed FISH against *Cacna1g, cfos* and *Pomc* following MBH leucine administration. White arrows highlight *Cacna1g*+/*cfos*+ POMC neurons. Scale bar: 200 μm, n = 4-6. Values are mean ± SEM. ****p<0.0001, unpaired t-test. **(E-F)** Representative image **(E)** and quantification **(F)** of multiplexed FISH against *Cacna1g, Trpc5* and *Pomc*. White arrows: *Cacna1g*+/*Trpc5*+ POMC neurons. Scale bar: 200 μm. **(F)** Relative proportion of MBH POMC neurons expressing *Cacna1g* and *Trpc5*, n = 1647 POMC neurons from 5 WT mice. **See also** Figure S1.

We used the PhosphoTRAP assay following parenchymal injection of leucine into the MBH as previously described (Figure 1A)^5^. Neurons activated following MBH leucine injection (and expressing the neuronal activation marker cFos) co-express Ser240/244-phosphorylated ribosomal S6 protein (p-rpS6, Figure S1A), allowing selective immunoprecipitation-based capture and sequencing of polysomes from leucine-activated neurons. RNA sequencing of total MBH extracts (input sample) confirmed enrichment in ARH-specific genes, validating the neuroanatomical accuracy of the dissections (Figure S1B). Further, activity-dependent transcripts were significantly enriched in p-rpS6 immunoprecipitated samples (IP sample; Figure S1C), confirming enrichment in translating transcripts from activated neurons. Differential expression analysis between IP and input samples generated a list of differentially enriched transcripts in leucine-activated neurons (Figure 1B, Table S1-S4). We validated these results using RT-qPCR on a panel of 41 genes spanning over the entire enrichment ratio spectrum, revealing a good correlation with the RNAseq analysis (r^2^=0.405, p<0.0001) (Figure S1D). *Calcr* was among the top enriched genes, in line with our previous work implicating CALCR neurons in brain leucine sensing^9^. In addition, IPA pathway analysis revealed activation of p70/S6K and ERK/MAPK signalling pathways, in line with our previous observations implicating these signalling cascades in MBH leucine sensing (Figure S1E, Table S5) ^10^.

*Cacna1g*, encoding the T-type Ca^2+^ channel Cav3.1, was among the top enriched transcripts encoding plasma membrane proteins (Table S3). Based on our previous work identifying extracellular Ca^2+^ entry as an early event required for leucine sensing in POMC neurons ^6^, and work from others indicating that leucine-induced Ca^2+^ currents contribute to mTORC1 activation ^11^, we chose to further validate this marker. We used multiplexed RNAscope fluorescence *in situ hybridisation* (FISH) to confirm expression of *Cacna1g* in leucine-sensing POMC neurons. As expected, MBH leucine administration significantly increased the proportion of *cfos*^+^ and *cfos*^+^*/Pomc*^+^ neurons (Figure S1G-S1H). Strikingly, 80% of *Cacna1g*^+^/POMC^+^ neurons were activated following MBH leucine administration (Figure 1C-1D), confirming that *Cacna1g* expression is enriched in leucine-activated hypothalamic POMC neurons. *Cacna1g* expression itself was not altered by MBH leucine (Data not shown).

Little is known about the functional relevance of Cav3.1 in POMC neurons, but a recent study suggests that Cav3.1 forms a functional complex with TRPC5 to mediate leptin-induced activation of POMC neurons ^12^. Intriguingly, *Trpc5* is also enriched leucine-activated neurons in our PhosphoTRAP assay (Figure 1B-Table S3). Using RNAscope FISH, we found that ~50% of POMC neurons express *Cacna1g* and strikingly, ~90% *Cacna1g*^+^ POMC neurons co-express *Trpc5* (Figure 1E-1F). *Cacna1g*^+^/*Trpc5*^+^ POMC neurons were observed throughout the entire rostrocaudal span of the ARH (Data not shown), and the colocalisation between *Cacna1g*^+^ and *Trpc5*^+^was markedly less pronounced in non-POMC neurons (Figure S1I). To expand on previous work suggesting that Cav3.1 physically interacts with TRPC5 ^12^, we used HEK293 cells heterologously expressing human Cav3.1 and confirmed direct interaction with human TRPC5 (Figure S1J). Thus, Cav3.1 may functionally contribute to the regulation of POMC neuronal activation.

### Cav3.1 activity is required for leucine-induced activation of POMC neurons

**Figure 2:**
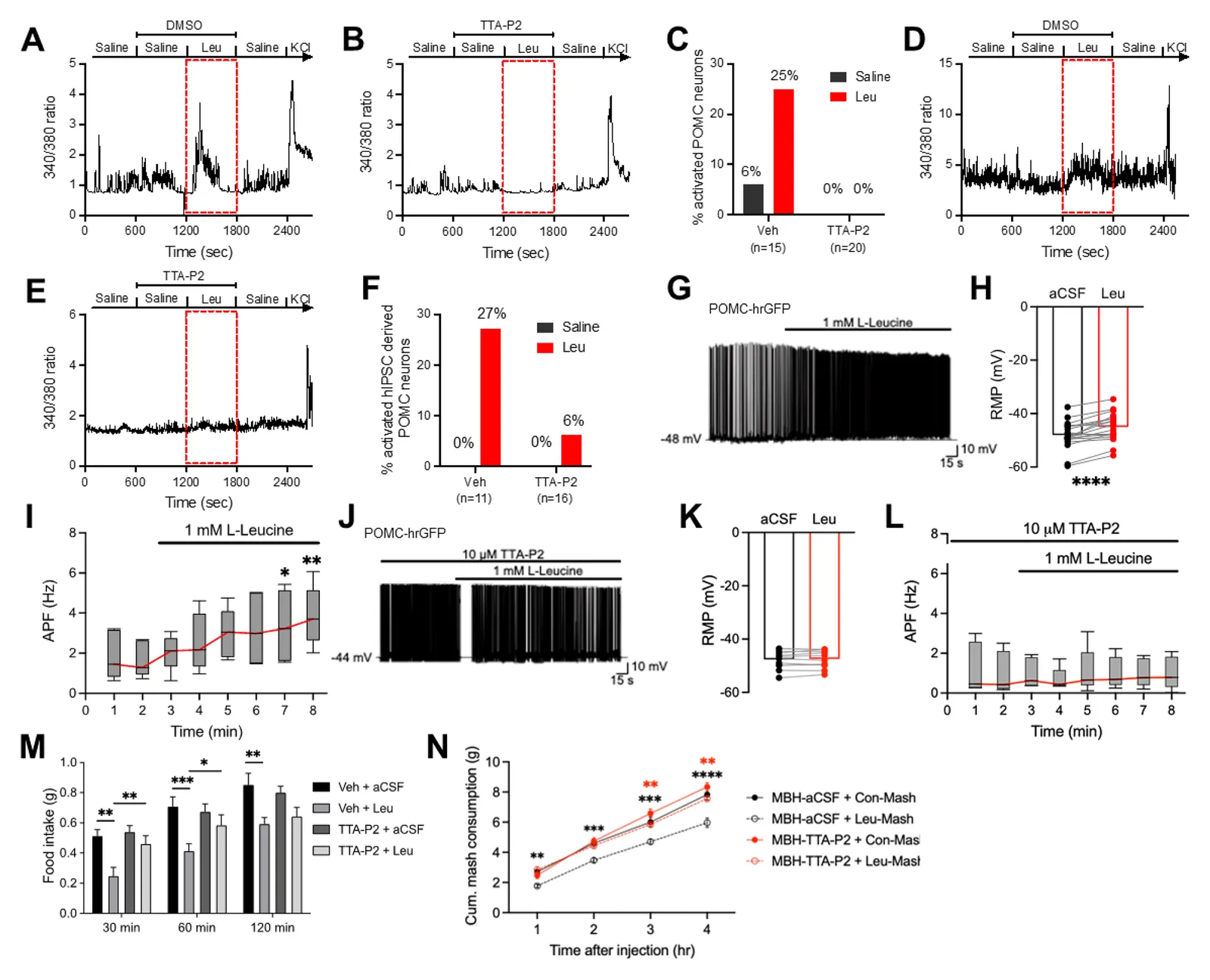
Cav3.1 activity is required for leucine-induced activation of POMC neurons Ca^2+^ imaging assay on mouse primary hypothalamic POMC neurons **(A-C)** and hIPSC-derived POMC neurons **(D-F)**. Representative Ca^2+^ traces of a POMC neuron activated by leucine (1 mM) and co-treated with vehicle **(A**,**D)** and Cav3.1 inhibitor TTA-P2 (10 μM) **(B**,**E)**, and associated quantifications of activated POMC neurons recorded **(C**,**F**; 4 independent experiments**). (G-I)** Brain slice current-clamp recordings of arcuate POMC neurons with leucine treatment. (**G**) Representative current-clamp trace depicting a characteristic depolarization of arcuate POMC neurons by 1 mM leucine. (**H**) Acute effect of leucine on the membrane potential of POMC neurons (n=19). (**I**) Action potential frequency (ARF) in a subpopulation of POMC neurons over time (n=6). **(J-L)** Brain slice current-clamp recordings of arcuate POMC neurons with TTA-P2 (10 μM) pre-treatment prior leucine. **(J)** Representative trace showing that 1 mM leucine fails to induce a depolarization of POMC neurons exposed to TTA-P2. **(K)** Acute effect of leucine on the membrane potential of POMC neurons in the presence of TTA-P2 (n=12). **(L)** APF of POMC neurons in the presence of TTA-P2 over time (n=6). **(M**) Acute feeding response after MBH leucine and vehicle or TTA-P2 co-treatment, n = 10 per group. **(N)** Feeding responses after MBH TTA-P2 (10 μM) injection in awake mice fed a leucine-supplemented mash (31.5 mg/g). Cumulative consumption of control (Con-) and leucine-supplemented (Leu-) mashes, n = 6 per group. Values in (H,K,M,N) are as mean ± SEM. Values in box plots (L,M) are median ± range and comparisons were made versus the minute bin prior leucine treatment. *p<0.05, **p<0.01, ***p<0.001, ****p<0.0001. Statistics: paired t-test: H,K; Friedman test followed by Dunn’s post-test: I,L; repeated 2-way ANOVA followed by Holm-Sidak post-test: M,N. **See also** Figure S2.

We first used TTA-P2, an established pharmacological Cav3.1 inhibitor ^13^, to test the role of Cav3.1 in leucine-induced activation of POMC neurons. Using our previously established Ca^2+^ imaging setup on acute primary culture of adult hypothalamic neurons ^6^, we found that ~25% POMC neurons (as marked by EGFP) are activated by acute leucine treatment, but TTA-P2 pre-treatment completely negated this response (Figure 2A-2C). Likewise, TTA-P2 pre-treatment markedly blunted leucine-induced neuronal activation in human hypothalamic POMC neurons derived from human induced pluripotent stem cells (Figure 2D-2F). Next, we performed brain slice electrophysiological recordings on mouse POMC neurons. In line with previous reports ^5,14^, leucine treatment significantly depolarised POMC neurons, resulting in increased spontaneous action potential firing rate (Figure 2G-2I). Co-treatment with TTA-P2 abolished leucine’s effects (Figure 2J-2L). To test if TRPC5 is also involved in leucine-induced activation of POMC neurons, we used brain slices from *Trpc5* knock-out (KO) mice. *Trpc5* KO abolished leucine-induced depolarisation of POMC neurons (Figure S2A-S2C), suggesting that Cav3.1 and TRPC5 work as a functional complex to mediate leucine-induced activation of POMC neurons. Last, we tested if Cav3.1 activity is required for the anorectic response to MBH leucine injection in behaving animals using a TTA-P2/Leucine co-injection paradigm. As expected, MBH leucine suppressed appetite, and this effect was blunted with TTA-P2 co-treatment (Figure 2M).

To assess the physiological relevance of these findings, we optimised a feeding paradigm where dietary leucine supplementation suppresses dark-onset food intake and increases hypothalamic POMC neuron activation (Figure S2D,S2E,S2G-S2I), validating the anorectic effects of dietary leucine. During a subsequent dark-onset feeding test with access to both control food and leucine-supplemented food, mice equally consumed both diets, ruling out the contribution of palatability and avoidance effects (Figure S2F). Next, we combined MBH TTA-P2 injections with this feeding tests using leucine-supplemented diet. We found that MBH Cav3.1 inhibition blunts the anorectic effect of dietary leucine supplementation (Fig. 2N, S2J).

### Cav3.1 is a leucine sensor

**Figure 3:**
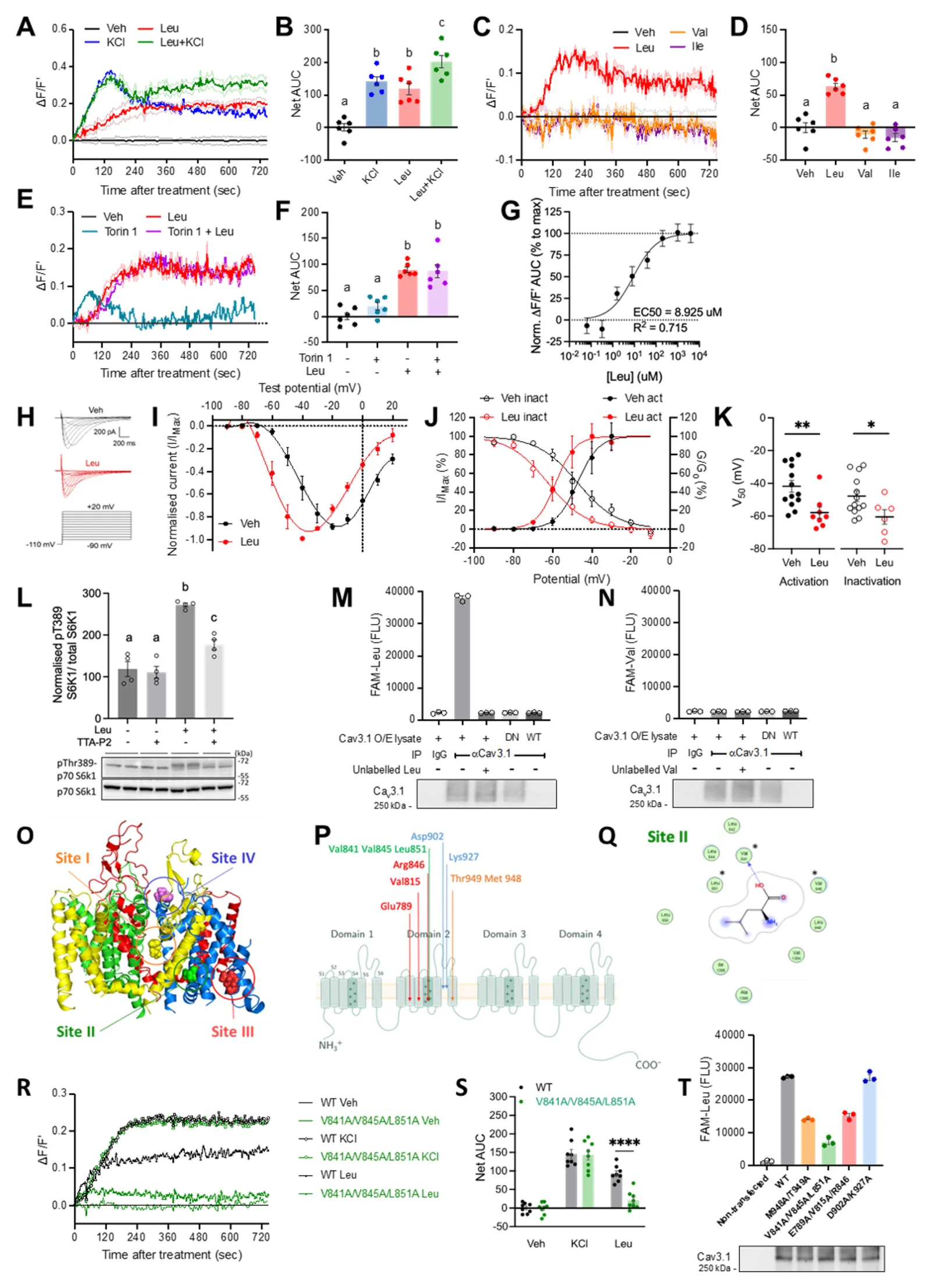
Cav3.1 is a leucine sensor **(A-G)** Fluo8 Ca^2+^ flux assay of HEK293-hCav3.1 cells. Ca^2+^ responses and AUC quantification after KCl (50 mM) and Leu (1 mM) **(A-B)**; Leu, Val and Ile treatments (1 mM each) **(C-D)**, Leu (1 mM) with or without mTOR inhibitor Torin 1 (100 nM) pre-treatment **(E-F)**, n = 6 per group merged from 2 independent experiments with triplicates each. **(G)** Normalised dose-dependent Leu-induced Ca^2+^ response (AUC) curve, n=8 per group, merged from 4 independent experiments with duplicates each. **(H-K)** Whole-cell patch-clamp recording of HEK293-hCav3.1 cells treated with leucine. **(H)** Representative traces of Ca^2+^ currents recorded in vehicle solution (left) and after application of 1 mM leucine. In both cases, cells were held at -100 mv and increasing steps of 10 mV were applied from -90 to +20 mv. **(I)** Normalised current-voltage (I-V) curves of HEK293-hCav3.1 cells treated with vehicle or leucine under the activation protocol. Stead-state activation (G/G_max_) and inactivation (I/I_max_) curves **(J)** and V_50_ histogram **(K)** of HEK293-hCav3.1 cells treated with vehicle or leucine. Veh_act: n=13, Leu_act: n=8, Veh_inact: n=13, Leu_inact: n=6. **(L)** Representative Western blot and densitometry quantification of Thr389 phosphorylation of S6K in HEK293-hCav3.1 cells treated with leucine (1 mM) with or without co-treatment of TTA-P2 (10 μM). n=4 per group. Data were merged from 2 independent experiments with duplicates each. **(M-N)** In vitro binding assay of hCav3.1 to fluorescent labelled leucine **(M)** and valine **(N)**. Post-assay Western blots below the plots validate successful immunoprecipitation (IP). IgG: unimmunized rabbit IgG control; DN: heat-denatured cell lysate prior IP; WT: cell lysate from non-transfected HEK293 cells. Data represent 3 technical replicates upon fluorescence measurements. These experiments have been repeated twice with similar results. Data of representative sets of experiment are shown. **(O)** Human Cav3.1 protein (PDB id: 6KZO) is shown in cartoon representation with Domain I, II, III and IV coloured in red, light blue, yellow and green, respectively. The representative binding poses of L-leucine are shown in sphere representation with poses relevant to different sites coloured distinctly: yellow (putative site I, marked with an orange circle), green (putative site II, marked with a green circle), red (putative site III, marked with a red circle) and light pink (putative site IV, marked with a blue circle). **(P)** Schematic representation of hCav3.1 critical amino acid residues of corresponding predicted leucine binding sites chosen for mutagenesis experiments, colour-coded as in (O). **(Q)** 2D ligand interactions diagrams for the docked poses of leucine at Site II. The residues mutated for experimental validation are shown with asterisks. The dotted arrow signs indicate hydrogen bonding whilst the dotted contour around the ligand pose indicate hydrophobic interactions with residues shown. These diagrams were generated in MOE. **(R-S)** Fluo8 Ca^2+^ flux assay of HEK293-hCav3.1 Site II mutant (V841A/V845A/L851A) cells. Ca^2+^ responses **(R)** and AUC quantification **(S)** after KCl and Leu treatments. n=8 merged from 4 independent experiments with duplicates each. **(T)** In vitro fluorescent leucine binding assay of WT and Site I–IV hCav3.1 mutants. Post-assay Western blot below the plot validates successful IP. Data represent 3 technical replicates upon fluorescence measurements. These experiments have been repeated twice with similar results. Data of a representative experiment is shown. Values and Ca^2+^ response traces are reported as mean ± SEM. *p<0.05, **p<0.01, ****p<0.0001. Groups denoted with different letters in (B,D,F,L) indicate significant difference (p<0.05). Statistics: 1-way ANOVA with Holm-Sidak post-test: B,D,F,L; least-squares regression with variable slopes: G; paired t-test: K; unpaired t-test: S. **See also** Figure S3.

To test the possibility that leucine might directly interact with Cav3.1 and modulate its function, we used HEK293 cells stably expressing human Cav3.1 (HEK293-hCav3.1). In HEK293-hCav3.1 cells loaded with cell-permeable Ca^2+^ sensitive Fluo8-AM dye, we confirmed dose-dependent activation of hCav3.1 by potassium chloride (KCl)-induced depolarisation (Figure S3A-S3C). Leucine treatment significantly activated hCav3.1 and enhanced KCl-induced hCav3.1 activation (Figure 3A-3B). These effects were not observed in wild-type HEK293 cells (Figure S3D), suggesting that leucine has a direct effect on Cav3.1 function. Activation of hCav3.1 is specific to leucine among all branched-chain amino acids (Figure 3C-3D), and is mTOR independent (Figure 3E-3F). Importantly, we found leucine activated hCav3.1 Ca^2+^ response in a dose-dependent manner with a EC50 (half-maximal effective concentration) at 8.925 µm (Figure 3G, S3E) – within the physiological range of CSF leucine concentration (7-12 µm)^5^. In contrast, hTRPC5-expressing stable HEK293 cells, while robustly responding to the acetylcholine receptor agonist carbachol (CCH), failed to respond to leucine (Figure S3F-S3I), suggesting that TRPC5 is recruited downstream from Cav3.1 to further activate POMC neurons after leucine sensing.

We then performed whole-cell voltage-clamp recordings on HEK293-hCav3.1 to elucidate the electrophysiological mechanisms through which leucine affects Cav3.1 function. In control cells, the activation (V50_act_Con_: -41.78 ± 12.83 mV) and inactivation curves (V50_act_Con_: -47.77 ± 11.91 mV) were comparable with the published literature ^15^. Leucine treatment produced a significant left-shift of both the activation (V50_act_Leu_: -57.42 ± 11.18 mV) and inactivation curves (V50_inact_Leu_: -60.50 ± 11.01 mV) (Figure 3H-3K). The resting membrane potential of wild-type HEK293 cells has been reported to be at about -30 to -50 mV ^16^. Importantly, according to our data, half of hCav3.1 are activated at this voltage band in vehicle-treated cells, while with leucine treatment hCav3.1 is fully activated (Figure 3J).

Previously, it has been demonstrated that mTOR signalling is required for the appetite-suppressive effects of MBH leucine *in vivo* ^8^, and work from others showed that Ca^2+^ influx activates mTOR in a calmodulin/hVps34 dependent manner ^11^, which are ubiquitously expressed and has been detected in human hypothalamus and HEK293 cells (Human Protein Atlas). Accordingly, to test the prediction that leucine can activate mTOR signalling via Cav3.1, we used HEK293-Cav3.1 cells, which show robust Thr389 phosphorylation or S6K in response to leucine treatment. TTA-P2 significantly blunted leucine-induced activation of the mTOR signalling pathway (Figure 3L), suggesting that leucine sensing via Cav3.1 contributes to leucine-induced mTOR activation. These data support a role for Cav3.1 as a leucine sensor upstream from mTOR and mediating leucine-induced activation of POMC neurons and mTOR signalling.

Given that mTOR signalling is unlikely to mediate leucine-induced Cav3.1 activation (Figure 3E-3F) ^6^, and that Cav3.1 phosphorylation does not change its voltage dependence ^15^, we reasoned that leucine might directly bind to Cav3.1 to modulate its activity. To test this idea, we adopted an immunoprecipitation-based *in vitro* leucine binding assay, previously used to test the leucine-binding property of the leucine sensor Sestrin2^17^. Using fluorescently-labelled leucine (FAM-leucine), we detected marked binding of FAM-leucine with hCav3.1, out-competed following incubation with excess amounts of unlabelled leucine, indicating that leucine can directly bind to hCav3.1 (Figure 3M). Moreover, heat-denaturation of hCav3.1 abolished FAM-leucine binding, indicating that intact protein folding of hCav3.1 is needed for leucine binding (Figure 3M). Of note, FAM-valine failed to bind to hCav3.1, suggesting a leucine-specific binding mechanism (Figure 3N).

To identify leucine’s binding site on hCav3.1, we performed a domain mapping experiment by generating a series of non-overlapping truncated hCav3.1 based on its 4 distinct transmembrane Domains, and tested them using the FAM-leucine binding assay. Unexpectedly, none of the 4 Domains alone bound leucine to a comparable extent of the full-length hCav3.1 (Figure S3J-S3L), implying that the leucine binding site may involve intact 3D structure. We next performed an *in silico* leucine docking analysis using the recently cryo-EM-resolved 3D structure of the human Cav3.1 protein (PDB id: 6KZO) ^18^. We first performed blind docking of L-leucine as the active ligand and with L-valine as a decoy against the entire hCav3.1 structure. After subsequent focused docking, we shortlisted four plausible leucine binding sites (hereafter termed Site I-IV) for further study (Figure 3O-3Q, S3O, S3R, S3U). To test the importance of these sites in leucine binding and effects on leucine-induced hCav3.1 activation, we performed site-directed mutagenesis of the critical residues on these sites to alanine (Figure 3P), generated HEK293 stable cell lines expressing these mutated hCav3.1 channels and used Western blot to confirm their expression (Figure S3M), and then investigated these using the Fluo8-based Ca^2+^ flux and *in vitro* FAM-leucine binding assays. The Site IV mutant (D902A/K927A) did not affect the channel function (Figure S3U-S3W) nor its binding to FAM-leucine (Figure 3T). Site I (M948A/T949A) and Site III (E789A/V815A/R846A) mutants did not respond to KCl or leucine (Figure S3P-S3Q, S3S-S3T), and had markedly impaired leucine binding (Figure 3T), suggesting major alterations to the protein structure affecting both normal voltage-sensing and leucine binding. In contrast, the Site II mutant (V841A/V845A/L851A) showed comparable response to KCl as WT, but leucine-induced Ca^2+^ influx was significantly blunted (Figure 3R-3S), and binding affinity to FAM-leucine was the lowest (^~^-75% vs. WT) (Figure 3T). Thus, Site II - a cytoplasm-facing hydrophobic pocket formed by the two voltage-sensing S4 segments of Domain II and III - is a functionally critical leucine binding site. Additionally, amino acid sequence alignments show that these two S4 segments of hCav3.1 are fully conserved among mammals but not with zebrafishes nor with hCav3.2 and hCav3.3 (Figure S3N), suggesting Cav3.1-mediated leucine sensing mechanism may be evolutionally conserved in mammals.

### Mediobasal hypothalamic *Cacna1g* is required for the appetite and weight-suppressing effect of dietary proteins

**Figure 4:**
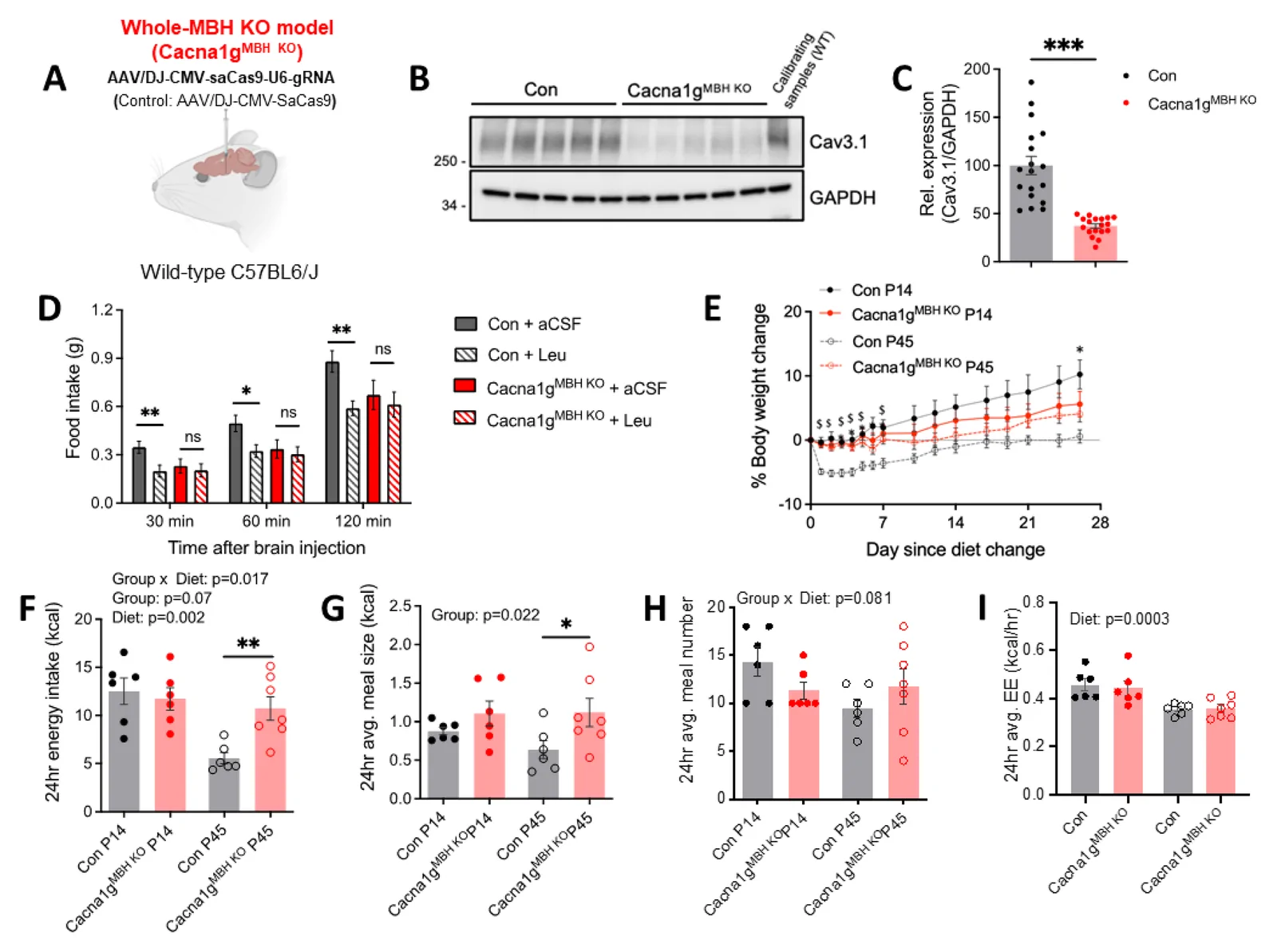
Mediobasal hypothalamic *Cacna1g* is required for the appetite- and weight-suppressing effect of dietary proteins **(A)** Cacna1g^MBH KO^ model. **(B-C)** Western blot analysis of Cav3.1 expression in Cacna1g^MBH KO^ mice with a C-terminal specific Cav3.1 antibody. Representative image **(B)** and densitometric quantification **(C)** of the blots. n = 18 per group. **(D)** Acute feeding response after MBH leucine injection in Con and Cacna1g^MBH KO^. Con: n = 11; MBH KO: n=12. **(E) %** body weight change of Con and Cacna1g^MBH KO^ over the course of 4 weeks feeding on 14% normal protein (P14) diet and 45% hight protein (P45) diet. **(F-I)** Metabolic and feeding behaviour assessments of Con and Cacna1g^MBH KO^ mice on P14 and P45 diets during indirect calorimetry measurement. 24-hr total energy intake **(F)**, 24-hr average meal size **(G)**, 24-hr average meal number **(H)** and 24-hr average energy expenditure **(I)**. Con-P14: n = 6; Con-P45: n = 6; KO-P14: n = 7; KO-P45: n = 7. Values are reported as mean ± SEM. *p<0.05, **p,0.01, ***p<0.001. Statistics: unpaired t-test: C; repeated 2-way ANOVA with Holm-Sidak post-test: D,E; 2-way ANOVA with Holm-Sidak post-test: F-I. **See also** Figure S4 & S5 and Table S7.

Hypothalamic leucine sensing has been proposed to contribute to the appetite-suppressing effect of dietary proteins, but evidence supporting causality is lacking. To address this, we developed an AAV-based CRISPR mediated knock-out of *Cacna1g* in the MBH (hereafter termed Cacna1g^MBH KO^) (Figure 4A, S4A-S4B). We validated expected indel mutations and top predicted off-target genes in mouse hypothalamic mHypoE-N46 cells and hypothalamic tissues (Figure S4C-S4E). Western Blot analysis using an anti-Cav3.1 C-terminal specific antibody revealed a 64% KO efficiency in the MBH of Cacna1g^MBH KO^ mice (Figure 4B-4C).

Consistent with the effect of MBH pharmacological inhibition of Cav3.1 with TTA-P2, MBH leucine failed to reduce food intake in Cacna1g^MBH KO^ mice (Figure 4D), indicating that Cav3.1 activation is required for leucine-induced anorexia. To determine if leucine sensing through Cav3.1 contributes to the feeding and metabolic responses to high-protein diets, we transitioned Cacna1g^MBH KO^ and control mice to either a 45% protein diet (high protein content, hereafter termed P45) or a 14% protein diet (normal protein content, hereafter termed P14) (Table S6). Over the course of 4 weeks, P45 feeding significantly reduced weight gain in control mice, but this effect was blunted in Cacna1g^MBH KO^ mice (Figure 4E). Decreased weight gain in the control group under P45 feeding was accounted for by a significant reduction in energy intake during the active phase through a specific decrease in meal size, which failed to occur in Cacna1g^MBH KO^ mice (Figure 4F-4H, S4F, S4M). P45 feeding significantly reduced energy expenditure but no difference was observed between Cacna1g^MBH KO^ and control groups (Figure 4I, S4G-S4H, S4N-S4O). We did not detect overt differences in the respiratory exchange ratio (RER) (Figure S4I, S4P) and locomotor activity (Figure S4J, S4Q) among control and Cacna1g^MBH KO^ mice under both feeding paradigms. Glucose tolerance was slightly increased in P14-fed Cacna1g^MBH KO^ mice (Figure S4K, S4R) but insulin sensitivity not affected (Figure S4L, S4S). Post-absorptive plasma amino acid concentrations and urinary markers of nitrogen disposal were markedly affected by high protein feeding but did not overtly differ between groups (Figure S4T-S4AA and Table S7). Together, these data revealed MBH Cav3.1 mediates protein-induced satiation and is required for the weight-suppressive effect of high protein feeding.

Because whole-body *Cacna1g* KO protects against diet-induced obesity (DIO) ^19^, we tested the role of MBH Cav3.1 in DIO. Weight gain (Figure S5A-S5C), energy intake (Figure S5D-S5G) and expenditure (Figure S5H-S5I), RER (Figure S5J) and locomotor activity (Figure S5K) and did not overtly differ between Cacna1g^MBH KO^ and WT controls during 60% high-fat diet maintenance. However, post-mortem RT-qPCR analysis revealed that MBH *Cacna1g* expression was significantly downregulated following 8-weeks HFD feeding in control mice (Figure S5L), which might explain the lack of phenotypic difference between Cacna1g^MBH KO^ and WT mice under these conditions. Overall, these data imply that MBH Cav3.1 is not involved in the protective effects of whole-body Cacna1g KO against DIO, and suggest that HFD feeding may blunt Cav3.1-mediated leucine sensing in the MBH.

### *Cacna1g* in hypothalamic POMC neurons is required for the appetite-suppressing effect of dietary proteins

**Figure 5:**
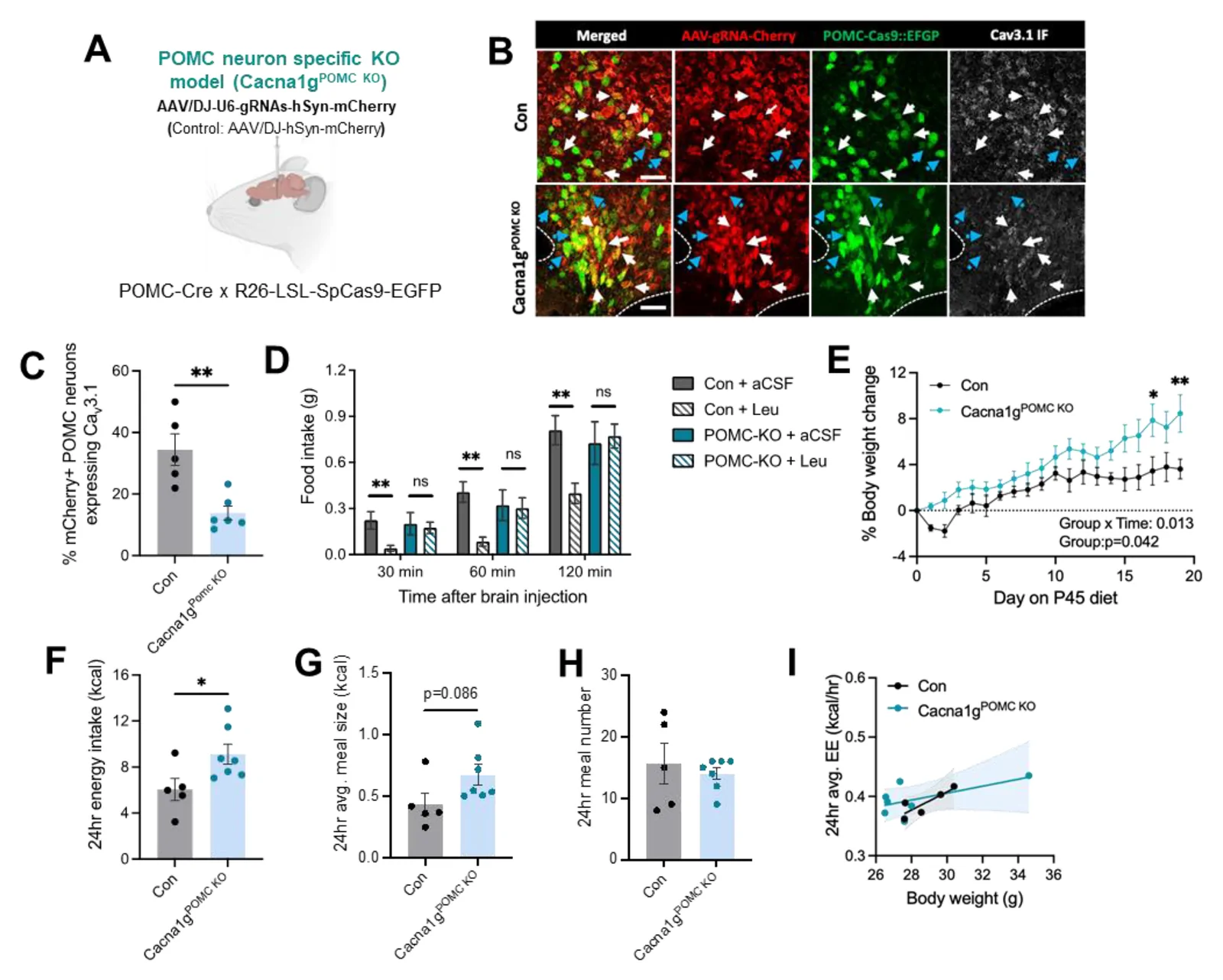
*Cacna1g* in hypothalamic POMC neurons is required for the appetite- suppressing effects of high protein diet feeding **(A)** Cacna1g^POMC KO^ model. **(B-C)** Confocal **i**mmunofluorescence (IF) microscopy analysis of the CRISPR knockout efficiency of Cav3.1 in Cacna1g^POMC KO^ mediobasal hypothalamus with a C-terminal specific Cav3.1 antibody validated in Fig. S6. Zoom-in images of the regions highlighted with white squares in Fig. S6F **(B)**. White arrows with solid line: example POMC+/mCherry+/Cav3.1+ neurons. Blue arrows with broken line: example POMC+/mCherry+/Cav3.1- neurons. Scale bar: 50 µm. Quantification for % mCherry+ transduced POMC neurons expressing Cav3.1 **(C)**. Con: n = 5; Cacna1g^POMC KO^: n = 6. **(D)** Acute feeding response after MBH leucine injection in Con and Cacna1g^POMC KO^. Con: n = 10; KO: n=8. **(E) %** body weight change of Con and Cacna1g^POMC KO^ over the course 3 weeks feeding on P45 diet. **(F-I)** Metabolic and feeding behaviour assessments of Con and Cacna1g^POMC KO^ mice on P45 diets during indirect calorimetry measurement. 24-hr total energy intake **(F)**, 24-hr average meal size **(G)**, 24-hr average meal number **(H)** and ANCOVA analysis of average 24hr energy expenditure against body weight **(I)**. Con: n = 5; Cacna1g^POMC KO^: n = 7. Values are reported as mean ± SEM. *p<0.05, **p,0.01. Statistics: unpaired t-test: C, F-H; repeated 2-way ANOVA with Holm-Sidak post-test: D, E; ANCOVA: I. **See also** Figure S6.

ARH POMC neurons suppress feeding and are activated by dietary leucine (Figure S2G-S2I) and local leucine infusion (Figure 1C, S1G) ^5,8^. To test if the feeding phenotype observed in Cacna1g^MBH KO^ mice is mediated via Cav3.1 activity in ARH POMC neurons, we used a CRISPR strategy to knockout *Cacna1g* specifically in ARH POMC neurons by stereotactical injection of gRNAs-expressing AAV to the MBH of transgenic mice with SpCas9 expression exclusively in POMC neurons (hereafter termed Cacna1g^POMC KO^, Figure 5A, S6A-S6C). POMC-specific KO of *Cacna1g* was validated using a Cav3.1-specific antibody ^20^ (Figure S6D-S6E) and found a ^~^60% reduction in Cav3.1 expression in mCherry-expressing transduced POMC neurons in Cacna1g ^POMC KO^ mice (Figure 5B-5C, S6F).

POMC-specific KO of *Cacna1g* was sufficient to blunt the anorectic effect of MBH leucine injection (Figure 5D). During maintenance on P45, Cacna1g^POMC KO^ mice gained significantly more weight compared to controls (Figure 5E), which was mediated by an increase in energy intake during the active phase (Figure 5F, S6G) and a trend towards increased meal size but no difference in meal number (Figure 5G-5H). Energy expenditure (Figure 5I, S6H), RER (Figure S6I), locomotor activity (Figure S6J), glucose tolerance (Figure S6K) and urinary parameters (Figure S6L-S6N) did not differ between groups. Thus, Cacna1g^POMC KO^ mice broadly phenocopied observations seen in Cacna1g^MBH KO^ mice during high protein diet feeding, supporting the conclusion that Cav3.1 in POMC neurons is required for the appetite-suppressing effect of high protein feeding.

### Pharmacological activation of hypothalamic Ca_v_3.1 reduces appetite and promotes weight loss in lean mice

**Figure 6:**
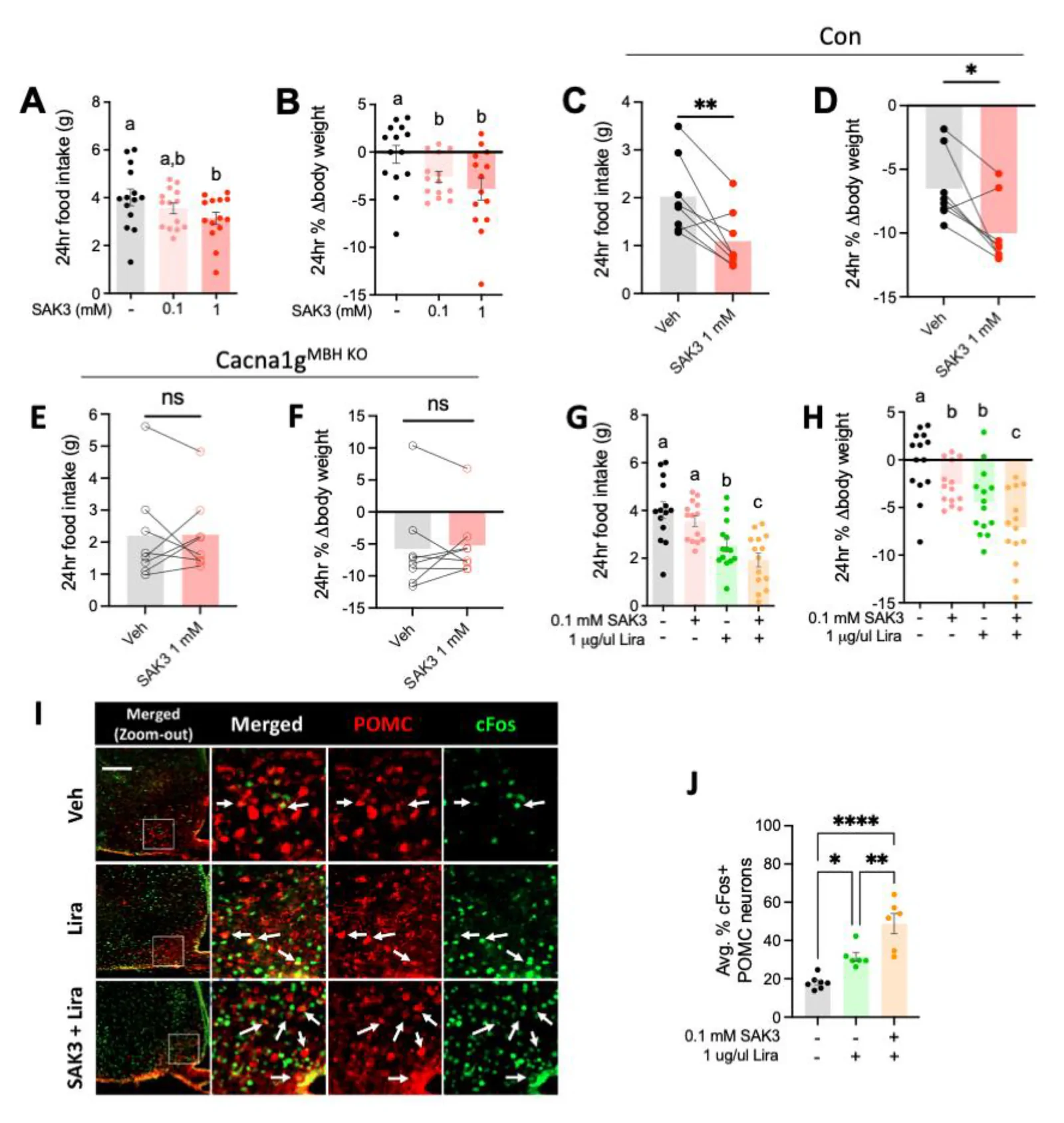
Pharmacological activation of hypothalamic Cav3.1 reduces appetite and promotes weight loss in lean mice **(A-B)** 24-hr feeding responses **(A)** and % body weight changes **(B)** after MBH injection of Veh, 0.1 mM and 1 mM Cav3.1 activator SAK3. n = 14 per group. **(C-F)** Hypothalamic Cav3.1 is required for the feeding effects of SAK3. 24-hr feeding responses and % body weight changes after MBH injection of Veh and 1mM SAK3 in Con (n = 6) **(C-D)** and Cacna1g^MBH KO^ mice (n = 8) **(E-F). (G-H)** 24-hr feeding responses **(G)** and % body weight changes **(H)** after MBH injection of Veh, 0.1 mM SAK3, 1 μg/μl liraglutide (Lira) and SAK3+Lira co-treatment, n = 14 per group. **(I-J)** Immunofluorescence (IF) microscopy analysis of POMC neuron activation after Lira and SAK3+Lira. Representative fluorescence images of POMC (red) and cFos (green) **(I)**. White squares: regions shown in the zoom-in images. White arrows: neurons double positive for POMC and cFos IF. Scale bar: 200um. Quantification of images analysis **(J)**. Values are reported as mean ± SEM. *p<0.05, **p<0.01, ****p<0.0001. Groups denoted with different letter in (A, B, G, H) indicate significant difference (p<0.05). Statistics: repeated 1-way ANOVA with Holm-Sidak post-test: A,B,G,H; paired t-test: C-F; 1-way ANOVA with Holm-Sidak post-test: J. **See also** Figure S7.

We explored the potential of Cav3.1 as a weight loss target using SAK3, a novel small molecule pharmacological activator of Cav3.1 ^21^. MBH injection of SAK3 reduced 24-hr food intake and body weight gain in a dose-dependent manner (Figure 6A-6B, S7A). This effect is lost in Cacna1g^MBH KO^ mice (Figure 6C-6F), confirming that Cav3.1 activation mediates SAK3 action.

Hypothalamic POMC neurons contribute to the anorectic action of the FDA-approved weight loss drug liraglutide, a GLP-1 receptor agonist, via TRPC5 activation ^22,23^. Thus, we reasoned that Cav3.1 activation might potentiate liraglutide-induced POMC activation. To test this, a subthreshold dose of SAK3 was delivered into the MBH during systemic liraglutide treatment. As predicted, MBH SAK3 increased the anorectic and weight-suppressive effect of liraglutide (Figure 6G-6H, S7B). Immunohistochemistry imaging analysis confirmed that SAK3 increased liraglutide-induced activation of POMC neurons under these conditions (Figure 6I-6J). Likewise, subthreshold SAK3 increased the appetite- and weight-suppressive action of leptin (Figure S6C-S6E) and the 5HT-receptor 2C agonist lorcaserin (Figure S6F-S6H), which both signal through POMC neurons to regulated appetite and energy balance. Thus, MBH Cav3.1 plays a role in integrating satiety signals *in vivo* and might represent a relevant weight loss target.

### Intranasal administration of Cav3.1 activator as an anti-obesity treatment

**Figure 7:**
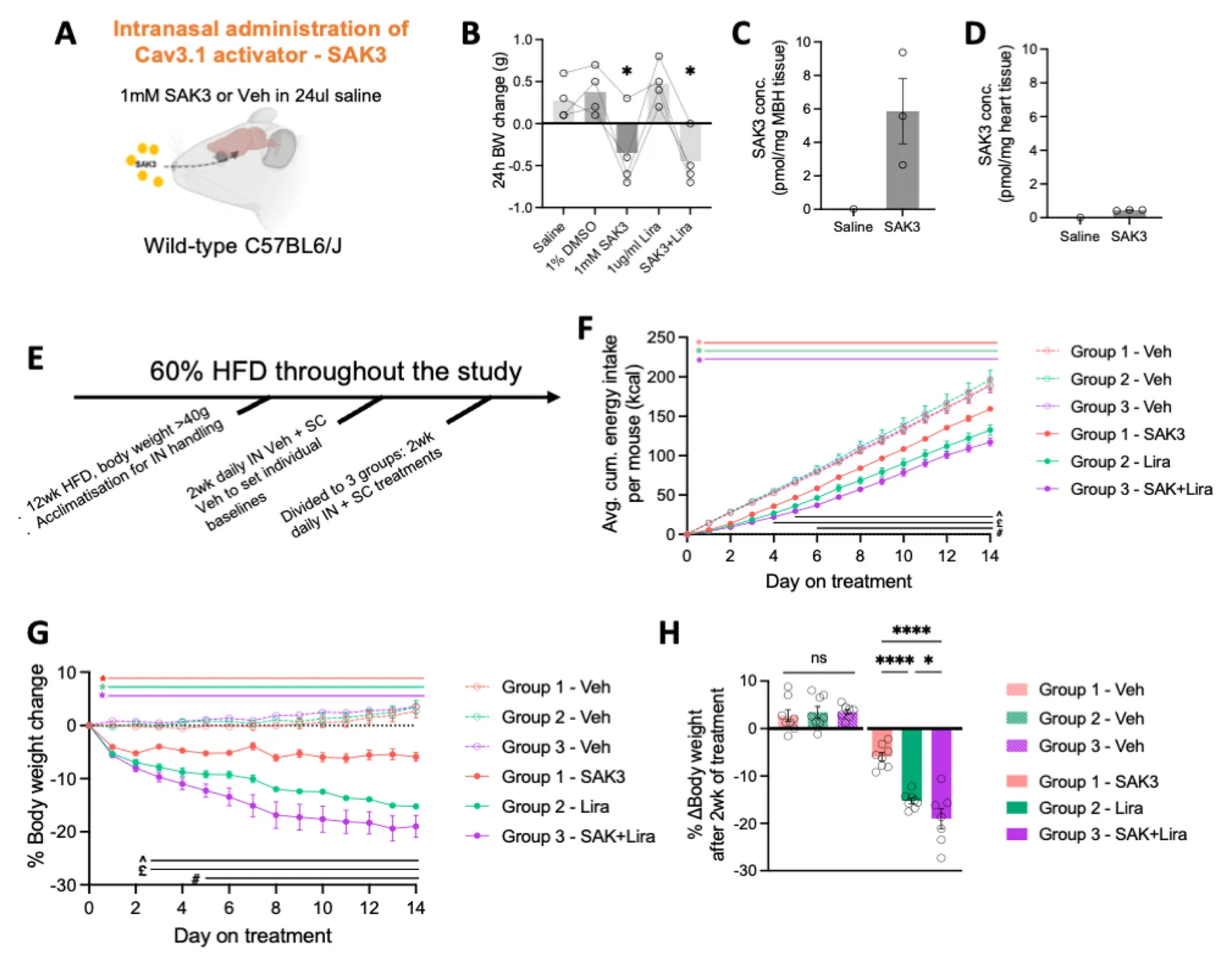
Intranasal administration of Cav3.1 activator as an anti-obesity treatment **(A)** Diagram of experimental strategy using intranasal administration (IN) of SAK3 to activate hypothalamic Cav3.1 activity. (B) 24-hours body weight change after IN administration of indicated substances on lean mice fed on normal chow. Comparisons were made against saline-treated condition. Each treatment were 96 hours apart. n = 4 per repeated testing condition. **(C-D)** Mass spectrometry quantification of the tissue abundance of SAK3 in the MBH (**C**) and heart (**D**) 1 hour after IN administration. n = 1 for IN saline; n = 3 for IN SAK3. **(E)** Diagram of the experimental paradigm to test the weight loss effect of IN SAK3 in high fat diet induced obesity mouse model, n = 8 per group for (F-H). **(F)** Cumulative energy intake since the start of respective treatments over the course of 2-week treatment. **(G-H)** % Body weight change normalised to the start of respective treatments over the course of 2-week treatment **(G)**. Histogram summarising the terminal % body weight change **(H)**. Values are reported as mean ± SEM. *p<0.05, ****p<0.0001. For (F-G), on the top over the plots, colour-coded * indicates p<0.05 between respective Drug vs Veh treatment phases within the same group; at the bottom of the plots, ^p<0.05 SAK3 vs Lira; ^£^p<0.05 SAK vs SAK3+Lira; ^#^p<0.05 Lira vs SAK3+Lira. Statistics: repeated 1-way ANOVA with Holm-Sidak post-test: B; repeated 2-way ANOVA with Holm-Sidak post-test: F,G; 1-way ANOVA with Holm-Sidak post-test: H.

We tested the potential weight loss benefits of MBH Cav3.1 activation with SAK3 in diet-induced obese mice using a non-invasive drug delivery method to the central nervous system via the intranasal (IN) route (Figure 7A) ^23^. In a pilot experiment, we found that IN delivery of SAK3 induced weight loss in lean mice maintained on chow diet overnight (Figure 7B), with no obvious adverse effects. We developed a mass spectrometry-based method to detect SAK3 abundance in tissues and confirmed presence of SAK3 in the MBH 1 hour after IN SAK3 dosing (Figure 7C), demonstrating the feasibility of using the IN route to deliver SAK3 to the MBH. We could also detect SAK3 in heart tissues but at concentrations ^~^13-fold lower than in the MBH (within the femtomolar range) (Figure 7D). Next, we tested the weight loss effect of IN SAK3 in diet-induced obese mice (Figure 7E). Daily IN SAK3 dosing for 2 weeks significant reduced food intake (Figure 7F), leading to significant weight loss, and significantly increased the appetite- and weight-suppressive action of liraglutide (Figure 7F-7H). Collectively, these results support the feasibility of targeting MBH Cav3.1 via the intranasal route as a weight loss strategy to enhance the weight loss response to GLP-1 receptor agonism.

## Discussion

In this study, we reveal a role for Cav3.1 as a neuronal leucine sensor mediating the anorectic action of hypothalamic leucine and high protein feeding. We found that *Cacna1g* expression is highly enriched in MBH neurons activated by leucine, about fourfold over non-responsive neurons. *Cacna1g* is not only a marker for leucine-sensing neurons, but also required for the rapid expression of Ca^2+^ currents in response to increased leucine levels and leucine-induced activation of MBH POMC neurons. Mechanistic investigations identified Cav3.1 as a specific mediator of leucine-induced neuronal activation. Leucine enhances Cav3.1 voltage sensitivity, lowering the activation threshold of the channel, and this occurs independent of and upstream from mTORC1 signalling. We demonstrate the role of Cav3.1 in the appetite-suppressive action of high protein feeding, and validates its relevance as an anti-obesity target.

A major finding of this study is the identification of Cav3.1 as a direct leucine sensor. Combining *in silico* analyses, *in vitro* binding, and functional assays, we reveal that leucine (but not valine or isoleucine) binds to the hydrophobic pocket located between the voltage-sensing S4 segments of Domain II and III of Cav3.1, linking leucine’s action to Cav3.1 voltage sensing. Other amino acids have been shown to modify the activity of voltage-gated ion channels. For example, the modified amino acid GABA directly binds to voltage-gated potassium channels KCQN3 and KCNQ5 ^24^. Our finding extent these observations and highlight the need to consider how excitable cells might sense external amino acid availability through direct modifications of ion channel properties.

Protein sequence alignment analysis suggests that the role of Cav3.1 as a leucine sensor may be conserved among mammals, since the Cav3.1 Domain 2, required for leucine sensing, is highly conserved. Within the Domain II of Cav3.1, we identified 3 putative leucine binding sites. Mutations of two of the binding sites (Thr949/Thr948 and Glu789/Val815/Arg846) produce channels that are non-responsive to depolarisation, therefore likely produce major conformational disruptions, accounting for the loss of leucine binding and Ca^2+^ responses in these mutants. However, the mutation of the hydrophobic pocket (Val841/Val845/Leu851) within the voltage-sensing S4 leads to specific loss of leucine-induced Ca^2+^ responses and leucine binding, yet retaining intact responsiveness to depolarisation, indicating a major role of this site in interacting with leucine. Among the three members of T-type VGCCs, there is a marked variation in the sequence of the amino acid residues involved in leucine binding, suggesting that the leucine-sensing property of Cav3.1 might be unique among VGCCs, although further study will be needed to confirm this prediction. Our data expand our knowledge on how cells monitor availability of essential nutrients such as leucine, with to date only one other described cell-intrinsic leucine-specific sensing mechanism, Sestrin1/2 ^17,25^.

Our work highlights the role of Cav3.1-mediated leucine sensing in leucine’s anorectic action. MBH neurons exhibit very rapid electrical changes in response to leucine exposure, and Cav3.1-mediated sensing provides a mechanism mediating these immediate neuronal responses. Past work implicates mTORC1 activation in leucine-induced anorexia ^5,8^, but this signalling mechanism impinges on cellular activity over a longer time frame. Here, we show that leucine-induced Cav3.1 Ca^2+^ currents occur independently of mTORC1 signalling but instead contribute to leucine-induced mTORC1 activation. This is consistent with previous work demonstrating that leucine-induced Ca^2+^ currents activate mTORC1 via calmodulin-hVps34 signaling ^11^. Hence, we propose that a multiphasic sequence of events occurs in response to leucine sensing to promote anorexia: leucine acutely activates Cav3.1 which causes the initial depolarisation and primes the subsequent activation of POMC neurons; the slower mTORC1 activation induced by Ca^2+^ influx and Sestrins-dependent mechanism contributes to reinforce the neuronal activation machinery. Of note, a recent study indicates that ARH POMC neurons respond to mTORC1 signalling heterogeneously ^26^, and Cav3.1 activation might differently modify the activity of different subsets of POMC neurons.

Much of the evidence supporting a role for brain leucine sensing in appetite regulation comes from pharmacological studies in which leucine is directly infused into specific brain regions. While these approaches are useful for bypassing peripheral effects of leucine and isolating the direct consequences of its detection in the brain, they do not establish the physiological relevance of brain leucine sensing^27^. Here we provide data supporting the physiological relevance of hypothalamic leucine sensing in appetite control. We show that dietary leucine supplementation supresses food intake and activates anorectic hypothalamic POMC neurons, and these effects are blunted during blockade of MBH Cav3.1 activity. Likewise, POMC Cav3.1 is required for the appetite-suppressive action of high-protein feeding. The fact that Cav3.1 expression is widespread and present outside feeding circuits raises questions about the specificity of this mechanism. However, the ability to sense leucine is likely relevant to many brain circuits not directly involved in appetite control. We know for example that hypothalamic growth and reproductive circuits are sensitive to dietary protein intake and might be tuned by dietary leucine availability ^28^. In line with this argument, brain branched-chain amino acid excess triggers seizures, for example, in patients with maple syrup urine disease ^29^, while Cav3.1 hyperactivity has been found to contribute to epileptic seizures in mouse models and humans ^30,31^. This raises the possibility Cav3.1-mediated leucine sensing might be a shared mechanism through which neurons adapt their activity to dietary protein availability.

Our data support the relevance of Cav3.1 in hyperphagic obesity. We found decreased MBH *Cacna1g* expression in diet-induced obese mice, and efficient appetite suppression following SAK3 treatment, which activated Cav3.1, leading to significant weight loss. Interestingly, genetic obesity in Prader–Willi syndrome (PWS) patients associate with a 62% reduction in hypothalamic Cacna1g expression ^32^, suggesting that this role is conserved. This is consistent with our findings that Cav3.1 activity regulates the activity of both mouse and human iPSC-derived POMC neurons. Thus, deficiency of hypothalamic Cav3.1 activity might be a common pathogenic mechanism contributing to overeating in diet-induced and genetic forms of obesity.

Current incretin-based anti-obesity medications are effective, but 15–20% of patients fail to respond, and adverse effects contribute to high discontinuation rates ^33,34^, often resulting in rapid weight regain. Here, we show that activation of Cav3.1 with SAK3 enhances the weight-reducing effects of GLP-1 receptor agonism, offering a strategy to achieve comparable weight loss with lower doses of GLP-1R agonists. Mechanistically, Cav3.1 interacts with TRPC5 ^12^, a required component in GLP-1R-induced POMC activation ^22,23^, and this interaction enhances TRPC5-induced POMC depolarization, for example in response to leptin ^12^. We found 90% co-expression of *Trpc5* and *Cacna1g* in ARH POMC neurons. TRPC5 is required for leucine-induced activation of POMC neurons, but leucine does not activate TRPC5 directly. Thus, we conclude that while Cav3.1 serves as a leucine sensor, the interaction with TRPC5 is needed to amplify the Ca^2+^ influx and subsequent activation of POMC neurons following leucine exposure. This is consistent with the properties of T-type Ca^2+^ channels, characterised by their low activation threshold and important in setting the excitability of neurons ^35^. The physical interaction of Cav3.1 with TRPC5 uniquely posits Cav3.1 to tune the excitability of POMC neurons to satiety signals ^22,23,36,37^. Consistently, we found that SAK3 potentiates the appetite-suppressive effect of several anorectic molecules which signal in POMC neurons via TRPC5. Together, our data suggest Cav3.1 plays a crucial role in tuning the excitability of POMC neurons in maintaining energy homeostasis.

Importantly, we demonstrated the feasibility of targeting MBH Cav3.1 via intranasal administration, a non-invasive route allowing spatiotemporal control of drug action. Non-polar small molecule drugs like SAK3 are particularly suitable for this delivery route, and the MBH is an ideal target as it is accessed by multiple diffusion pathways ^38,39^.

Finally, although high-protein diets are effective and widely used for body weight management, concerns remain about their long-term safety, particularly given the association between elevated circulating BCAA levels and increased risk of metabolic and cardiovascular diseases ^40–43,44,45^. Intranasal delivery of a Cav3.1 activator offers a novel strategy that harnesses the satiety pathways targeted by dietary proteins to regulate body weight while minimizing side effects.

Altogether, this study expands the current understandings of the molecular biology of cellular amino acid sensing and the neurocircuitry mediating the feeding effects of dietary protein sensing. The application of this knowledge has led to the identification of novel candidate anti-obesity strategies.

### Limitations of Study

While we used multidisciplinary approaches on both mouse and human-derived models to demonstrate that Cav3.1 is required for leucine induced activation of hypothalamic POMC neurons, we only tested the feeding and metabolic consequences of activating this mechanism in male mice. Further work is required to establish potential sex-dependent effects and the relevance of this mechanism to appetite control in humans Cav3.1 has a broad expression distribution in the brain. While we showed that Cav3.1 expression in hypothalamic POMC neurons is a key player in mediating the anorectic response to dietary proteins, leucine sensing in other Cav3.1 expressing cells may also play a physiological role.

## Star Methods

Details of all reagents and animal models are detailed in the **Key Resource Table**.

### Resource availability

#### Lead contact

Further information and requests for reagents may be directed to and will be fulfilled by the Lead contact, Clemence Blouet (csb69@medschl.cam.ac.uk)

#### Materials availability

This study generated following unique reagents: 1.) various stable cell lines and plasmids of hCav3.1 mutants, and 2.) two new anti-mCacna1g gRNA AAV plasmids which are available upon request. All values of graphs and original western blot images can be found in the file Data S1.

### Experimental model and Subject details

#### Mouse strains and husbandry

All animal experiments (except for the mouse brain slice electrophysiology experiments performed at the University of Texas as noted below) were performed in accordance with the UK Home Office regulations under the Animals (Scientific Procedures) Act (1986) and with the approval of the University of Cambridge Animal Welfare and Ethics Review Board.

Wild-type C57BL/6J mice were obtained from Charles River UK (at 7 weeks of age) and were allowed to acclimatise to the facility for 1 week prior any experimental procedures. The following transgenic mouse strains were obtained from the Jackson Laboratory: P*omc-eGFP* (Tg(Pomc-EGFP)1Low/J; Strain #:009593) ^46^, *Pomc-Cre* (Tg(Pomc1-cre)16Lowl; Strain #:005965) ^47^, *R26-LSL-Cas9::EFGP* (tm1(CAG-cas9*,-EGFP)Fezh; Strain #:026175) ^48^. All transgenic mouse lines were kept on C57BL/6J genetic background. For all the experiments using Cre reporter lines, we conducted the work in hemizygous males or wild-type littermates randomly assigned to the experimental groups. For studies on wild-type mice, weight-matched groups were compared.

Mice were group-housed in individually ventilated cages with standard bedding and enrichment and maintained in a humidity-controlled room at 22–24°C on a 12-hr light/dark cycle with ad libitum access to water and standard laboratory chow diet (SAFE^®^ 105, SAFE^®^ Complete Care Competence, France) unless otherwise stated. Isocaloric modified diets with varying protein amounts were custom made by Research Diets as per the formulations in Table S6. 60% high fat diet was obtained from Research Diets (Cat# D12492). Before each dietary change, the mice were briefly exposed to the new diets to avoid neophobia or other novelty-related responses in subsequent experiments. Mice were acclimatised to the dosing procedures for at least 4 consecutive days prior any dosing studies.

For brain slice electrophysiology experiments performed at the University of Texas, male (6-to 18-week-old) pathogen-free mice were used for all experiments. All mice were housed under standard laboratory conditions (12 hr light-dark cycle) and a temperature-controlled environment with food and water available ad libitum. All experiments were performed in accordance with the guidelines established by the National Institute of Health Guide for the Care and Use of Laboratory Animals and approved by the University of Texas Institutional Animal Care and Use Committee. To identify POMC neurons, *Pomc-hrGFP* (Tg(Pomc1-hrGFP)1Lowl/J, Strain #:006421) mice were utilized ^48^. *Trpc5* KO (Trpc5tm1.1Clph) mice ^49^ were subsequently mated with *POMC-hrGFP* to identify POMC neuron on a *Trpc5* KO background.

#### Primary culture of post-weaning mediobasal hypothalamic neurons

Acute primary cultures of mediobasal hypothalamic neurons were prepared from 5 to 6-week-old POMC-EFGP male mice as previously described ^6^. Briefly, mice were fasted overnight and sacrificed with cervical dislocation. Brains were extracted into an ice-cold custom-formulated extraction media based on rat cerebrospinal fluid and microdialysis extracts ^5^. For each brain, 2 brain sections of 0.75 mm were cut using a McIlwain tissue chopper, starting 1.2 mm post the anterior commissure and ranging from −1.06 mm post Bregma to −2.54 mm post Bregma. The MBH was then dissected from coronal slices using a dissecting microscope. Tissue was transferred to papain (20 U/ml, Worthington, Lakewood, NJ, USA) pre-heated at 37°C and digested for 30 min at 37°C under agitation (Thermomixer, 500 rpm). After digestion, tissue extracts from 6 to 7 animals were pooled, transferred to a tube containing extraction media with 3.5 U/ml DNase I from bovine pancreas (Sigma) using a glass Pasteur pipette with a fire polished 1.5 mm opening and triturated with pipettes. The trituration supernatant was gently loaded on top of a BSA gradient (4% BSA prepared in extraction media (pH 7.4) loaded on top of 8% BSA (pH 7.4)), centrifuged for 5 min at 300 rcf, and the pellet was resuspended in a custom-formulated culture media (ref). 100 μl of resuspended cells were plated on the poly-D-lysine coated glass-bottom 35 mm dishes (MatTek Corporation) using a 0.5 mm trituration pipette, inside a cloning cylinder (8 mm^2^, Sigma). Plates were placed in an incubator (37°C, 5% CO2) for 1 hr. After that, an additional 2 ml culture media was added and the cloning cylinder removed. 4 to 6 culture dishes were prepared on each experimental day. Each culture dish was imaged once and represented our experimental unit.

#### Human derived hypothalamic neurons

Human induced pluripotent stem cells (FSPS13B line) were maintained in mTESR1 media and differentiated to hypothalamic neurons as previously described ^50^. Human hypothalamic neurons were allowed to differentiated for 30–50 days in vitro, at which point there were abundant neurons immunoreactive for POMC ^50,51^. To study cell-autonomous responsiveness to leucine, cultures were enzymatically dissociated with TrypLE and Papain as described previously (ref) and replated onto glass-bottomed 35 mm dishes (MatTek Corporation) coated with Geltrex (Thermo Fisher Scientific, 21041025). Cells were loaded with 5 μM Fura2 AM for 45 min and treated with vehicle or experimental solutions.

#### Brain slice preparation

Brain slices were prepared from young adult male mice (6-10 week-old) as previously described ^22,37,52^. Briefly, male mice were deeply anesthetized with i.p. injection of 7% chloral hydrate and transcardially perfused with a modified ice-cold ACSF (described below). The mice were then decapitated, and the entire brain was removed and immediately submerged in ice-cold, carbogen-saturated (95% O2 and 5% CO2) ACSF (126 mM NaCl, 2.8 mM KCl, 1.2 mM MgCl2, 2.5 mM CaCl2, 1.25 mM NaH2PO4, 26 mM NaHCO3, and 5 mM glucose). Coronal sections (250 mm) were cut with a Leica VT1000S Vibratome and then incubated in oxygenated ACSF (32°C-34°C) for at least 1 hr before recording. The slices were bathed in oxygenated ACSF (32°C-34°C) at a flow rate of ^~^2 ml/ min. The pH and osmolality of all ACSF with or without L-Leucine adjusted to within 7.2-7.3 and 290-300 mOsm/L range respectively. All electrophysiology recordings were performed at room temperature.

#### Cell culture

Human embryonic kidney HEK293 cell line (ATCC) and mouse embryonic hypothalamic mHypoE-N46 cell line (Cedarlane Labs, NC, USA) were maintained in DMEM with 2 mM stable glutamine supplemented with 10% fetal bovine serum (FBS) and 10,000 U penicillin/streptomycin at 37°C with 5% CO_2_. The cells were authenticated by STR profiling (Eurofins) and tested negative for mycoplasms by PCR (Merck) regularly. Cells were used for experiments before 30 passages. Lipofectamine 3000 (Thermo Fisher Scientific) were used for transient transfection of mHypoE-N46 cells according to manufacturer’s protocol (Publication No. MAN0009872 Rev C.0). TransIT-293 Transfection Reagent (Mirus Bio) was used for transfecting HEK293 cells according to manufacturer’s protocol (ML009-Rev.G 0117).

HEK293 cells stably expressing hCav3.1 (HEK293-hCav3.1) were generated monoclonally. In brief, 48hr after transient transfection with linearised hα1Ga-pDsRed plasmid (Addgene #45811) ^53^, cells were selected with antibiotic G418 at 750 μg/ml for about 2 weeks. Single clones of resistant cells were generated with serial dilutions and retrieved with the aid of cloning rings. This stable cell line was maintained with standard medium supplemented with 250 μg/ml G418 and was used for all cell experiments involving hCav3.1 except those involved truncated hCav3.1 and site-specific mutated hCav3.1.

HEK293 cells stably expressing hCav3.1 site-specific mutations and their wildtype control (WT) were generated polyclonally. 48 hr after transient transfection with the linearised hCav3.1 WT and mutant plasmids with puromycin-resistant genes, cells were selected with antibiotic puromycin at 2 μg/ml for about 2 weeks. The resultant stable cell lines were maintained with standard medium supplemented with 1 μg/ml puromycin and was used for cell experiments involving site-specific mutations of hCav3.1.

HEK293 cells stably expressing hTRPC5 (HEK293-hTRPC5) were generated polyclonally. 48 hr after transient transfection with the linearised hTRPC5 plasmid (Sino Biological #HG18741-UT) with a hygromycin-resistant gene, cells were selected with antibiotic hygromycin B at 500 μg/ml for about 2 weeks. The resultant stable cell lines were maintained with standard medium supplemented with 200 μg/ml hygromycin B.

### Method details

#### Stereotaxic surgical procedures and adeno-associated virus (AAV) vectors

Surgical procedures were conducted on 9- to 11-week-old mice under isoflurane anaesthesia. All the animals received Metacam prior to- and 24 hr post-surgery, and were allowed a 1-week recovery period during which they were acclimatized to injection procedures. For brain viral injections under anaesthesia, mice were stereotactically implanted with bilateral steel guide cannulas (Plastics One) positioned 1 mm above the ARH (A/P: 1.1 mm, D/V: 4.9 mm, and lateral: +/−0.4 mm from the bregma). Beveled stainless steel injectors (33 gauge) extending 1 mm from the tip of the guide were used for injections. For chronic cannula implantation, the cannula guide was secured in place with Loctite glue and dental cement (Fujicem2). Correct targeting was confirmed histologically post-mortem. The mice were allowed 1 week of recovery during which they were handled daily and acclimatized to the relevant experimental settings. For AAV injections, 500 nl AAVs per side were injected into the ARH with an injection rate of 100nl per minute and were kept in place for additional 5 minutes post-injection under anaesthesia. After that, the wound was closed by surgical suturing and allowed for 3 weeks recovery and transgene expression. The following AAVs produced by Vector Biolabs were used in this study: AAV/DJ-U6-anti-Cacna1g gRNA-CMV-SaCas9 (5.2 × 10^12^ vg/ml); AAV/DJ-CMV-SaCas9 (6.3 × 10^12^ vg/ml); AAV/DJ-U6-dual anti-Cacna1g gRNA-hSyn-mCherry (4.7 × 10^12^ vg/ml); AAV/DJ-hSyn-mCherry (7.1 × 10^12^ vg/ml).

#### MBH injection on behaving mice and food intake assessments

The same general design applied across all experiments involving MBH injection: studies were conducted in a home-cage environment; mice were food-deprived for 6hr during the day-time before receiving a bilateral parenchymal injection (100 nl/side, and 50 nl/min) of following drugs: L-leucine 2.1 mM (Sigma), TTA-P2 10 μM (Alomone), SAK3 0.1-1mM (Tocris), Leptin 5 μg/μl (R&D Systems), liraglutide 1 μg/μl (Tocris), lorcaserin 10 μg/μl (Generon) or control aCSF with matching DMSO content as a vehicle. Mice were immediately returned to their home cage for food intake analysis or culled for specific experiments as per indicated time intervals.

Mice were fasted from ZT5-11 for PhosphoTRAP assay and from ZT3-9 for RNAscope in situ hybridization and immunofluorescence histological assessments. They were culled with specific details in respective sections below.

For food intake studies, mice were fasted at ZT5, and the injection occurred 1 hr before dark onset. The mice were refed after the injection, and their food intake was monitored over various time points after refeeding. All the studies were conducted in a cross-over randomized manner on age- and weight-matched groups, and at least 4 days elapsed between each brain injection.

The chow mash diet was made by mixing the powder version of chow diet (SAFE^®^ 105, SAFE^®^ Complete Care Competence, France) with filter-sterilised deionised water in a 1:2 ratio. For the leucine-supplemented mash, 0.12 M L-leucine (Sigma) solution in sterile deionised water was used to mix with chow powder in 1:2 ratio, resulting in additional 31.5 mg leucine per 1 g chow powder consumption. In the food intake experiments involving mash, the mice were fast at ZT5, and at ZT10 mice were be moved to new cages with a polypropylene absorbent mat (New Pig). At ZT11, mice were presented with the mash diet in a ceramic pot at ZT11, with or without prior MBH injection of TTA-P2 as mentioned above. The evaporation of water content of the mash was controlled by putting the mash pots in an empty IVC cages, and after each measurement of mash consumption, the pots were refilled to the original level. For the preference testes involving mash, the position of control- and leucinemash containing pots were randomised, and the mice did not show positional bias in all the preference testes.

#### PhosphoTRAP assay

To identify enriched transcripts in mediobasal hypothalamic neurons activated or inhibited by the administration leucine compared to aCSF, PhosphoTRAP assay was performed with adaptions as previously described ^54,55^. 45 min after the MBH injection as described above, mice were culled by overdose of anaesthetic (300 mg/kg I.P. sodium pentobarbitone; Euthatal Solution for Injection, Dopharma Research B.V.). The MBH was micro-dissected in an ice-cold Buffer B (Hanks Balanced Salt Solution (HBSS) with 2.5 mM of HEPES at pH 7.4, 4 mM of NaHCO3, 35 mM of glucose, and 100 mg/mL of cycloheximide in methanol) under a 10x dissecting microscope. The hypothalami of 5 mice were pooled as a single sample resulting in 4 replicates per experimental condition (i.e. aCSF vs. Leu). Pooled hypothalami were manually homogenized in a 1ml of buffer C (10 mM of HEPES at pH 7.4, 150 mM of KCl, 5 mM of MgCl2, 100 nM of calyculin A, 2 mM of DTT, 100 U/mL of RNasin, 100 mg/mL of cycloheximide, and Roche protease and phosphatase inhibitor cocktails) and clarified by centrifugation at 2,000 g at 4°C for 10 min. The supernatants were transferred to a new tube. 70μl of 10% NP40 and 70μl of DHPC (1,2-diheptanoyl-sn-glycero-3-phosphocholine) were added, the samples were then mixed by inversion and allowed for 2 min incubation on ice, and centrifuged at 16,100 g at 4°C for 10 min. Supernatants were transferred to a new tube. 25 μl of each sample was collected and subject to RNA extraction as “Input sample”. The remaining sample was used for ribosome immunoprecipitation (IP).

Prior to the immunoprecipitation step, 100μl Protein A Dynabeads (Invitrogen) were washed 3 times with buffer A (10mM HEPES, 150mM KCl, 5mM MgCl2 and 1% NP40 (pH 7.4) before incubation with 4μg pS6 antibody against Ser240/244 (#2215, Cell Signalling Technology) and 0.1% bovine serum albumin (BSA) in buffer A (300 μl per IP sample) at 4°C overnight with continuous mixing on an end-over-end rotator. On the next day, the antibody-bead conjugates were washed twice with wash buffer D (10 mM of HEPES, 350 mM of KCl, 5 mM of MgCl2, 2 mM of DTT, 1% NP40, 100 U/mL of RNasin, 100 mg/mL of cycloheximide, and Roche protease and phosphatase inhibitor cocktails). After the last wash, the beads were resuspended in 200 μl of homogenisation buffer C supplemented with 50 μl of 10% NP40 and 10 μl of DHPC for 1 mL of buffer C and 1 μM of ZK10 (a gift from Dr. Zackary Knight, UCSF, US). The remaining sample supernatant was added to the antibody-beads conjugates, resuspended by pipetting and mixed with a rotator at 4°C for 10 min. The beads were washed 4 times with 0.9 mL of ice-cold wash buffer D and resuspended in 350 μl of RLT buffer and allowed for 5 min incubation on ice. The supernatant was then collected as “IP sample.”

RNA was extracted using the RNeasy Micro kit (#74004; Qiagen). RNA quantity was assessed using the Quant-iT RiboGreen RNA Assay kit (#R11490; Thermo Fisher Scientific), and the samples’ RNA quality was assessed using Pico chips on an Agilent Bioanalyser. The samples were cleared from DNA contamination using a TURBO DNA-free kit (#AM1907; Thermo Fisher Scientific).

cDNA was prepared using a Smart Seq v4 Ultra Low Input RNA kit (#634889; Clontech). Library preparation was done using a Nextera XT kit (Illumina), and the samples were run on a NexSeq 500 System (#FC-131-1024; Illumina).

#### PhosphoTRAP pathway analysis

Ingenuity Pathway Analysis (QIAGEN) was used to identify pathways that are up- or downregulated in each cluster between experimental conditions. Log2FC values, p values, and FDRs for each gene for each cluster were uploaded to the software.

#### Metabolic phenotyping

Body composition was analysed using a EchoMRI Whole Body Composition Analyser (EchoMRI, Houston, Texas). Promethion High-Definition Multiplexed Respirometry Cages (Sable Systems International, Las Vegas, Nevada) were used to analyse energy expenditure by indirect calorimetry, food intake, water intake, respiratory quotient and locomotor activity over 48 hr in mice single-housed for at least one week prior. Data collected during the first 24 hr of each run was discarded to allow for acclimatisation of mice to the altered cage environment.

#### Glucose phenotyping

For the oral glucose tolerance test, mice were food deprived for 6 hr from ZT1-7 and blood was sampled from the tail vein immediately prior to glucose bolus (gavage, 2 mg/kg), and 10, 20, 30, 60, 90, and 120 min following bolus administration. For the insulin sensitivity test, mice were food deprived for 6 hr from ZT1-7 and blood was sampled from the tail vein immediately prior to the administration of an insulin bolus (i.p., 0.75 U/kg), and 10, 20, 30, 60, 90, and 120 min following bolus administration. Blood glucose was analysed using an AlphaTrak3 handheld glucometer (Precision Xtra; MediSense).

#### Intranasal administration of substances on awake mice

Intranasal (IN) administration utilising a modified scuffing method to immobilise awake mice during drug administration via the nostrils was performed according to an established protocol ^56^. Briefly, mice were acclimatised for the modified scuffing method and mock injection (air-puffing only from pipette) either daily for two weeks in the chow-fed pilot experiment or bi-weekly over 12 weeks in the high fat diet experiment. On the day of actual experiment, a total of 24 μl of solution was applied to both nostrils of a mouse, over 4 sessions of 6 μl administration, ejecting slowly from a gel-loading pipette tip to allow formation of small droplets and inhalation by the mouse.

For pilot experiments, the following drugs were IN administered 1 hr before dark onset: 1 mM SAK3, 1 μg/ μl liraglutide or vehicle control (1% DMSO in saline) content. Food and mice were weighed before and 24 hr after the IN administration. Each of the testing conditions was separated by 4 days apart.

For the experiment on HF-fed mice, 7 week-old WT mice were transited to and maintained on 60% high fat diet for 12 weeks; upon then, only mice weighing over 40 g were used. Mice first received daily IN administration of vehicle (1% DMSO in saline) and subcutaneous (SC) injections of saline (5 ml/kg) during ZT 10-11 for 2 weeks to obtain an individual baseline response to the procedures and then daily I.N. administration of saline. After that, mice were proceeded to receive I.N. administration of 1mM SAK3 (or vehicle) with SC injection of 0.2 mg/kg liraglutide (or vehicle) during the same time-of-day for another 2 weeks. Food and animals were weighed daily throughout the study.

#### Brain tissue preparation for histological assessments

Mice were anaesthetised with an intraperitoneal injection of 100 mg/kg sodium pentobarbitone (Dolethal, Vetoquinol UK Ltd) then transcardially perfused with chilled 0.1 M heparinized phosphate buffered saline (PBS) followed by 4% paraformaldehyde (pH 7.4). The brains dissected and post-fixed overnight at 4°C in 4% PFA then cryoprotected in 30% (w/v) sucrose solution in PBS with 0.03% sodium azide as a preservative for at least 48 hr prior to processing. For RNA-scope experiments, the cryoprotected brains were embedded in Optimal Cutting Temperature (OCT) medium (CellPath, Newtown, UK) in plastic molds, frozen on crushed dry ice and then stored at -80°C until further processing.

#### Brain tissues preparation for simultaneous gDNA, RNA and protein isolation

To validate the CRISPR mediated MBH Cacna1g KO models, the micro-dissected mediobasal hypothalami of control and Cacna1g^MBH KO^ mice were collected in the RNAlater solution (Thermo Fisher Scientific) and stored according to the manufacturer’s protocol (7020M Rev. G 15Jul2014). On the day of processing, the tissues were removed from RNAlater solution and then homologized with a hand-held electric homogeniser and a pistil (Sigma Aldrich) in 300 μl TRIzol Reagent (Thermo Fisher Scientific) to simultaneously isolate of gDNA, total RNA and protein according to the manufacturer’s protocols (MAN0001271 Rev. B.0 and MAN0016385 Rev. A.0).

#### Multiplexed Fluorescent in situ hybridization (FISH) with RNAscope

Simultaneous detection of mouse *cFos, Trpc5, Cacna1g and Pomc* was performed on frozen sections using Advanced Cell Diagnostics (ACD) RNAscope 2.5 LS Multiplex Reagent Kit (Cat No. 322800), RNAscope 2.5 LS Probe Mm-Fos (Cat No. 316928), RNAscope 2.5 LS Probe Mm-Trpc5 (Cat No. 476248), RNAscope^®^ 2.5 LS Probe Mm-Cacna1g-C2 (Cat No. 459768 C2), RNAscope^®^ 2.5 LS Probe Mm-POMC-C3 (Cat No. 314088-C3), (ACD, Hayward, CA, USA). Briefly, OCT embedded frozen brains were sliced at 12 µM using a Leica CM1950 cryostat directly onto Superfrost Plus slides (Thermo Fisher Scientific) in an RNase-free environment and stored at −80°C until use. Upon processing, slides were thawed at room temperature for 10 min and post-fixed in pre-chilled 4% PFA for 15 min at 4oC, washed in 3 changes of PBS for 5 min each before dehydration through 50%, 70& 100% Ethanol for 5 min each. The slides were air-dried for 5 min before loading onto a Bond Rx instrument (Leica Biosystems). Slides were prepared using the frozen slide delay prior to pre-treatments using Epitope Retrieval Solution 2 (Cat No. AR9640, Leica Biosystems) at 95°C for 2 min, and ACD Enzyme from the Multiplex Reagent kit at 40°C for 15 min. Probe hybridisation and signal amplification was performed according to manufacturer’s instructions.

Opal 650 (Akoya Biosciences Cat No. FP1496A) detection at 1:750 dilution of Trpc5 or Fos, Opal 570 (Akoya Biosciences Cat No. OPI-001003) detection at 1:750 of Cacna1g, and Opal 690 (Akoya Biosciences Cat No. OPI-001006) detection at 1:3000 dilution of POMC were performed on the Bond Rx according to the ACD protocol. Slides were then removed from the Bond Rx and mounted using Prolong Diamond (Thermo Fisher Scientific).

The brain sections were imaged using a spinning disk Operetta CLS (PerkinElmer) in confocal mode using an sCMOS camera and a 40x automated water-dispensing objective. The sections were imaged with z stacks at intervals of 1 μm. ROIs included the ARH and VMH. Gain and laser power settings remained the same between the experimental and control conditions during each experiment. Harmony software (PerkinElmer) was used to automatically quantify the number of labelled RNA molecules (spots) per cell and the number of labelled cells among other metrics.

#### Immunohistochemistry and imaging

Perfused brains were post-fixed overnight at 4°C in 4% PFA then cryoprotected in 30% (w/v) sucrose (Fisher Scientific) solution in PBS for at least 48 hr prior to processing. Tissues were covered with Optimal Cutting Temperature (OCT) medium (CellPath, Newtown, UK) and sections were obtained at 25 μm on a Leica SM2010R Freezing Microtome (Leica, Wetzlar, Germany). All sections were subjected to heat-mediated antigen retrieval in 10 mM sodium citrate (pH6.5; Fisher Scientific) in distilled water for 20 min at 80°C prior to washing 3 times in PBS. For all experiments, sections were blocked in normal goat serum (NGS, Abcam) diluted in PBS containing 0.3% Triton X-100 (0.3% PBST; Sigma) for 1 hr prior to primary antibody incubation overnight at 4°C. Following primary antibody incubation, sections were washed 3 time with 0.1% PBST and incubated with appropriate fluorophore-conjugated secondary antibodies diluted 1:500 in 0.3% PBST for 2 hr at room temperature. Sections were subsequently washed with 0.1% PBST and mounted to Clarity microscope slides (Dixon Science, Edenbridge, UK) under coverslips (1.0 thickness; Marienfeld, Lauda-Königshofen, Germany) with Vectashield Vibrance Mounting Medium with 4′,6-diamidino-2-phenylindole (DAPI; Vector Laboratories, Newark, California). The following primary antibodies were used: anti-Phospho-S6 Ribosomal Protein (Ser240/244) (D68F8) Rabbit monoclonal-Alexa Fluor 594 Conjugate) (1:200; Cell Signalling Technology, Cat# 9468), anti-cFOS rabbit monoclonal and guinea pig monoclonal (1:1000; Synaptic Systems, Cat# 226008 and 226308, respectively), anti-Cav3.1 (C-terminal) rabbit polyclonal (1:200; Abcam, Cat# ab203577), anti-GFP chicken polyclonal (1:1000; Abcam, Cat# ab13970), anti-DsRed mouse monoclonal (1:1000, Clontech, Cat# 632392), anti-POMC rabbit polyclonal (1:1000; Phoenix Pharmaceutical, Cat# H-029-30).

The sections were imaged using a Zeiss Axio slide scanner with a 20x objective or a Leica SP8 confocal microscope with 40x or 63x objectives. The imaging settings remained the same between the experimental and control conditions. Images of tissue sections were digitized, and areas of interest were outlined based on cellular morphology and using Paxinos and Franklin’s brain atlas [31]. The images were analyzed using the ImageJ manual cell counter or Zeiss ZEN 2.3 software.

#### Quantitative real-time polymerase chain reaction (qPCR)

Total RNA of tissues and cell cultures was extracted using either TRIzol reagent according to the manufacturer’s protocol (MAN0001271 Rev. B.0). cDNA synthesis of 1 μg RNA was performed using the High-Capacity cDNA Reverse Transcription Kit with random hexamer primers (Thermo Fisher Scientific). qPCR was performed using either TaqMan Universal PCR Master Mix for reaction based on dual-labelled probes or the SYBR Green PCR Master Mix for SYBR Green reactions (both from Thermo Fisher Scientific) on a QuantStudio 5 Real-Time PCR System (Thermo Fisher Scientific). The average of technical duplicates of a biological sample was used for quantification. Relative gene expression was quantified using the ΔΔ threshold cycle (Ct) method with adjustments to the amplification efficiencies of individual primer pairs. *Eef1α* was used as the reference gene for all experiments. The specificity of all SYBR Green primers were validated with melting curve analyses. All custom-designed standard qPCR primers and 6-FAM-TAMRA dual labelled probes were designed to target the exon-exon boundary of a gene or separated by a long intron to exclude amplifying genomic DNA, and were synthesised by Merck. Predesigned dual-labelled TaqMan Gene Expression Assay probes were purchased from Thermo Fisher Scientific. Information for oligos used are shown in the **Key Resources Table**.

#### SDS-polyacrylamide gel electrophoresis (PAGE) and Western blot

Protein from MBH tissues were extracted with TRIzol Reagent as described above. Protein from cultured cells were lysed with RIPA lysis buffer (1% triton X-100, 2% sodium dodecylsulfate (SDS), 1% sodium deoxycholate, 1 % NP40 and 1x cOmplete protease inhibitor cocktail (Roche, Grenzach-Wyhlen, DE) in Tris-buffered saline (TBS) and subjected to sonication using a Branson 450 sonifier (Thermo Scientific; amplitude: 50, duty: 30%, duration: 30 s). In the case of detecting phosphorylated proteins, 1x Roche pSTOP phosphatase inhibitors was included to the lysis buffer. Protein concentrations were determined by BCA protein assay kit (Thermo Scientific). Appropriate protein amount was further diluted with SDS sample-loading buffer (50mM Tris-HCl pH 6.8, 0.05% (w/v) bromophenol blue, 6.7% glycerol, 1% SDS, 1.5% β-mercaptoethanol).

Due to the large molecular mass of Cav3.1 (^~^262 kDa), a modified immunoblotting protocol was used for detecting Cav3.1. For detecting Cav3.1, samples were heated at 70°C for 10 min. 60 μg protein from MBH tissues or 40 μg from cultured cells were loaded per sample onto a 4 – 12% pre-casted Novex Tris-Glycine Mini Protein Gels (Thermo Fisher Scientific). After electrophoresis, gel was first equilibrated in a 2x Tris-Glycine Transfer buffer (25 mM Tris, 192 mM glycine, pH 8.3) containing 0.04% SDS for 10 min. After that, Western blotting was performed on a methanol-activated PVDF membrane using 1x Tris-Glycine Transfer buffer containing 10% methanol and 0.01% SDS at 12 V overnight or 20 V for 3h. Both electrophoresis and Western blotting were carried out with the Invitrogen Mini Blot system (Thermo Fisher Scientific).

For detecting other proteins, samples were heated at 95°C for 5 min. 40 μg protein from cell culture were loaded per sample. A standard SDS-PAGE and Western blotting protocol was used as described above except the following differences: 1.) standard 10% PAGE gel was used; 2.) the pre-equilibration step was omitted; 3.) the Transfer buffer contained 20% methanol and no SDS; 4.) The transfer was carried out at 20V for 2 hr.

5% non-fat milk (Bio-Rad) was used as the blocking agent for primary antibodies detecting non-phosphorylated proteins and all horseradish peroxidase (HRP)-conjugated secondary antibodies (Abcam, Cat# ab205718-ab205719, respectively). 5% BSA was used as the blocking agent for primary antibodies detecting phosphorylated proteins. SuperSignal West Pico PLUS Chemiluminescent Substrate (Thermo Fisher Scientific) was used for developing the chemiluminescence signal of the blot, which was then imaged and digitalised with the ChemiDoc Imaging System (Bio-Rad). Densitometric analysis of band intensity was performed with Quantity One software (Bio-Rad). Quantification across multiple blots were normalised with a shared calibrating samples with the same amount of protein. Endogenous GAPDH on the same blot was used as the internal loading control for standard Western blot experiments. Total non-phosphorylated form of the target proteins from the same blot was used as the internal loading control for phosphorylated protein after stripping with the Restore PLUS Western Blot Stripping Buffer (Thermo Fisher Scientific).

The following primary antibodies were used: anti-Cav3.1 (C-terminal) mouse monoclonal (1:1000; abcam, Cat# ab134269), anti-TRPC5 rabbit polyclonal (1:1000; Alomone Labs, Cat# ACC-020), anti-GAPDH mouse monoclonal (1:1000; Santa Cruz Biotechnology, Cat# sc-32233), anti-phospho-p70 S6 Kinase (Thr389) rabbit polyclonal (1:1000; Cell Signalling Technology, Cat# 9205), anti-P70 S6K rabbit monoclonal (1:1000; Cell Signalling Technology, Cat# 2708) and anti-HA mouse monoclonal (1:1000; Cell Signalling Technology, Cat# 2367).

#### Blood amino acid profiling

Whole blood was collected terminally into a EDTA-treated tube (Sarstedt) using right ventricle cardiac puncture from mice under anaesthesia following an intraperitoneal injection of 100 mg/kg sodium pentobarbitone. Plasma was retrieved as supernatant after centrifuging the whole blood at 4°C at 2000 g for 15 mins. 5 µl of plasma sample was added with mixed isotope-labelled amino acid internal standards, then extracted by protein precipitation using formic acid and isopropyl alcohol followed by derivatisation using AccQ-Tag Ultra Derivatising Kit (Water 186003836). Derivatized amino acid standards in 0.1% formic acid was prepared and analysed along with the extracted samples. Liquid chromatography and tandem mass spectrometry was performed on the Shimadzu Nexera X2 Liquid chromatography system linked to Sciex 6600 Triple Quad mass spectrometer. 19 proteinogenic amino acids (except glycine due to protocol limitations) plus citrulline, orthenine and taurine were analysed.

#### Urine analyses

Urine was collected from mice during ZT8-10 after at least 3 weeks on indicated diets on two separated days without other procedures with gentle abdominal massaging to encourage urination on a sheet of water repulsive parafilm. Urinary albumin was analysed with a mouse albumin ELIZA kit (Cat# E99-134, Bethyl Laboratories, US); creatinine was measured with the Creatinine Assay Kit (Cat# EXO-1012, Ethos Biosciences, US); urea was measured with urea assay kit (Cat# ab234052, Abcam) and glucose was measured with glucose assay (Cat# ab65333, Abcam). Urinary osmolarity was measured by the Osmomat 3000 osmometer (Gontec, DE).

#### SAK3 LC-MS/MS analysis

Tissues were weighed and transferred into vials containing Lysing Matrix D beads (MP Bio) along with 500 µl of 80% ACN in water. Samples were homogenised on a VelociRuptor (SLS) homogenisation system at 4.5 m/s for 1 min and were cooled on ice between each of the three cycles. The heart tissues required a second homogenisation step with the addition of a metal ball to break apart the tough muscle tissue. Samples were centrifuged for 10 minutes at 12000 g and 250 µl of the supernatants was transferred to an Eppendorf LoBind plate. The supernatants were evaporated under oxygen free nitrogen at 40 ºC and reconstituted into 100 µl of 10% ACN in water and 50 µl of internal standard (D6 memantine) was added. A calibration line of SAK3 in 0.1% formic acid was prepared and analysed along with the extracted samples. Liquid chromatography and tandem mass spectrometry was performed on a Waters M-class Acquity linked to a TQ-XS mass spectrometer. A selected reaction monitoring (SRM) method was used targeting SAK3 (369.99/323.92) and labelled Memantine (180.2/163.04). The data was processed on TargetLynx XS (v 4.2, Waters) and SAK3 content was normalized by tissue weight.

#### Calcium imaging

Cells were loaded with 5 μM Fura2 AM dye (Life Technologies) for 30 min, washed with custom-formulated extraction media based on rat cerebrospinal fluid ^6^, and imaged using an inverted fluorescence microscope (Olympus IX71, Olympus, Southend on Sea, UK) with a 40× oil-immersion objective lens. GFP (to identify POMC cells) was excited at 488 nm and fura-2 at 340 nm and 380 nm using a monochromator (Cairn Research, Faversham, UK) and a 75 W xenon arc lamp, and emissions were recorded using an Orca ER camera (Hamamatsu, Welwyn Garden City, UK), a dichroic mirror and a 510 nm long pass filter. All images were collected on MetaFluor software (Molecular Devices, Wokingham, UK). The ratio of fura-2 emissions at 340 and 380 nm (340/380 ratio) was used to monitor changes in the intracellular Ca^2+^ concentration. Solutions were perfused continuously at a rate of approximately 0.5 ml/min.

#### Whole cell patch-clamp electrophysiological recording on brain slices

The pipette solution for whole-cell recording was modified to include an intracellular dye (Alexa Fluor 350 hydrazide dye) for whole-cell recording: 120 mM K-gluconate, 10 mM KCl, 10 mM HEPES, 5 mM EGTA, 1 mM CaCl2, 1 mM MgCl2, and 2 mM MgATP, 0.03 mM Alexa Fluor 350 hydrazide dye (pH 7.3). Epifluorescence was briefly used to target fluorescent cells, at which time the light source was switched to infrared differential interference contrast imaging to obtain the whole-cell recording (Zeiss Axioskop FS2 Plus equipped with a fixed stage and a QuantEM:512SC electron-multiplying charge-coupled device camera). Electrophysiological signals were recorded using an Axopatch 700B amplifier (Molecular Devices); low-pass filtered at 2-5 kHz, and analysed offline on a PC with pCLAMP programs (Molecular Devices). Membrane potentials and firing rates were measured from POMC neurons in brain slices. Recording electrodes had resistances of 2.5-5 MΩ when filled with the K-gluconate internal solution.

A change in membrane potential was required to be at least 2 mV in amplitude in response to drug application. Membrane potential values were not compensated to account for junction potential (−8 mV). Effects of L-Leucine on AP frequency (over 0.5 Hz) before and during acute L-Leucine bath application were analysed within a recording using the Kolmogorov-Smirnov (K-S) test (a nonparametric, distribution-free goodness-of-fit test for probability distributions).

#### Whole cell patch-clamp electrophysiological recording on HEK293 cell

HEK293 cells were transfected with pCAG-hCav3.1-ires-EGFP plasmid using TransIT-293 Transfection Reagent 48-72 hours before experiments. Transfected cells were plated in 35mm dishes the day before the experiments. Recordings of single EGFP+ cells were performed at room temperature (20-24°C). Regular bath solution or bath solution containing 1 mM L-Leucine (prepared fresh on day of experiment) were applied directly onto cells using a custom-made gravity fed perfusion system. A constant flow of bath solution was applied for baseline recordings. The internal pipette solution contained (in mM): 115 CsCl, 1 MgCl2, 0.9 CaCl2, 20 HEPES, 5 Mg-ATP, 11 BAPTA, Ph 7.3 with CsOH. Bath solution contained (in mM): 10 CaCl2, 140 TEA-Cl, 10 HEPES, 10 D-glucose, 2.5 CsCl and 1 MgCl2 Ph 7.3 WITH TEAOH. Cells were recorded in conventional whole cell configuration after obtaining a gigaΩ seal and recorded using an Axopatch 200B amplifier connected through a Digidata 1440A A/D converter and pclamp software (Molecular Devices). Microelectrodes were pulled from borosilicate glass (1.5 mm OD, 1.17 mm ID; Harvard apparatus) using a PC-100 Narishige microelectrode puller (Narishige, Japan) and the tips coated with refined yellow beeswax. Electrodes were fire polished using an MF-830 micro forge (Narishige, Japan) and had resistances of 2-3 MΩ when filled with internal pipette solution. A silver/AgCl ground wire connected to the bath solution via a 3M KCl agar bridge was used as a ground. To obtain current-voltage relationships cells were challenged to a 2-step activation/inactivation protocol, with an initial holding potential of -100 mV and stepped from -90 to -10 mv, in 10 mv steps (500 ms first duration, 2 seconds step to step). I/V plots, inactivation constants and raw traces were obtained and analysed using Clampfit software (Molecular Devices).

#### Plasmid construction

The guide RNAs used were designed to target an early exon common to all transcript variants of mouse *Cacna1g* with the aid of the CRISPick gRNA design online tool (Broad Institute). The selected custom gRNAs in pX601-AAV-CMV::NLS-SaCas9-NLS-3xHA-bGHpA vector (for Cacna1g^MBH KO^) ^57^ and pSpCas9(BB)-2A-GFP PX458 vector (for Cacna1g^POMC KO^) ^58^ were purchased directly from Genescript (company) and validated on mHypoE-N46 cells for CRISPR-mediated indel mutations.

The validated gRNA for Cacna1g^MBH KO^ was cloned to AAV-U6-gRNA-CMV-SaCas9 vector by Vector Biolabs. The two validated gRNAs along with their U6 promoters for Cacna1g^POMC KO^ were cloned to pAAV-Linker-hSyn::mCherry.3xFLAG-WPRE (ref. Addgene #120391) in tandem with MluI and SacI sites respectively. The resultant plasmids along the gRNA-less empty vectors (as controls) were then provided to Vector Biolabs for AAV production.

The hCav3.1 from hα1Ga-pDsRed plasmid (ref; Addgene Cat# 45811) were subcloned to following vectors: 1.) pCAG-ires-GFP vector (Addgene #45025) with EcoRI and NotI sites for cell electrophysiology experiments; 2.) piRES-puro vector (Addgene Cat# 25728) with EcoRI and NotI sites for site-directed mutagenesis experiments.

The original hα1Ga-pDsRed plasmid was modified to include an HA epitope tag and a stop codon to the C-termianl using NotI and XbaI sites for the domain mapping experiment. The full length hCav3.1 were then re-cloned to this new vector without the DsRed sequence and in-frame with the HA tag using the EcoRI and NotI sites. The truncated hCav3.1 fragments (Domain I fragment: 1 – 735 aa; Domain II fragment: 736 – 1267 aa; Domain III fragment: 1268 – 1586 aa; Domain IV fragment: 1587 – 2314 aa) were cloned to this vector using FseI and NotI sites retaining the 32 N-terminal amino acid residues of full-length hCav3.1 which contains the secretion signal peptide and is also recognised by the anti-Cav3.1 antibody for immunoprecipitation in the fluorescent leucine binding assay.

Leucine binding hCav3.1 mutants were generated by performing site-directed mutagenesis using standard overlapping PCR method on the hα1Ga-pDsRed plasmid. The resultant hCav3.1 mutants were cloned into the piRES-puro vector using EcoRI and NotI sites. The sequence integrity of all plasmids was confirmed by Sanger sequencing (Eurofins Genomics).

#### Indel mutation detection assay on genomic DNA

Genomic DNA was extracted from MBH tissues using TRIzol as described above and from cultured cells with QIAamp DNA kit (Qiagen). The T7 endonuclease I based mutation based EnGen Mutation Detection Kit (New England Biolabs) was used to detect on-target CRISPR-mediate indel mutations according to the manufacturer’s protocol (Version 3.0_7/20). The top exonic off-target loci were predicted using the CCTop online tool ^59^.

#### Live cell fluo8 Ca^2+^ flux assay

2.5×10^4^ cells per well were plated to poly-D-lysine coated 96 well plates with black wall and clear-bottom for 72hr and allowed to grow to confluence. After that, cells were rinsed once with imaging medium (CaCl_2_ 1.8 mM, MgCl_2_ 0.8 mM, KCl 5.4 mM, NaCl_2_ 115 mM, glucose 13.8 mM) and loaded with a cell permeable fluorescent Ca^2+^ sensitive dye Fluo8-AM (Abcam) at 5 μM and allowed for 30 min incubation at 37°C. Imaging medium was then replaced (100 μl per well) and allowed for incubation at room temperature (~24°C) in darkness for 30 min. Cells were then treated with indicated substances in a 5 μl volume. The plate was then sealed with a MicroAmp optical adhesive film (Thermo Fisher Scientific) and put into the Tecan infinite M1000Pro plate reader. After a short 5 second orbital shaking, fluorescence kinetics were measured under the bottle-reading mode with following settings: excitation: 490±9 nm, emission 520±9nm, number of Flashes: 10, flash frequency: 400 Hz, integration time: 20 us, interval time: 5 s, kinetic cycle: 150.

#### In vitro fluorescent leucine and valine binding assay

An immunoprecipitation-based fluorescent leucine/valine binding assay was adapted from a radioactive labelled version ^17^. The HEK293-hCav3.1 stable cells were plated to a 6-well cell culture plate at 1 × 10^6^ per well density and allowed to grow to confluence for 48 hours. For the domain mapping experiment, HEK293 cells were transiently transfected with full-length and truncated hCav3.1 and allowed for expression for 48 hr as mentioned above. On the day of performing the binding assay, the antibody was conjugated to the magnetic protein A Dynabeads before cell harvesting. In brief, 33.3 μl beads per sample were washed with 1 mL TBS with 1% Triton X-100 (TBST) then blocked with 500 μl TBST with 1μg/ml BSA for 20 min at 4°C with rotation, followed by incubating with 1 μg N-terminal anti-Cav3.1 (Alomone Labs, Cat# ACC-021) (or 1 μg rabbit IgG as a control) in 500 μl lysis buffer with 1 μg/ml BSA at 4°C with rotation for 1 hr. After that, beads were washed twice with TBST.

Cells were harvested using a lysis buffer (TBS with 1% Triton X-100, 1x Roche EDTA free cOmplete protease inhibitor, 1x Roche pSTOP phosphatase inhibitors, 50mM sodium fluoride, 1mM sodium orthovanadate) and allowed to mix with gentle rotation at 4°C for 1 hr. After that, cells were centrifuged with 16100 rcf at 4°C for 10 min. Supernatant was then loaded to washed beads and allowed to incubate at 4°C with gentle rotation for 2 hr. Heat-denatured samples were obtained by heating the supernatant to 70°C for 10 min followed by chilling on ice for 5 min prior loading to beads. Then, the beads were washed 1 time with lysis buffer followed by 3 times high-salt TBST adjusted to 500 mM sodium chloride.

After the washing, beads were resuspended with 500 μl assay buffer (TBS with 0.1% Triton X-100) with 10 μM FAM-leucine or FAM-valine (Cambridge Research Biochemicals) and allowed to incubated at 4°C with gentle rotation for 1 hr. 10 mM unlabelled leucine/ valine were added to respective control samples at this stage to validate the specificity of the binding assay. The beads were then washed 3 times with 1 mL assay buffer. The immunocomplex was eluted from beads with 135 μl elution buffer (PBS with 1% SDS) and heating at 70°C for 10 min. The supernatant was then retrieved and 3 technical replicates per condition with 40 μl each were loaded to a 96 well plates with black wall and clear-bottom for fluorescence measurement the Tecan infinite M1000Pro plate reader (bottle-reading mode, excitation: 490±9 nm, emission 520±9 nm, number of Flashes: 10, flash frequency: 400 Hz, integration time: 20 μs). After the fluorescence measurement, the samples were retrieved and added 1X SDS sample-loading buffer for Western Blot analysis as described below.

#### Co-immunoprecipitation

HEK293-hCav3.1 cells were transiently transfected with hTRPC5 plasmid (Sino Biological; Cat# HG18741-UT) using TransIT-293 Transfection Reagent. After 48 hr, cells harvested with an IP buffer (1% Triton X-100, 10% glycerol, 1x Roche cOmplete protease inhibitor cocktail with EDTA, in TBS pH7.4) at 4°C for 1 hr with gentle rotation and cleared by centrifugation. The cell lysates were subjected to anti-hCav3.1 IP by incubating with 1 μg of anti-hCav3.1 Rabbit antibody (Alomone) (or with 1 μg of unimmunised rabbit IgG (Thermo Fisher) as control) together with 30 μl of protein A agarose (Thermo Fisher) for 2 hr at 4°C with rotation. The immuno-complexes were washed 5 times with IP buffer and eluted by 1x SDS sample buffer and subjected to Western blot analysis as described above.

#### In silico prediction of L-leucine binding sites on Cav3.1

To predict the possible binding site of L-Leucine onto Cav3.1, a blind docking approach using AutoDock Vina 1.1.2 was used as described previously ^60^. The cryo-EM derived structure of human Cav3.1 protein (PDB id: 6KZO) ^18^ as well as the 3D structures of the ligands (L-Leucine and L-Valine) were obtained from the Protein Data Bank. Prior to docking, the protein structure was prepared using the Dock Prep module of UCSF Chimera version 1.17.3 (https://www.cgl.ucsf.edu/chimera/). Initially, we performed blind docking of L-Leucine (the active ligand) and with L-Valine as a decoy against the entire Cav3.1 structure using AutoDock Vina at an exhaustiveness of 24 in three independent runs. Afterwards, total 27 docked poses (3 × 9/docking run) for each ligand were saved and clustered which allowed segregation into distinct potential sites or pockets. Through visual inspection, we then disregarded the sites where both Leucine and Valine poses overlapped. The sites that appeared to be potentially unique for L-Leucine recognition were then ranked based on the average of predicted energy of interaction (DG, kcol/mol) of the docked L-Leucine poses clustered per site. Finally, a focused docking of L-Leu was performed for each site using GOLD suite version 5.3 (CCDC, Cambridge) done in 15 independent runs/site. The most reproducible pose per site was then considered as the final, plausible binding mode of L-Leucine at each site. These poses in complex with the Cav3.1 protein were then subjected to 2D ligand interaction analysis through PLIP server ^61^ and depicted using MOE version 2019 (Chemical Computing Group, Montreal, Canada).

#### Quantification and Statistical analysis

Unless otherwise stated, all the data were presented as means ± SEM and were analysed using GraphPad Prism 10 software package. For all the statistical tests, an α risk of 5% was used to define statistical significance. For pairwise comparisons were conducted using two-tailed t tests assuming equal variance between groups. One-factor multiple comparisons (across all groups or against control group) were tested with one-way ANOVAs followed by Holm-Sidak post hoc tests. For repeated measurement of the same subjects, paired t test or repeated measures one-way ANOVA were used. Two-factors multiple comparisons were tested with two-way ANOVAs followed by Holm-Sidak post hoc tests. All kinetics were analysed using repeated-measures two-way ANOVAs and adjusted with Holm-Sidak post hoc tests. Dietary and aCSF/leucine treatments were allocated randomly in weight-matched groups. We used blinding (to mouse genotype, viral treatment, or drug delivered) for in vivo experiments and to conduct image analyses. Additional statistical details on each experiment can be found in the Figure or figure legends.

### Key Resource Table

**Table:** 

Reagents or Resources	Source	Identifier
**Mouse models (stock strains)**		
C57BL/6J-Tg(Pomc-EGFP)1Low/J	Jackson Laboratory	Strain# 009593; RRID: IMSR_JAX:009593
STOCK Tg(Pomc1-cre)16Lowl/J	Jackson Laboratory	Strain# 005965; RRID: IMSR_JAX:005965
B6J.129(B6N)- Gt(ROSA)26Sortm1(CAG-cas9*,- EGFP)Fezh/J	Jackson Laboratory	Strain# 026175; RRID: IMSR_JAX:026175
FVB-Tg(Pomc1-hrGFP)1Lowl/J	Jackson Laboratory	Strain# 006421; RRID: IMSR_JAX:006421
129S1/SvImJ-Trpc5tm1.1Clph/J	Jackson Laboratory	Strain# 030804; RRID: IMSR_JAX:030804
B6(129S4)-Cacna1gtm1Stl/J	Jackson Laboratory	Strain# 021932; RRID: IMSR_JAX:021932
**AAVs**		
AAV/DJ-U6-anti-Cacna1g gRNA-CMV-	This study; AAV produced by	NA
SaCas9	Vector Biolabs	
AAV/DJ-U6-dual anti-Cacna1g gRNA-	This study; AAV produced by	NA
hSyn-mCherry	Vector Biolabs	
AAV/DJ-CMV-SaCas9	Vector Biolabs	Cat# 7121
AAV/DJ-hSyn-mCherry	Vector Biolabs	Cat# VB4884
**Cell lines**		
hiPSCs FSPS13B	A gift from L. Vallier Lab (University of Cambridge)	NA
HEK293	ATCC	Cat# CRL-1573; RRID: CVCL_0045
mHypoE-N46	Cedarlane Labs	Cat# CLU138; RRID: CVCL_D459
**Drugs**		
L-Leucine	Merck	Cat# L8912
aCSF	Tocris	Cat# 3525
TTA-P2	Almone	Cat# T-155
L-Valine	Merck	Cat# V0513
L-Isoleucine	Merck	Cat# I7403
Torin 1	Tocris	Cat# 4247
SAK3	Tocris	Cat# 6239
Recombinant Mouse Leptin	R&D Systems	Cat# 498-OB
Lorcaserin.HCl	Generon	Cat# M2821
Liraglutide	Tocris	Cat# 6517
**RNAscope FISH probes**		
RNAscope 2.5 LS Probe Mm-Fos	Advanced Cell Diagnostics	Cat# 316928
RNAscope 2.5 LS Probe Mm-Trpc5	Advanced Cell Diagnostics	Cat# 476248
RNAscope 2.5 LS Probe Mm-Cacna1g-C2	Advanced Cell Diagnostics	Cat# 459768-C2
RNAscope 2.5 LS Probe Mm-POMC-C3	Advanced Cell Diagnostics	Cat# 314088-C3
**Primary antibodies**		
anti-Cav3.1 (C-terminal) mouse monoclonal	abcam	Cat# ab134269
anti-Cav3.1 (C-terminal) rabbit polyclonal	abcam	Cat# ab203577
anti-Cav3.1 (N-terminal) rabbit polyclonal	Alomone	Cat# ACC-021; RRID: AB_2039779
anti-cFos rabbit monolonal	Synaptic Systems	Cat# 226 008; RRID: AB_2891278
anti-cFos guinea Pig monolonal	Synaptic Systems	Cat# 226 308; RRID: AB_2905595
anti-Phospho-S6 Ribosomal Protein (Ser240/244) rabbit polyclonal	Cell Signaling Technology	Cat# 2215; RRID: AB_331682
anti-Phospho-S6 Ribosomal Protein (Ser240/244) (D68F8) rabbit monoclonal - Alexa Fluor 594 Conjugate	Cell Signaling Technology	Cat# 9468; RRID: AB_2716873
anti-GFP chicken polyclonal	abcam	Cat# ab13970; RRID: AB_300798
anti-DsRed mouse monoclonal	Takara Bio	Cat# 632392; RRID: AB_2801258
anti-POMC rabbit polyclonal	Phoenix Pharmaceutical	Cat# H-029-30; RRID: AB_2307442
anti-TRPC5 rabbit polyclonal	Almone	Cat# ACC-020; RRID: AB_2040241
anti-GAPDH mouse monoclonal	Santa Cruz	Cat# sc-32233; RRID:AB_627679
anti-phospho-p70 S6 Kinase (Thr389) rabbit polyclonal	Cell Signaling Technology	Cat# 9205; RRID: AB_330944
anti-P70 S6 Kinase rabbit monoclonal	Cell Signaling Technology	Cat# 2708; RRID: AB_390722
anti-HA mouse monoclonal	Cell Signaling Technology	Cat# 2367; RRID:AB_10691311
**Oligonucleotides for qPCR**		
Actb; FP: GCTCTGGCTCCTAGCACCAT; RP: GCCACCGATCCACACAGAGT; Probe: ATCAAGATCATTGCTCCTCCTGAGCGC	This study; synthesised by Merck	NA
Ano6; FP: AGGAGCGCATCCCATTTACC; RP: CGATCACAGAGGCGATGATGA; Probe: CTGTGTGCGAGCGCCGTCTTCTTCTG	This study; synthesised by Merck	NA
Atg9bFP: GAAGGACTCCGCGGTTC; RP: CCAGCTGGAAGACATCCTC; Probe: CAGGCAAAGCCATTCCGCTGATGATAGC	This study; synthesised by Merck	NA
Atp6v0a2	Thermo Fisher Scientific	Mm00441846_g1
Atxn3; FP: CCATAAGTCGCCAGGAAATCG; RP: AACTACCTTGCATACTGAGCTG; Probe: GGAGGATGAAGAAGCTGATCTCCGCA	This study; synthesised by Merck	NA
Barhl; FP: AATACCTGAGCGTGCAAGAC; RP: TTCCATTTAGTCCTGCGGTTC; Probe: GGAGCTAGCCGCCTCGCTCA	This study; synthesised by Merck	NA
Cacna1g	Thermo Fisher Scientific	Mm00486572_m1
Calca	Thermo Fisher Scientific	Mm004801463_m1
Calcr	Thermo Fisher Scientific	Mm00432282_m1
Ccdc164	Thermo Fisher Scientific	Mm01210710_m1
Cd38; FP: AACCATACCATGTAACAAGACTCTC; RP: TCATATCAGAAGTACTAGGGTCTCC; Probe:TGGAGGACACCCTGCTGGGC	This study; synthesised by Merck	NA
Cep110; FP: GCTCCGGCAAATGGATAAGATG; RP: GCATTCTGTCCTTGGTTTGGC; Probe: AAAAGTCCACGAGTGGCGCTTTCAG	This study; synthesised by Merck	NA
Chrm1; FP: CCTCCCCTATCCACCTTCAGC; RP: GGCATCACCTTGGCATCCAG; Probe: AGGCTGCTGGCCAGATGGACA	This study; synthesised by Merck	NA
Cln6	Thermo Fisher Scientific	Mm01179411_m1
Coch	Thermo Fisher Scientific	Mm0000483360_m1
Creb3l1; FP: CTCACTGCCCCTCACAAACT; RP: CCTTCTCCTCAGCCTTGGTG; Probe: CATCAGGGCCACTGCTCTTGACAGAAG	This study; synthesised by Merck	NA
Ctns; FP: ATGAGGAGGAATTGGCTGCTT; RP: ACGTTGGTTGAACTGCCATTTT; Probe: TGGGAGGAGCAGTGAGACTGACAGTTG ACT	This study; synthesised by Merck	NA
Dapk2	Thermo Fisher Scientific	Mm00802402_m1
Dock2	Thermo Fisher Scientific	Mm00473720_m1
Eef1a; FP: CACATCCCAGGCTGACTGT; RP: TCGGTGGAATCCATTTTGTT; Probe: CCTGATTGTTGCTGCTGGTGTTGGTGAA	This study; synthesised by Merck	NA
Fgf10; FP: GTTGTTGCCGTCAAAGCCAT; RP: TGTGCTGCCAGTTAAAAGATGC; Probe: AGCCATGAACAAGAAGGGGAAACTCTAT GGCT	This study; synthesised by Merck	NA
Fmnl3; FP: CAGCAAGGTGACCCTTTTGGA; RP: ACGAAGTCTACAGGTAGTGTCTG; Probe: TGCCAAGAACCTGGCTATCACCCTTCGCA	This study; synthesised by Merck	NA
Gapdh; FP: AGGTCGGTGTGAACGGATTTG; RP: TGTAGACCATGTAGTTGAGGTCA; Probe: CCTGGTCACCAGGGCTGCCATTTG	This study; synthesised by Merck	NA
Gpr155	Thermo Fisher Scientific	Mm01327379_m1
Hyls1; FP: CGAGTGGGAAACAGCGTTC; RP: AAACCTTGAACGCCAGAACC; Probe: GCCGGAGCGGTGGACCTTTC	This study; synthesised by Merck	NA
iI13ra1	Thermo Fisher Scientific	Mm01302068_m1
Inca1; FP: GCACGGACGCTCTCTTCTC; RP: TGACCACTCTGGAACACTTGG; Probe: GTTTGACTCCCGGACCGCCG	This study; synthesised by Merck	NA
Mfsd10; FP: GGTGGGAAGCAAAGGTTTCG; RP: GACCCTAGGGCAGCAGTAAG; Probe: TCAGGGCGCCTACGACTCCC	This study; synthesised by Merck	NA
Ramp2; FP: CTGAGGACAGCCTTGTGTCAA; RP: ATTCCAAGCAGTTCTGCAGGT; Probe: TGGAAGACTACGAAACACATGTCCTACCT TGCTG	This study; synthesised by Merck	NA
Sfrp4	Thermo Fisher Scientific	Mm004840104_m1
Slc2a5	Thermo Fisher Scientific	Mm00600311_m1
Smad3; FP: GGAGAAGTGGTGCGAGAAGG; RP: TGACCTGGGGATGGTAATGC; Probe: CGGGGCAGTTGGACGAGCTG	This study; synthesised by Merck	NA
ST8SIA1; FP: TGGGAAACGGTGGGATTCTG; RP: TGGCGAATTATGCTGGGGTT; Probe: AGTGGCTGTGGCCGTCAAATAGATGAAG C	This study; synthesised by Merck	NA
Stk36	Thermo Fisher Scientific	Mm00558201_m1
Taslrl; FP: CAGGTCTGACAGCTTCAACGG; RP: AGGGTGATGTTGGGAAGCAG; Probe: CCAAGCCATGCGGTTCACCGT	This study; synthesised by Merck	NA
Tbrg4	Thermo Fisher Scientific	Mm01220234_g1
Tm9sf1	Thermo Fisher Scientific	Mm00446042_m1
Tnr; FP: AGGCTGGTTTGGCAAGAACT; RP: TCATCTCCGCTGTATTCACTGT	This study; synthesised by Merck	NA
Vash2; FP: TCCAACCCAAGTGAGAAGCC; RP: GTCAGAGCAGGCGATTTGTC; Probe: TCCAAGGAGGCTCGGCAGGC	This study; synthesised by Merck	NA
Zfp709	Thermo Fisher Scientific	Mm00521284_m1
Zic1; FP: GCAAGCCCTTCAAAGCCAAA; RP: CTTCTCCCCTGTGTGTGTCC;Probe: CAACCACATCCGAGTGCACACAGGCGAA	This study; synthesised by Merck	NA
**Oligonucleotides for T7E1 mutation detection assay PCR**		
Cacna1g-Ex8; FP: CACACAGTTCTCTGAGACCAAG; RP: ATACCTTCCCACTACCCACA	This study; synthesised by Merck	NA
Slc35f4: FP: TTATCACCGCACTGCTGTTTCC; RP: CTCCTGAAATCCACACAGATCC	This study; synthesised by Merck	NA
Nipal3: FP: AGTCACCCTCTTTATTTCCTCG; RP: ATACTTGAAAGGCAGTTTGGGC	This study; synthesised by Merck	NA
Trpm5: FP: ACTTTGTGTCTTGGGGGAAACC; RP: AGGGTCTTCACCATTACCTTGG	This study; synthesised by Merck	NA
Sema3b: FP: AGGAAACTTGAGGACGGCAAGG; RP: GGCTTTCTTGTGTTCTGGAAGC	This study; synthesised by Merck	NA
Col6a5: FP: GAGGCAGAACCGTGGTCTAT; RP: ATATTAGCCAGAGCGGGCCT	This study; synthesised by Merck	NA
**Plasmids**		
halGa-pDsRed	Addgene	Cat# 45811
pCMV-hCav3.1 (full-length)-HA	This study	NA
pCMV-truncated hCav3.1 (Domain I)-HA	This study	NA
pCMV-truncated hCav3.1 (Domain II)-HA	This study	NA
pCMV-truncated hCav3.1 (Domain III)-HA	This study	NA
pCMV-truncated hCav3.1 (Domain IV)-HA	This study	NA
pCAG-hCav3.1-ires-EGFP	This study	NA
pCMV-hCav3.1-ires-Puro	This study	NA
pCMV-hCav3.1 (M948A/T949A)-ires- Puro	This study	NA
pCMV-hCav3.1 (V841A/V845A/V851A)-ires-Puro	This study	NA
pCMV-hCav3.1 (E789A/V815A/R846A)-ires-Puro	This study	NA
pCMV-hCav3.1 (D902A/K927AA)-ires- Puro	This study	NA
pCMV-hTRPC5	Sino Biological	Cat# HG18741-UT
**Rodent diets**		
Rodent chow	SAFE	Cat# SAFE R105
60% high fat diet (HFD)	Research Diets	Cat# D12451i
14% protein diet	Research Diets	D21071201i
45% protein diet	Research Diets	D17030703i
**Software and algorithms**		
Prism 10.0	GraphPad	https://www.graphpad.com/scientific-software/prism/
ImageJ (Fiji)	ImageJ	https://imagej.nih.gov/ij/index.html
Ingenuity Pathway Analysis	Qiagen	
Harmony	PerkinElmer	
Zeiss ZEN 2.3	Zeiss	
Quantity One	Bio-Rad	
pCLAMP	Molecular Devices	
Clampfit	Molecular Devices	
CRISPick	Broad Institute	https://portals.broadinstitute.org/gppx/crispick/public
AutoDock Vina 1.1.2	Scripps Research Institute	https://vina.scripps.edu/
UCSF Chimera	UCSF	https://www.cgl.ucsf.edu/chimera/
GOLD suite version 5.3	The Cambridge Crystallographic Data Centre (CCDC)	https://www.ccdc.cam.ac.uk/solutions/software/gold/
MOE version 2019	Chemical Computing Group	https://www.chemcomp.com/en/Products.htm
PLIP (Protein Ligand Interaction Profiler) server	Biotechnology Center TU Dresden (BIOTEC)	https://plip-tool.biotec.tu-dresden.de/plip-web/plip/index
**Deposited data**		
PhosphoTRAP RNAseq data	ArrayExpress	E-MTAB-16665
Source Data	This paper	Data S1

## Supplementary Material

Supplementary materials

## Data Availability

Data used to generate the figures are available in Data S1. RNA sequencing data of PhosphoTRAP are available on ArrayExpress (https://www.ebi.ac.uk/biostudies/arrayexpress), accession number: E-MTAB-16665. Microscopy data and raw electrophysiology data reported in this paper will be shared by the lead contact upon request. Any additional information required to re-analyse the data reported in this paper is available from the lead contact upon request.
