# Supplementary material for: Cav3.1 Is a Neuronal Leucine Sensor That Mediates Satiety and Weight Loss in Response to Dietary Protein
Source: Cell Metab. Author manuscript; Available in PMC 2026 Oct 16. (PMC7619568; doi:10.1016/j.cmet.2026.03.017)
Supplement: Supplementary file 1 [file EMS217845-supplement-Supplementary_materials.pdf]

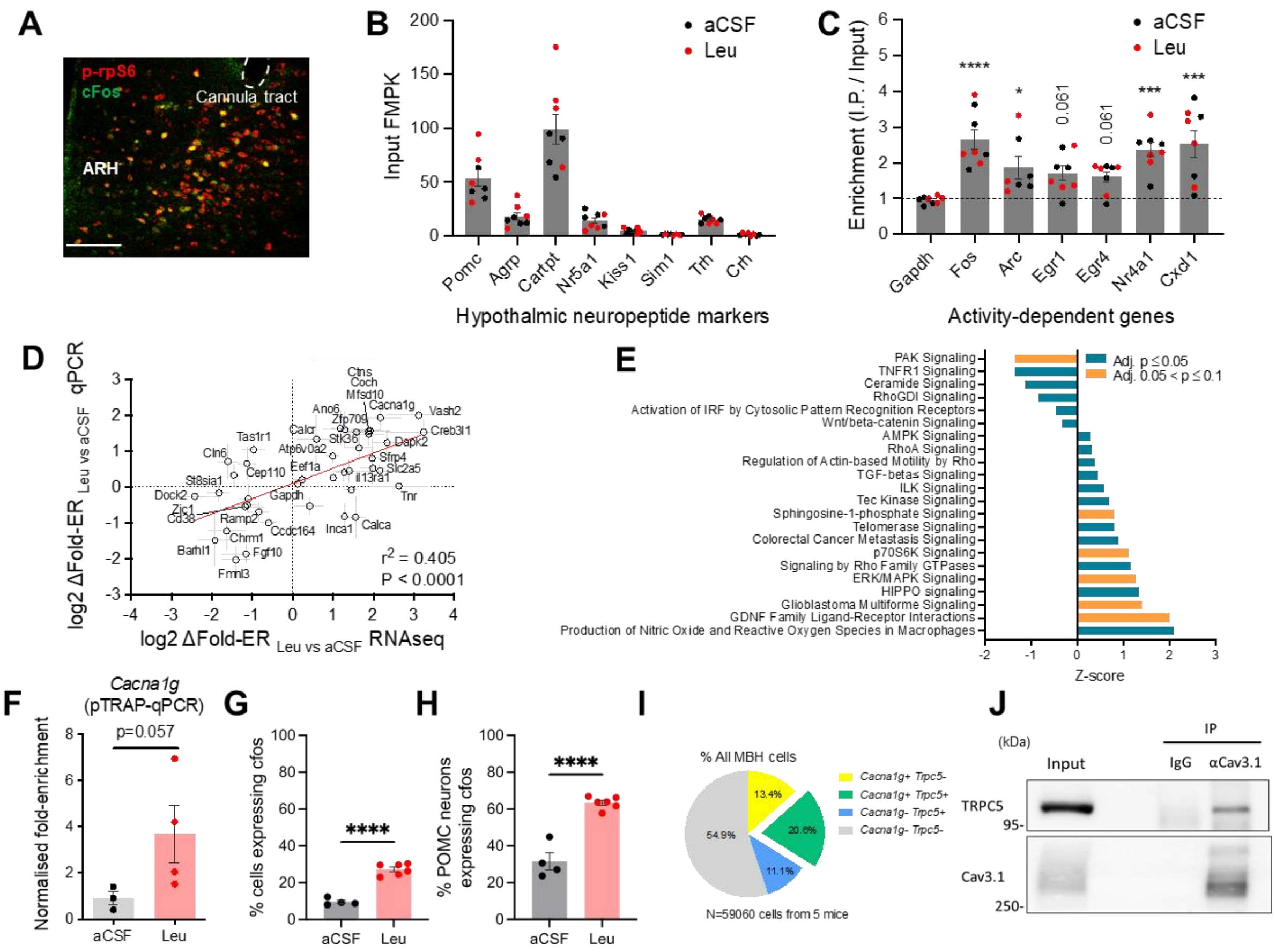

Figure S1: Additional data related to Figure 1

(A) Representative image showing co-localisation of phospho-rpS6 and cFos in the ARH and VMH after MBH leucine injection. Scale bar: 50 µm. **(B)** Abundance of hypothalamic marker transcripts in MBH input samples from both aCSF and leucine groups. **(C)** Enrichment (I.P./Input) of activity dependent genes in MBH samples from both aCSF and leucine groups compared to *Gapdh*. **(D)** Validation of PhosphoTRAP RNAseq results with RT-qPCR of 41 selected genes. **(E)** Ingenuity Pathway analysis (IPA) of transcripts enriched in leucine-responsive neurons. See Table S5 for full data. **(F)** Normalised fold enrichment ratio of *Cacna1g* analysed by RT-qPCR. The RNA samples used were from the same as the ones used for RNAseq, n = 3/4. **(G,H)** RNAscope quantification - see Figure 1C. **(G)** % all MBH cells expressing *cfos*, (**H**) % all POMC neurons expressing *cfos*, **(I)** number of *Cacna1g* spots per POMC neuron, n = 4/6. **(I)** Relative proportion of MBH cells expressing *Cacna1g* and *Trpc5*, n = 59060 MBH cells from 5 WT mice. **(J)** Representative Western blot of co-immunoprecipitation assay of heterologously expressed human TRPC5 and Cav3.1 in HEK293 cells. This experiment has been repeated twice. Data reported as mean ± SEM. *p<0.05, ***p<0.001, ****p<0.0001. Statistics: 1-way ANOVA with Holm-Sidak post-test: C; Pearson correlation: D; unpaired t-test: F-H.

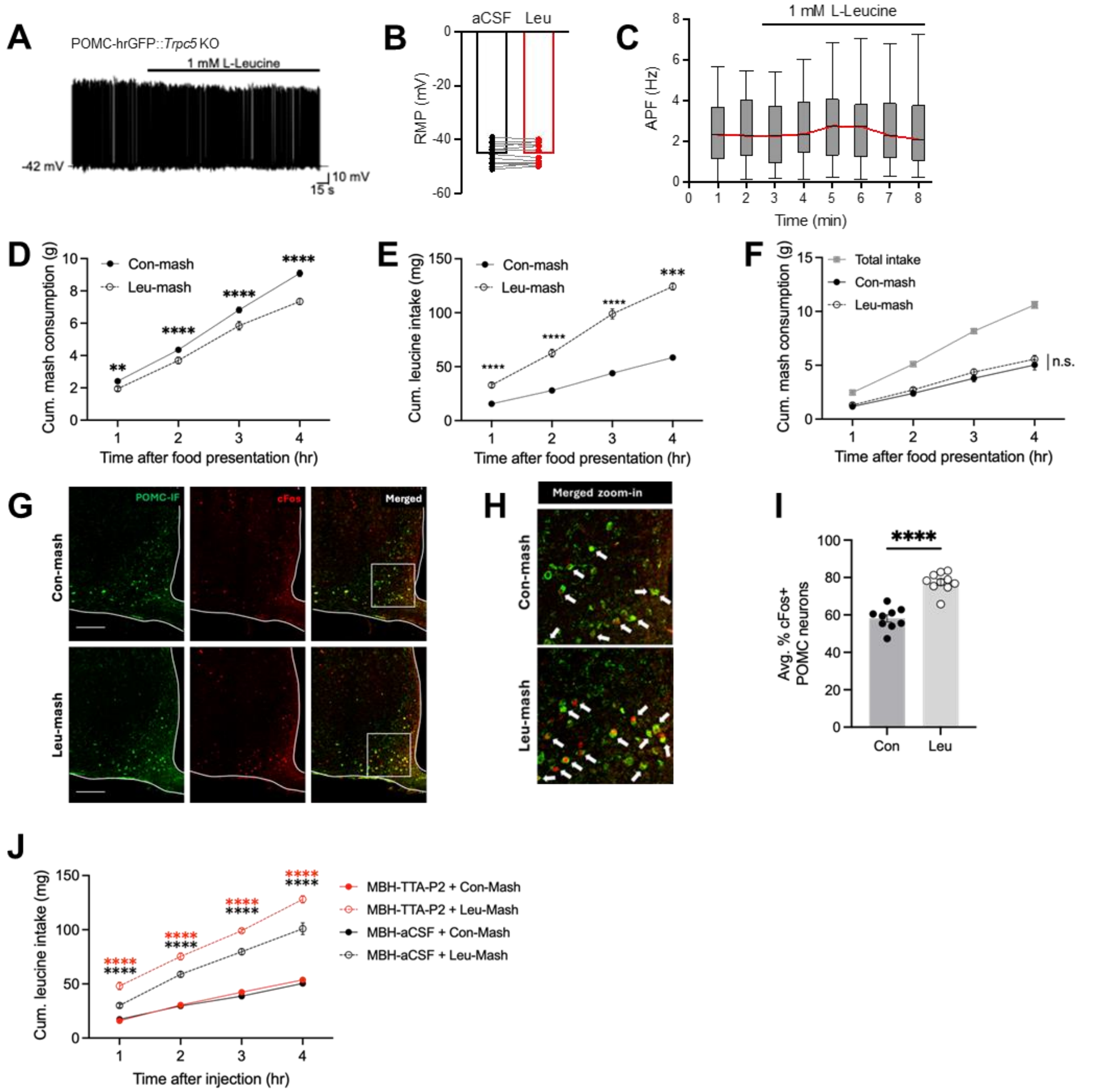

Figure S2: Additional data related to Figure 2

(A-C) Brain slice current-clamp recordings of arcuate POMC neurons from *Trpc5* KO mice with leucine treatment. **(A)** Representative trace showing that 1 mM leucine fails to induce a depolarization of POMC neurons on a *Trpc5* KO background. **(B)** Acute effect of leucine on the membrane potential of POMC neurons deficient for TRPC5 (n=14). **(C)** APF of POMC neurons deficient for TRPC5 over time (n=13). **(D,E)** Cumulative mash consumption **(D)** and leucine intake **(E)** of mice fed a control (Con) and leucine-supplemented mash (31.5 mg/g), n=19 per group. **(F)** Cumulative intake of Leu-mash and Con-mash during a 4hr self-selection paradigm, no preference was observed, n=10. **(G-I)** Representative images **(G,H)** and quantification **(I)** of cfos (red) expression in ARH POMC neurons (green) following leucine-supplemented mash feeding. Scale bar: 200 µm, n=9/10. **(J)** Cumulative leucine intake after MBH TTA-P2 injection in experiment depicted in Fig. 2N. n=6. Values in (B,D,E,F,I,J) are mean ± SEM. Values in box plots (C) are median ± range and comparisons were made versus the minute bin prior leucine

treatment.**p<0.01, ***p<0.001, ****p<0.0001. Statistics: unpaired t-test: B,I; Friedman test followed by Dunn's post-test: C; repeated 2-way ANOVA followed by Holm-Sidak post-test: D,E,F,J.

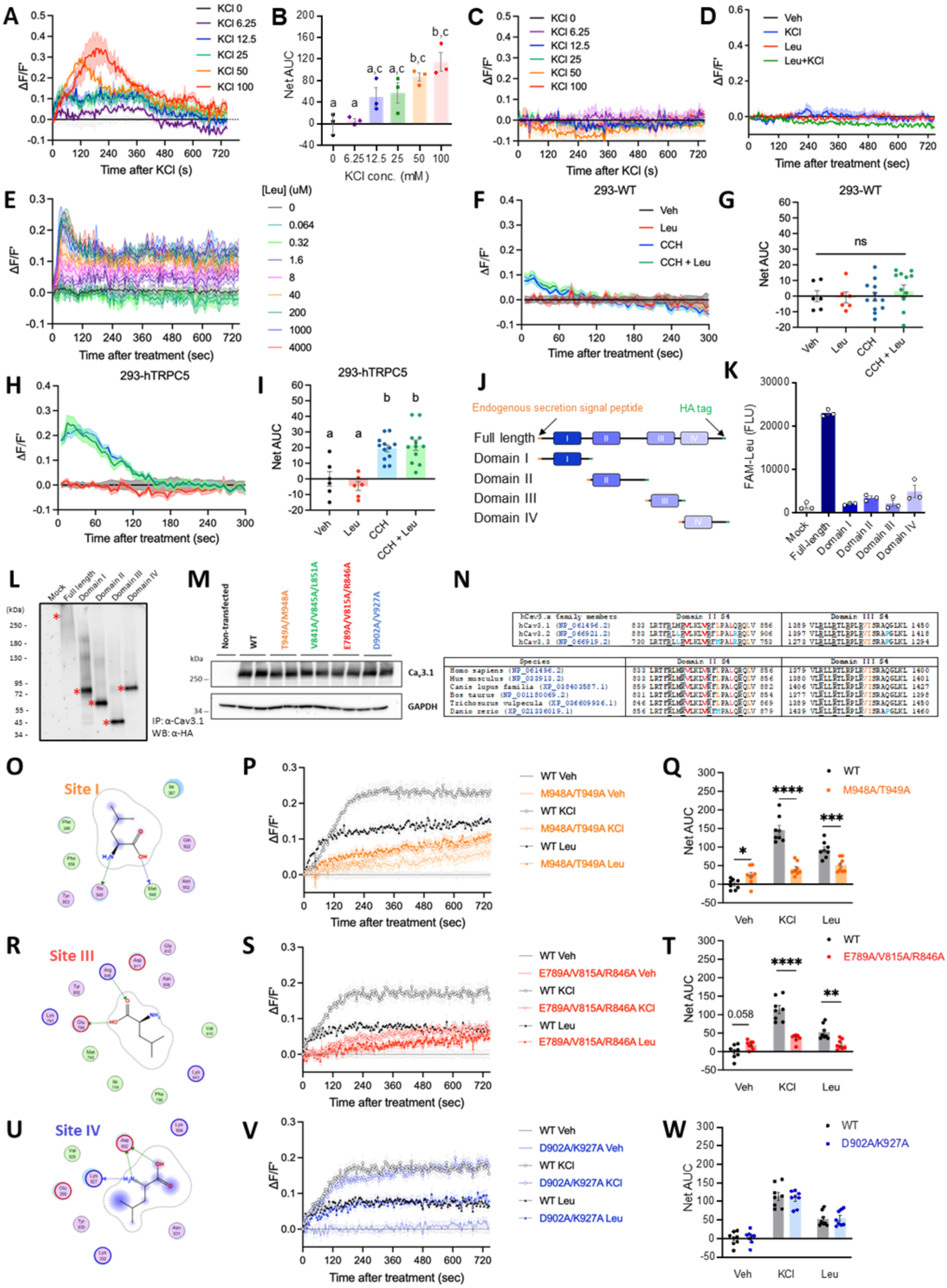

Figure S3: Additional data related to Figure 3

(A-E) Fluo8 Ca^{2+} flux assay of HEK293-hCa$_v$3.1 and -WT cells. Dose-dependent KCl-induced Ca^{2+} responses and AUC quantification of HEK293-hCa$_v$3.1 **(A-B)**; Lack of Ca^{2+} responses in non-transfected HEK293-WT cells after various doses of KCl **(C)** and leucine (1 mM) treatment (**D**), n = 3 per group. Dose-dependent Leu-induced Ca^{2+} response traces in the

experiment depicted in Fig. 3G **(E)**. **(F-I)** Fluo8 Ca^{2+} flux assay of HEK293-WT and -hTRPC5 overexpressing cells responses to the acetylcholine receptor agonist, carbachol (CCH; 200 µM) and leucine (1 mM). Ca^{2+} responses and AUC quantification after CCH and Leu treatments in 293-WT **(F,G)** and 293-hTRPC5 cells **(H,I)**, n = 6 for Veh and Leu groups and n = 12 for CCH and CCH+Leu groups, merged from 2 independent experiments **(J-L)** In vitro fluorescent leucine binding assay of individual $hCa_v3.1$ domains. Schematic diagram of recombinant truncated hCav3.1 domain fragments construction **(J)**. Quantification of fluorescence measurements for fluorescent leucine binding **(K)**. Post-assay Western blot validates successful expression and immunoprecipitation of the recombinant domain fragments **(L)**. Red asterisks indicate expected band size of the fragments. Note that this assay was performed on transiently transfected cells; the full-length $hCa_v3.1$ expression was far weaker yet bound stronger to FAM-leucine compared to the truncated fragments. Data represent 3 technical replicates upon fluorescence measurements. These experiments have been repeated twice with similar results. Data of a representative experiment is shown. **(M)** Western blot validation for the protein expression of hCav3.1 Site I/II/III/IV mutants in respective stably expressing HEK293 cells. **(N)** Amino acid sequence alignments of the Domain II and III voltage-sensing S4 transmembrane segments of human Cav3.1 against hCav3.2 and hCav3.3 (upper panel) and against Cav3.1 of various mammalian species and zebrafish (lower panel). Bolded underlined black "R": consensus voltage-sensing arginine residues; bolded red: critical residues predicted to mediate leucine binding and were chosen for mutagenesis in this study; orange: other residues predicted to participate in leucine binding; bolded blue: residues different from human Cav3.1.**(O,R,U)** 2D ligand interactions diagrams for the docked poses of L-Leu at Site I **(O)**, Site III **(R)** and Site IV **(U)**. The residues mutated for experimental validation are shown with asterisks. The dotted arrow signs indicate hydrogen bonding whilst the dotted contour around the ligand pose indicate hydrophobic interactions with residues shown. **(P-Q, S-T, V-W)** Fluo8 Ca^{2+} flux assay of HEK293-$hCa_v3.1$ predicted Leu-binding mutant cells. Ca^{2+} responses and AUC quantification after KCl and Leu treatments of Site I mutant cells (M948A/T949A; **P-Q**); Site III mutant cells (E789A/V815A/R846A; **S-T**) and Site IV mutant cells (D902A/K927A; **V-W**), n = 8 merged from 4 independent experiments with duplicates each. Values and Ca^{2+} response traces are reported as mean ± SEM. *p<0.05, **p<0.01, ***p<0.01, ****p<0.0001. Groups denoted with different letter in (B,G,I) indicate significant difference (p<0.05). Statistics: 1-way ANOVA with Holm-Sidak post-test: B,G,I; unpaired t-test: Q,T,W.

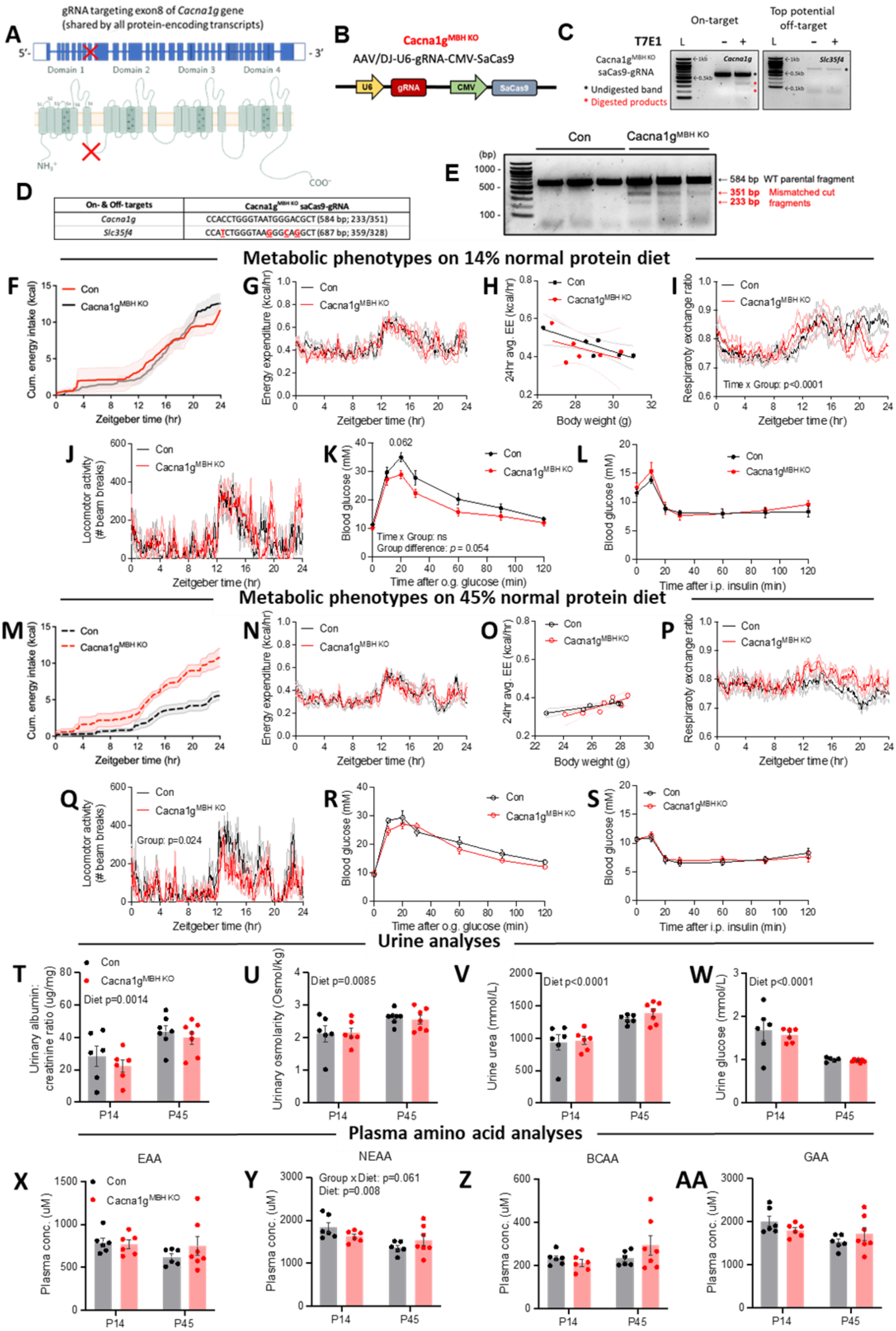

A
gRNA targeting exon8 of Cacna1g gene
(shared by all protein-encoding transcripts)
5'-
-3'
Domain 1
Domain 2
Domain 3
Domain 4
NH3+
COO-
B
Cacna1gMBH KO
AAV/DJ-U6-gRNA-CMV-SaCas9
U6
gRNA
CMV
SaCas9
C
T7E1
On-target
Top potential off-target
Cacna1gMBH KO saCas9-gRNA
* Undigested band
* Digested products
Cacna1g
Slc35f4
D
On- & Off-targets | Cacna1gMBH KO saCas9-gRNA
Cacna1g | CCACCTGGGTAATGGGACGCT (584 bp; 233/351)
Slc35f4 | CCATCTGGGTAAGGGCAGGCT (687 bp; 359/328)
E
Con
Cacna1gMBH KO
(bp)
1000
500
100
584 bp WT parental fragment
351 bp
233 bp
Mismatched cut fragments
Metabolic phenotypes on 14% normal protein diet
F
Con
Cacna1gMBH KO
Cum. energy intake (kcal)
Zeitgeber time (hr)
G
Energy expenditure (kcal/hr)
H
24hr avg. EE (kcal/hr)
Body weight (g)
I
Respiraroty exchange ratio
Time x Group: p<0.0001
J
Locomotor activity (# beam breaks)
K
0.062
Blood glucose (mM)
Time x Group: ns
Group difference: p = 0.054
Time after o.g. glucose (min)
L
Time after i.p. insulin (min)
Metabolic phenotypes on 45% normal protein diet
M
N
O
P
Q
Group: p=0.024
R
S
Urine analyses
T
Urinary albumin: creatinine ratio (ug/mg)
Diet p=0.0014
P14
P45
U
Urinary osmolarity (Osmol/kg)
Diet p=0.0085
V
Urine urea (mmol/L)
Diet p<0.0001
W
Urine glucose (mmol/L)
Diet p<0.0001
Plasma amino acid analyses
X
EAA
Plasma conc. (uM)
Y
NEAA
Group x Diet: p=0.061
Diet: p=0.008
Z
BCAA
AA
GAA

Figure S4: Additional data related to Figure 4

(A) Diagram of the CRISPR knockout strategy targeting the exon 8 of mouse *Cacna1g* gene which is common to all protein-coding splice variants. The red crosses indicate the intended mutation sites on genomic DNA and protein. **(B)** Schematic representation of the AAV constructs used for Cacna1g$^{MBH\ KO}$ models. **(C)** Validation for the specificity of gRNAs used to target mouse *Cacna1g.* Representative DNA electrophoresis images of T7 endonuclease I mutation detection assay of genomic DNA isolated from mouse mHypoE-N46 cell line 96 hr after transfection of indicated gRNA/Cas9 all-in-one plasmids. Intended *Cacna1g* targeting site and top predicted exonic off-target sites were analysed. Black arrows indicate parental unmodified PCR amplicons and red arrows indicate digested mutated fragments of expected size. L: 0.5 µg 100 bp DNA ladder; +/-: with or without T7E1 digestion. **(D)** Table of the on- and predicted off-targets genomic DNA sequence for the gRNAs. The expected DNA size of parental amplicons and digested fragments are indicated in the brackets. **(E)** Representative DNA electrophoresis image of T7 endonuclease I mutation detection assay of genomic DNA isolated from the MBH tissues of Con and Cacna1g$^{MBH\ KO}$ mice. Black arrow indicates parental unmodified PCR amplicons and red arrows indicate digested mutated fragments of expected size. **(F-L)** Metabolic parameters of Con and Cacna1g$^{MBH\ KO}$ mice on P14 diet during indirect calorimetry measurement. 24-hr profile of cumulative energy intake **(F)**, 24-hr profiles of energy expenditure **(G)**, ANCOVA analysis of average 24-hr energy expenditure against body weight **(H)**, 24-hr profile of respiratory exchange ratio **(I)**, 24-hr profile of locomotor activity **(J)**, Oral glucose tolerance test **(K)** and insulin tolerance test **(L)**. **(M-S)** Metabolic parameters of Con and Cacna1g$^{MBH\ KO}$ mice on P45 diet during indirect calorimetry measurement. 24-hr profile of cumulative energy intake **(M)**, 24-hr profiles of energy expenditure **(N)**, ANCOVA analysis of average 24-hr energy expenditure against body weight **(O)**, 24-hr profile of respiratory exchange ratio **(P)**, 24-hr profile of locomotor activity **(Q)**, Oral glucose tolerance test **(R)** and insulin tolerance test **(S)**. **(T-W)** Urine analyses of Con and Cacna1g$^{MBH\ KO}$ mice fed on P14 and P45 diets. Urinary albumin/creatinine (ACR) ratio **(T)**, osmolarity **(U)**, urea **(V)** and glucose **(W)**. **(X – AA)** Grouped post-absorptive phase plasma amino acid profiling of Con and Cacna1g$^{MBH\ KO}$ mice fed on P14 and P45 diets. Essential amino acid (EAA; **X**), non-essential amino acid excluding glycine (NEAA; **Y**), branched-chain amino acid (BCAA, **Z**) and glucogenic amino acid excluding glycine (GAA, **AA**). Individual amino acid data are shown in **Table S7**. Con-P14: n = 6; Con-P45: n = 6; KO-P14: n = 7; KO-P45: n = 7. Values are reported as mean ± SEM. Statistics: repeated 2-way ANOVA with Holm-Sidak post-test: K,L,R,S; 2-way ANOVA with Holm-Sidak post-test: T-AA.

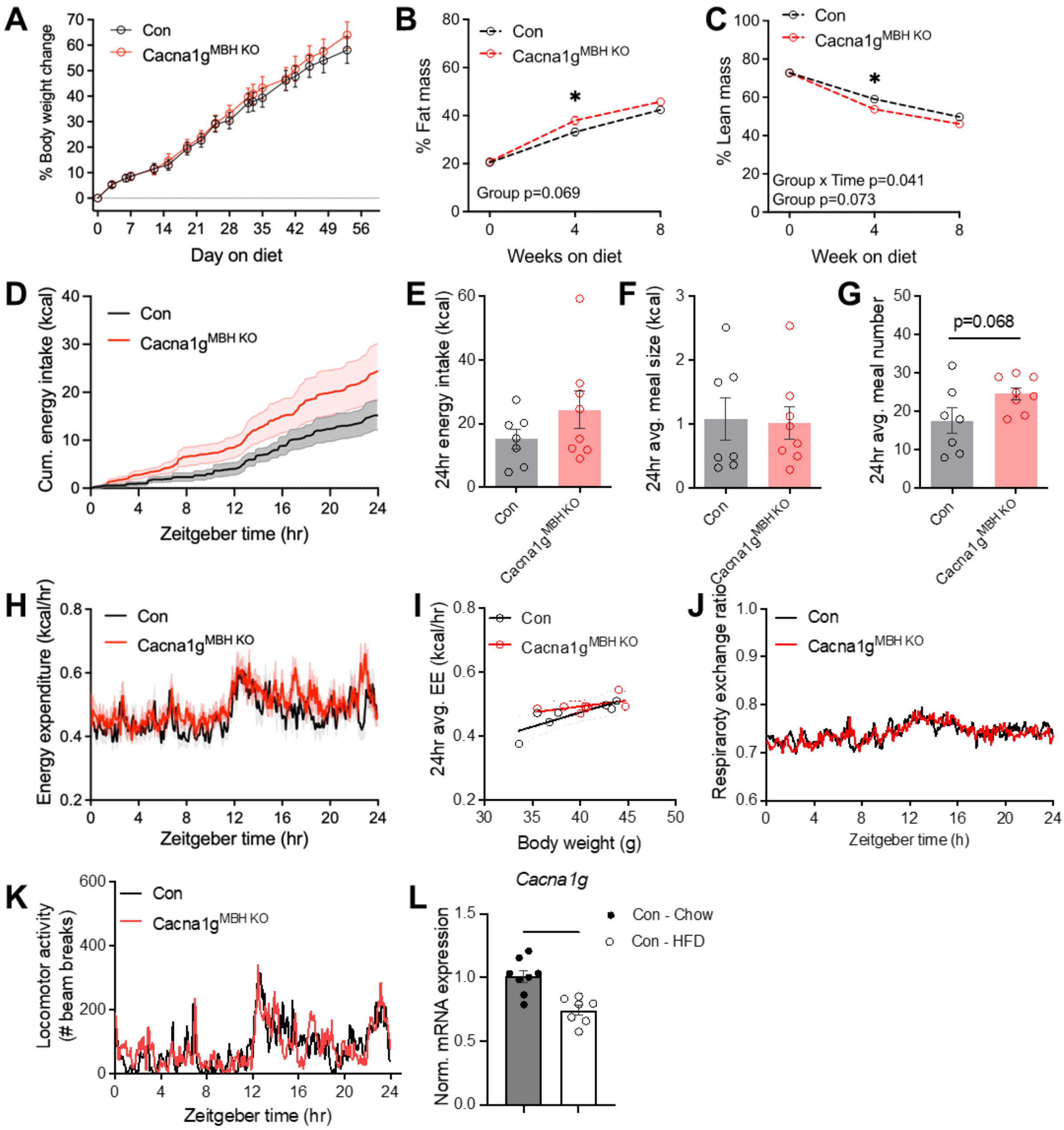

Figure S5: Hypothalamic *Canca1g* is downregulated by diet-induced obesity, related Figure 4

(A-K) Metabolic phenotyping of Con and Cacna1g$^{MBH\ KO}$ mice on 60% high fat diet (HFD). % body weight change **(A)**, % fat mass **(B)** and % lean mass **(C)** over the course of 8 weeks HFD feeding. 24-hr energy intake profile **(D),** 24-hr total energy intake **(E)**, 24-hr average meal size **(F)**, 24-hr average meal number **(G)**, 24-hr profiles of energy expenditure **(H)**, ANCOVA analysis of 24-hr average energy expenditure against body weight **(I)**, 24-hr profile of respiratory exchange ratio **(J)** and 24-hr profile of locomotor activity **(K)**, during indirect calorimetry measurement. **(L)** RT-qPCR measurement of relative expression of *Cacna1g* mRNA in the mediobasal hypothalamus of Con mice fed on HFD vs chow for 8 weeks. Con-Chow: n = 8; Con-HFD: n = 7; KO-Chow: n = 7; KO-HFD: n = 8. *p<0.05. **p<0.01. Values are reported as mean ± SEM. Statistics: repeated 2-way ANOVA with Holm-Sidak post-test: A-C; unpaired t-test: E-G, L; ANCOVA: I.

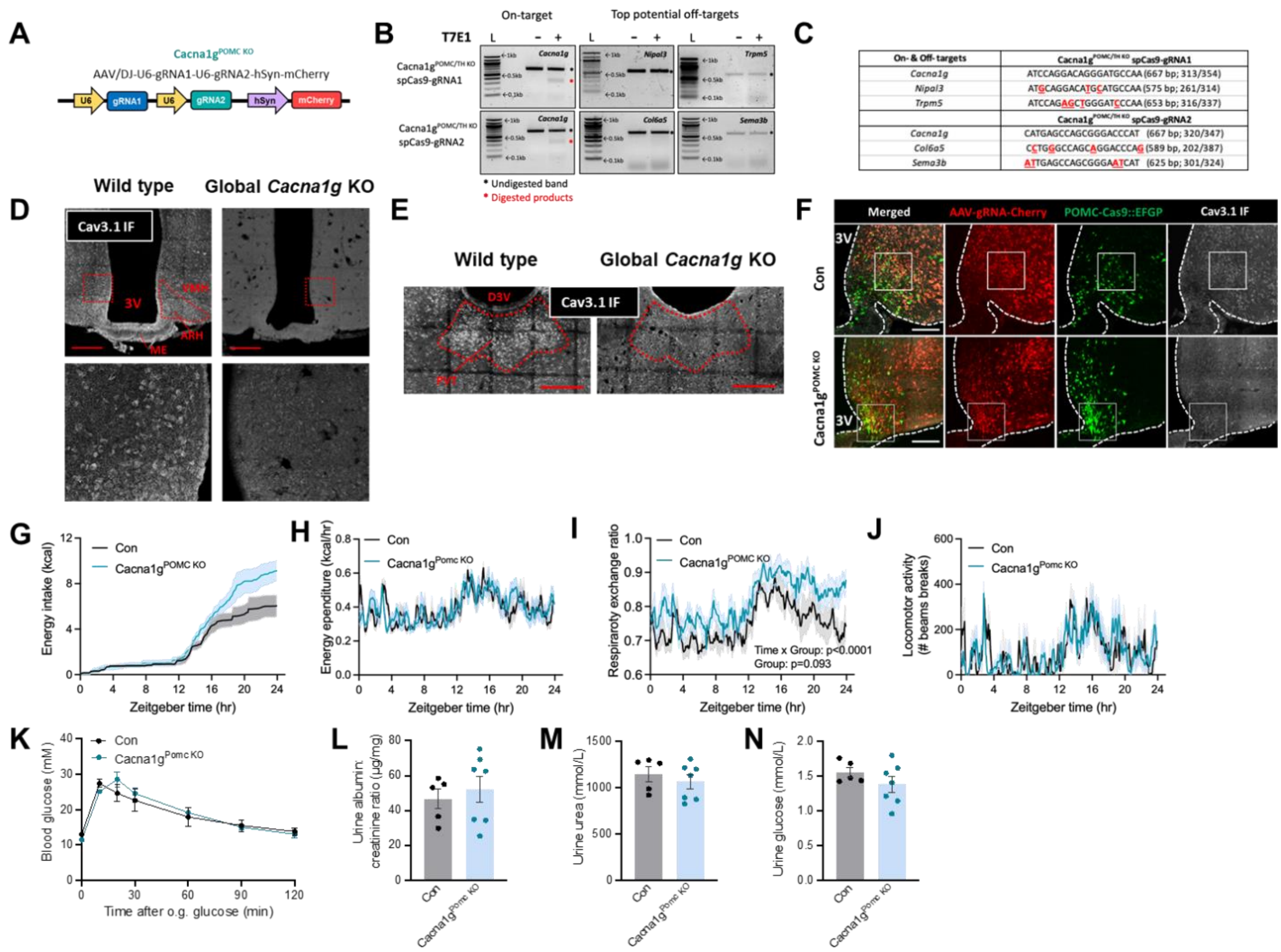

Figure S6: Additional data related to Figure 5

(A) Schematic representation of the AAV constructs used for Cacna1g$^{POMC\ KO}$ models. **(B)** Validation for the specificity of gRNAs used in Cacna1g$^{POMC\ KO}$ mice. Representative DNA electrophoresis images of T7 endonuclease I mutation detection assay of genomic DNA isolated from mouse mHypoE-N46 cell line 96 hr after transfection of indicated gRNA/Cas9 all-in-one plasmids. Intended *Cacna1g* targeting site and top predicted exonic off-target sites were analysed. Black arrows indicate parental unmodified PCR amplicons and red arrows indicate digested mutated fragments of expected size. L: 0.5 µg 100 bp DNA ladder; +/-: with or without T7E1 digestion. **(C)** Table of the on- and predicted off-targets genomic DNA sequence for the gRNAs. The expected DNA size of parental amplicons and digested fragments are indicated in the brackets. **(D-E)** Confocal immunofluorescence (IF) microscopy validation of a C-terminal specific anti-Cav3.1 antibody (Abcam, Cat# ab203577) with constitutive global *Cacna1g* KO mice. Representative IF images of the MBH of WT and global KO mice **(D)**. Upper panel: multi-focal plane (maximum intensity projection) images. Lower panel: single focal plane zoom-in images of the regions highlighted in red squares of the respective pictures shown on the upper panel. Representative IF images of the thalamus of WT and global *Cacna1g* KO mice **(E)**. 3V: third ventricle; ARH: arcuate nucleus of the hypothalamus; VMH: ventromedial nucleus of the hypothalamus; ME: median eminence; D3V: dorsal third ventricle; PVT: paraventricular nucleus of the thalamus. Scale bars: 200 µm. **(F)** Zoom-out confocal immunofluorescence microscopy image of Fig. 5B. **(G-K)** Metabolic parameters of

Cacna1g$^{POMC\ KO}$ mice on P45 diet. 24-hr profile of cumulative energy intake **(G)**, 24-hr profile of energy expenditure **(H)**, 24 hr profiles of respiratory exchange ratio **(I)** and 24 hr profiles of locomotor activity **(J)** during indirect calorimetry measurement. Con: n = 5; Cacna1g$^{POMC\ KO}$: n = 7. Oral glucose tolerance test of Con and Cacna1g$^{MBH\ KO}$ mice on P14 diet **(K)**. Con: n = 5; Cacna1g$^{POMC\ KO}$: n = 7. **(L-N)** Urine analyses of Con and Cacna1g$^{POMC\ KO}$ mice fed on P45 diets. Urinary albumin/creatinine ACR ratio **(L)**, urea **(M)** and glucose **(N)**. Con: n = 5; Cacna1g$^{POMC\ KO}$: n = 7. Values are reported as mean ± SEM. Statistics: unpaired t-test: L, M-N; repeated 2-way ANOVA with Holm-Sidak post-test: K.

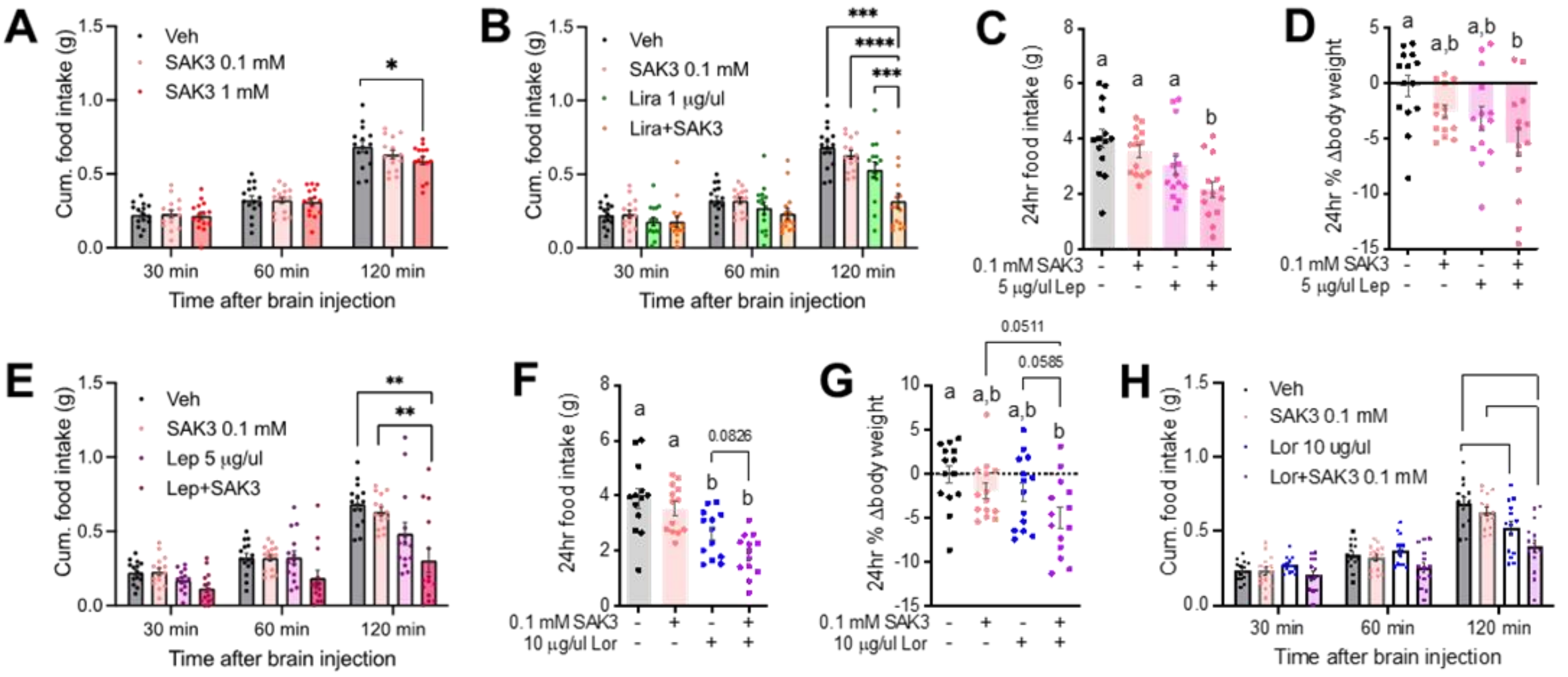

Figure S7: Additional data related to Figure 6

(A) Acute feeding responses after MBH injection of Veh, 0.1 mM and 1 mM $Ca_v3.1$ activator SAK3, n = 14 per group. **(B)** Acute feeding responses after MBH injection of Veh, 0.1 mM SAK3, 1 µg/µl liraglutide (Lira) and SAK3+Lira co-treatment, n = 14 per group. **(C-E)** 24-hr feeding responses **(C)**, 24-hr % body weight changes **(D)** and acute feeding responses **(E)** after MBH injection of Veh, 0.1 mM SAK3, 5ug/µl leptin (Lep) and SAK3+Lep co-treatment, n = 14 per group. **(F-H)** 24-hr feeding responses **(F)**, 24-hr % body weight changes **(G)** and acute feeding responses **(H)** after MBH injection of Veh, 0.1 mM SAK3, 10ug/µl lorcaserin (Lor) and SAK3+Lor co-treatment, n = 14 per group. Values are reported as mean ± SEM. *p<0.05, **p<0.01, ***p<0.001, ****p<0.0001. Groups denoted with different letter in (C, D, F, G) indicate significant difference (p<0.05). Statistics: repeated 2-way ANOVA with Holm-Sidak post-test: A,B,E,H; repeated 1-way ANOVA with Holm-Sidak post-test: C,D,F,G.

Supplemental Figure S1

Click here to access/download;Supplemental Text and Figures;Fig S1.tiff

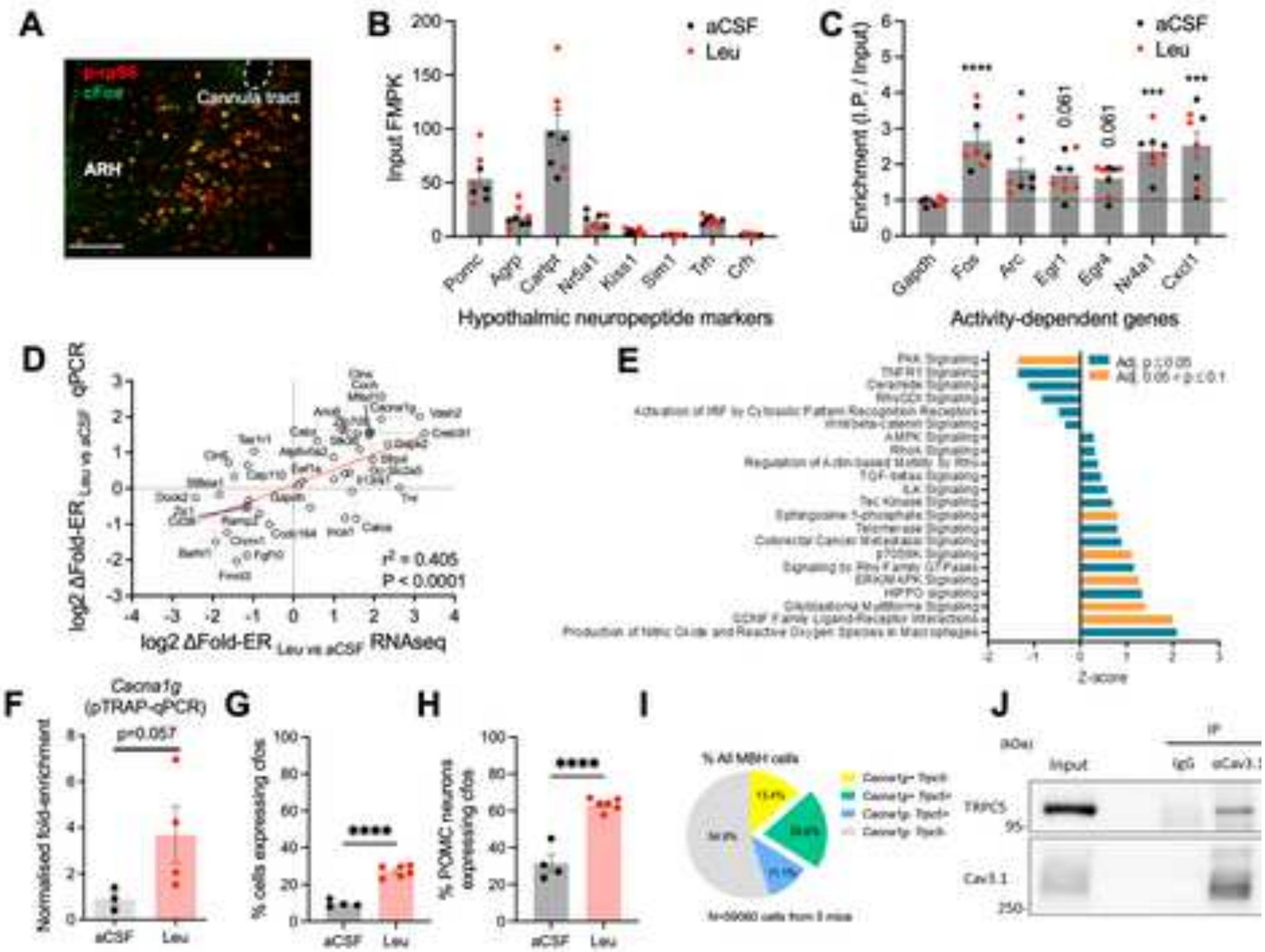

Supplemental figure S2

Click here to access/download;Supplemental Text and Figures;Figure S2.tiff

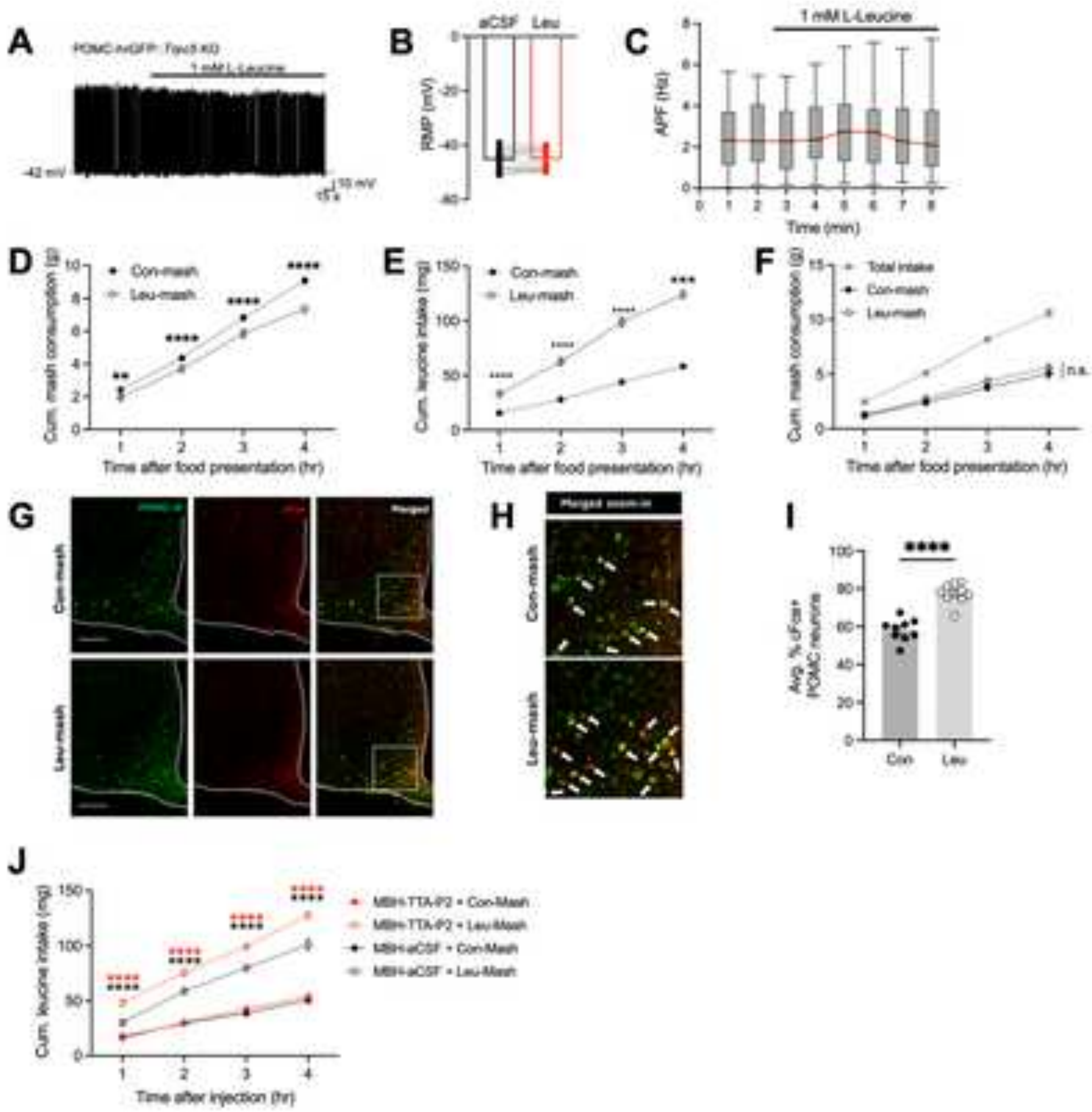

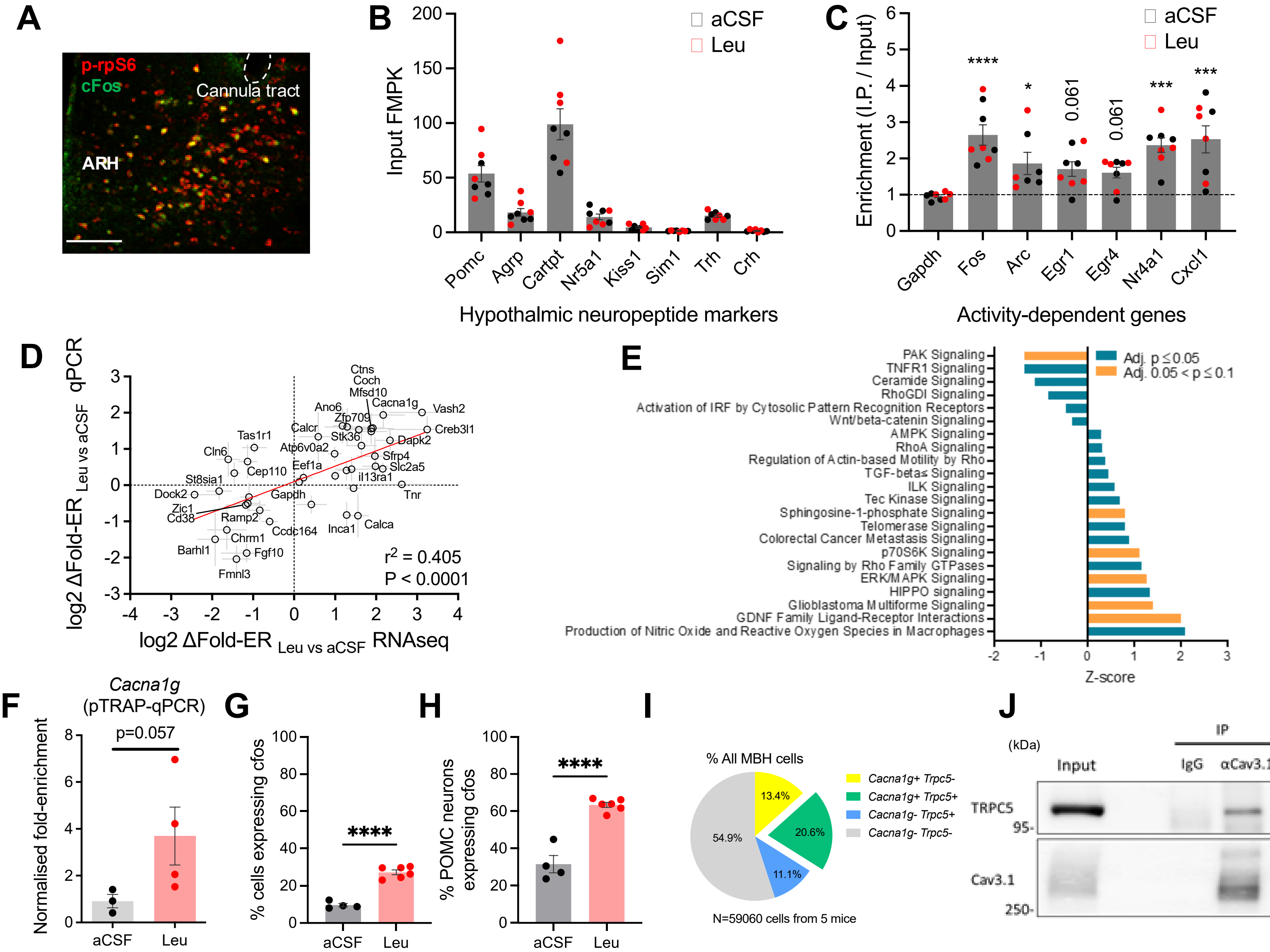
A
p-rpS6
cFos
Cannula tract
ARH
B
aCSF
Leu
Input FMPK
Pomc
Agrp
Cartpt
Nr5a1
Kiss1
Sim1
Trh
Crh
Hypothalmic neuropeptide markers
C
Enrichment (I.P. / Input)
****
*
0.061
0.061
***
***
Gapdh
Fos
Arc
Egr1
Egr4
Nr4a1
Cxcl1
Activity-dependent genes
D
log2 ΔFold-ER Leu vs aCSF qPCR
log2 ΔFold-ER Leu vs aCSF RNAseq
r2 = 0.405
P < 0.0001
Ctns
Coch
Mfsd10
Cacna1g
Vash2
Ano6
Zfp709
Creb3l1
Calcr
Stk36
Dapk2
Tas1r1
Atp6v0a2
Sfrp4
Cln6
Cep110
Eef1a
Slc2a5
St8sia1
Il13ra1
Dock2
Gapdh
Tnr
Zic1
Cd38
Ramp2
Ccdc164
Inca1
Calca
Chrm1
Barhl1
Fgf10
Fmnl3
E
PAK Signaling
TNFR1 Signaling
Ceramide Signaling
RhoGDI Signaling
Activation of IRF by Cytosolic Pattern Recognition Receptors
Wnt/beta-catenin Signaling
AMPK Signaling
RhoA Signaling
Regulation of Actin-based Motility by Rho
TGF-beta Signaling
ILK Signaling
Tec Kinase Signaling
Sphingosine-1-phosphate Signaling
Telomerase Signaling
Colorectal Cancer Metastasis Signaling
p70S6K Signaling
Signaling by Rho Family GTPases
ERK/MAPK Signaling
HIPPO signaling
Glioblastoma Multiforme Signaling
GDNF Family Ligand-Receptor Interactions
Production of Nitric Oxide and Reactive Oxygen Species in Macrophages
Adj. p ≤ 0.05
Adj. 0.05 < p ≤ 0.1
Z-score
F
Cacna1g
(pTRAP-qPCR)
p=0.057
Normalised fold-enrichment
aCSF
Leu
G
% cells expressing cfos
****
aCSF
Leu
H
% POMC neurons expressing cfos
****
aCSF
Leu
I
% All MBH cells
13.4%
20.6%
11.1%
54.9%
Cacna1g+ Trpc5-
Cacna1g+ Trpc5+
Cacna1g- Trpc5+
Cacna1g- Trpc5-
N=59060 cells from 5 mice
J
(kDa)
IP
Input
IgG
αCav3.1
TRPC5
95-
Cav3.1
250-

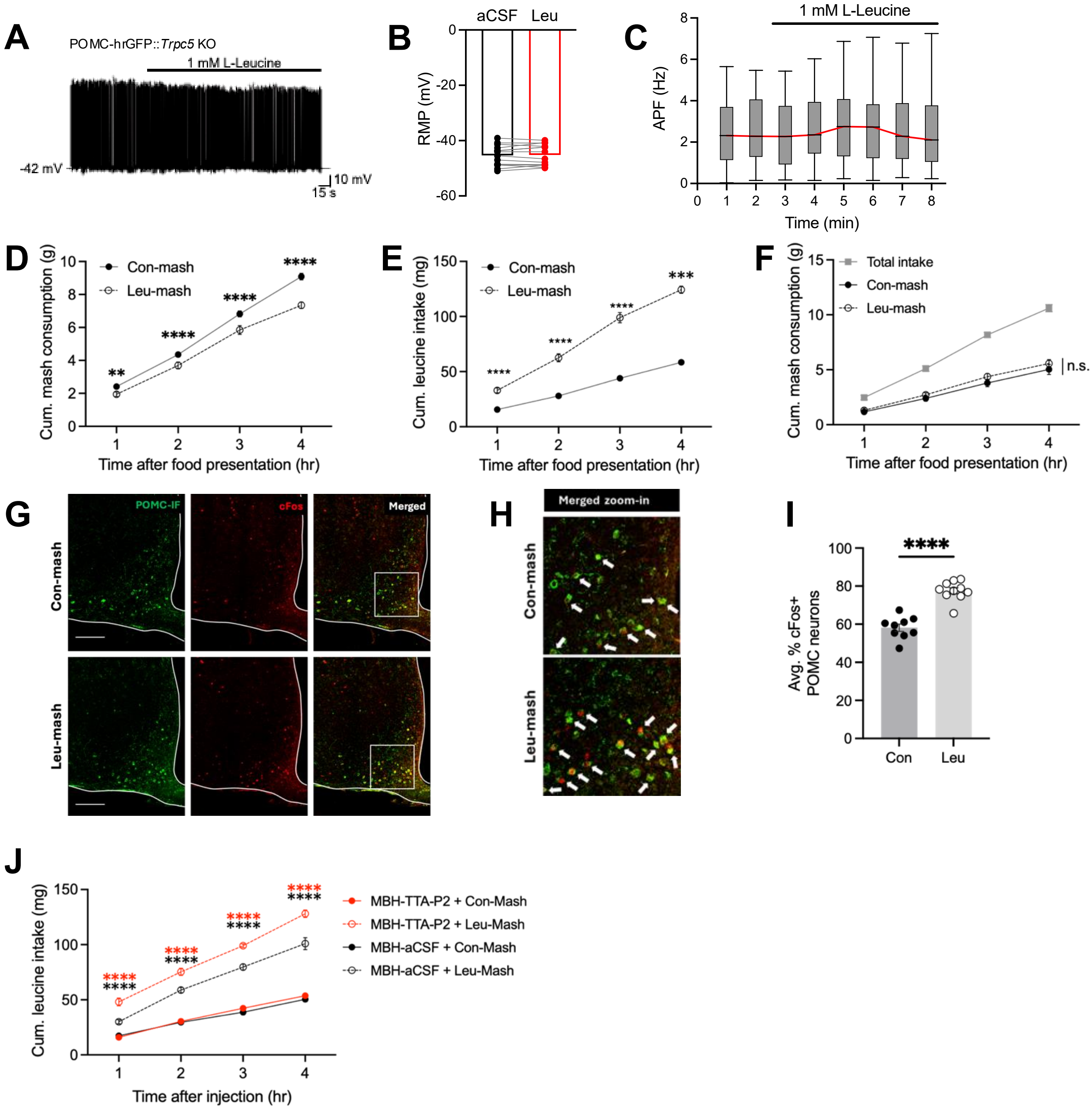

A
POMC-hrGFP::Trpc5 KO
1 mM L-Leucine
-42 mV
10 mV
15 s
B
aCSF
Leu
RMP (mV)
C
1 mM L-Leucine
APF (Hz)
Time (min)
D
Con-mash
Leu-mash
Cum. mash consumption (g)
Time after food presentation (hr)
E
Con-mash
Leu-mash
Cum. leucine intake (mg)
Time after food presentation (hr)
F
Total intake
Con-mash
Leu-mash
n.s.
Cum. mash consumption (g)
Time after food presentation (hr)
G
POMC-IF
cFos
Merged
Con-mash
Leu-mash
H
Merged zoom-in
Con-mash
Leu-mash
I
Avg. % cFos+ POMC neurons
Con
Leu
J
MBH-TTA-P2 + Con-Mash
MBH-TTA-P2 + Leu-Mash
MBH-aCSF + Con-Mash
MBH-aCSF + Leu-Mash
Cum. leucine intake (mg)
Time after injection (hr)

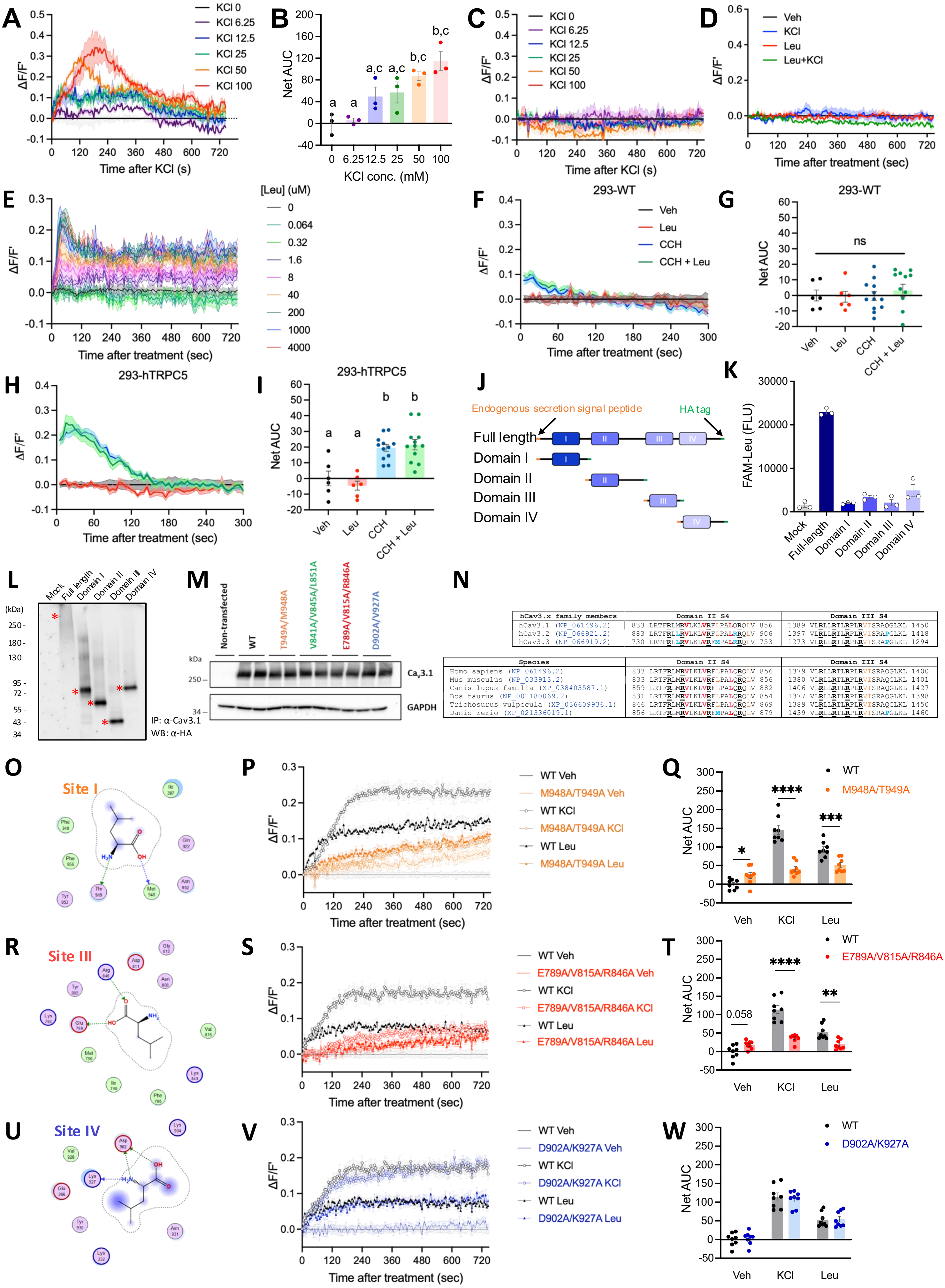
A
ΔF/F'
Time after KCl (s)
KCl 0
KCl 6.25
KCl 12.5
KCl 25
KCl 50
KCl 100
B
Net AUC
KCl conc. (mM)
a
a,c
b,c
C
Time after KCl (s)
D
Veh
KCl
Leu
Leu+KCl
Time after treatment (sec)
E
[Leu] (uM)
0
0.064
0.32
1.6
8
40
200
1000
4000
Time after treatment (sec)
F
293-WT
Veh
Leu
CCH
CCH + Leu
G
293-WT
ns
H
293-hTRPC5
I
293-hTRPC5
J
Endogenous secretion signal peptide
HA tag
Full length
Domain I
Domain II
Domain III
Domain IV
K
FAM-Leu (FLU)
Mock
L
(kDa)
IP: α-Cav3.1
WB: α-HA
M
Non-transfected
WT
T949A/M948A
V841A/V845A/L851A
E789A/V815A/R846A
D902A/V927A
kDa
$Ca_v3.1$
GAPDH
N
hCav3.x family members
Domain II S4
Domain III S4
Species
Homo sapiens
Mus musculus
Canis lupus familia
Bos taurus
Trichosurus vulpecula
Danio rerio
O
Site I
P
WT Veh
M948A/T949A Veh
WT KCl
M948A/T949A KCl
WT Leu
M948A/T949A Leu
Q
WT
M948A/T949A
Veh
KCl
Leu
R
Site III
S
WT Veh
E789A/V815A/R846A Veh
WT KCl
E789A/V815A/R846A KCl
WT Leu
E789A/V815A/R846A Leu
T
WT
E789A/V815A/R846A
0.058
U
Site IV
V
WT Veh
D902A/K927A Veh
WT KCl
D902A/K927A KCl
WT Leu
D902A/K927A Leu
W
WT
D902A/K927A

Figure S4: Additional data related to Figures 4

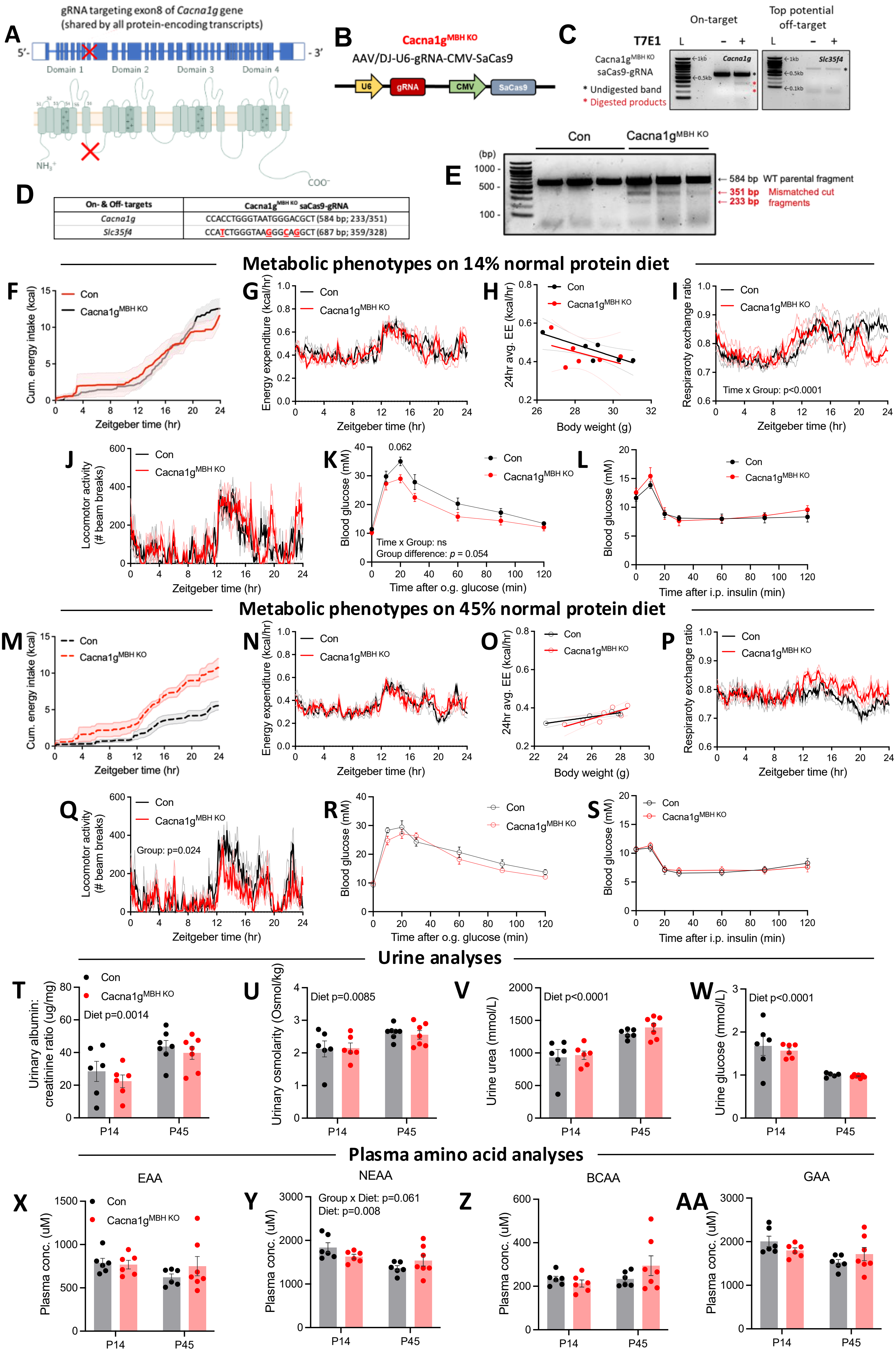

Figure S5: Hypothalamic *Canca1g* is downregulated by diet-induced obesity, related Figure 4

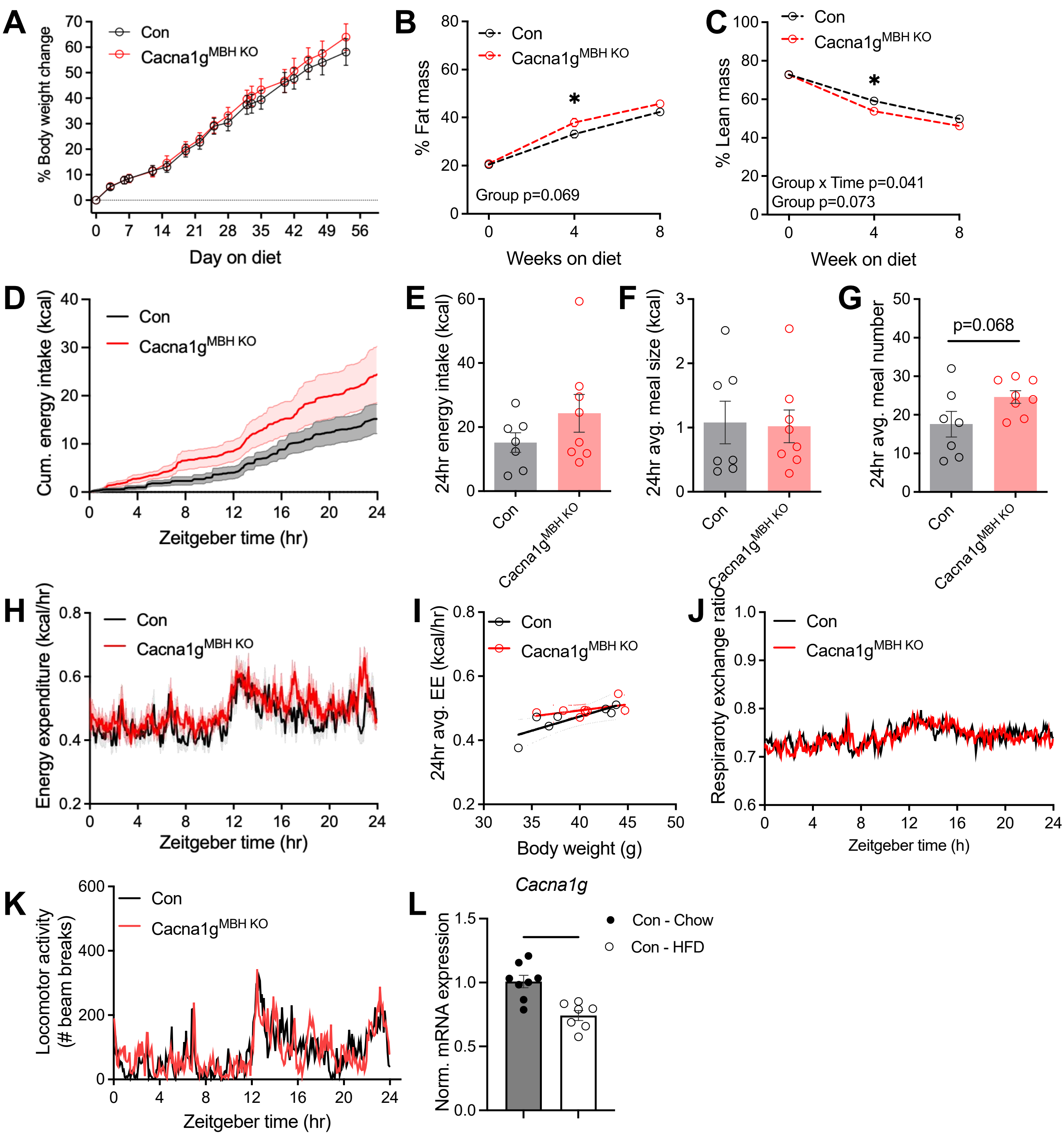

Figure S6: Additional data related to Figure 5

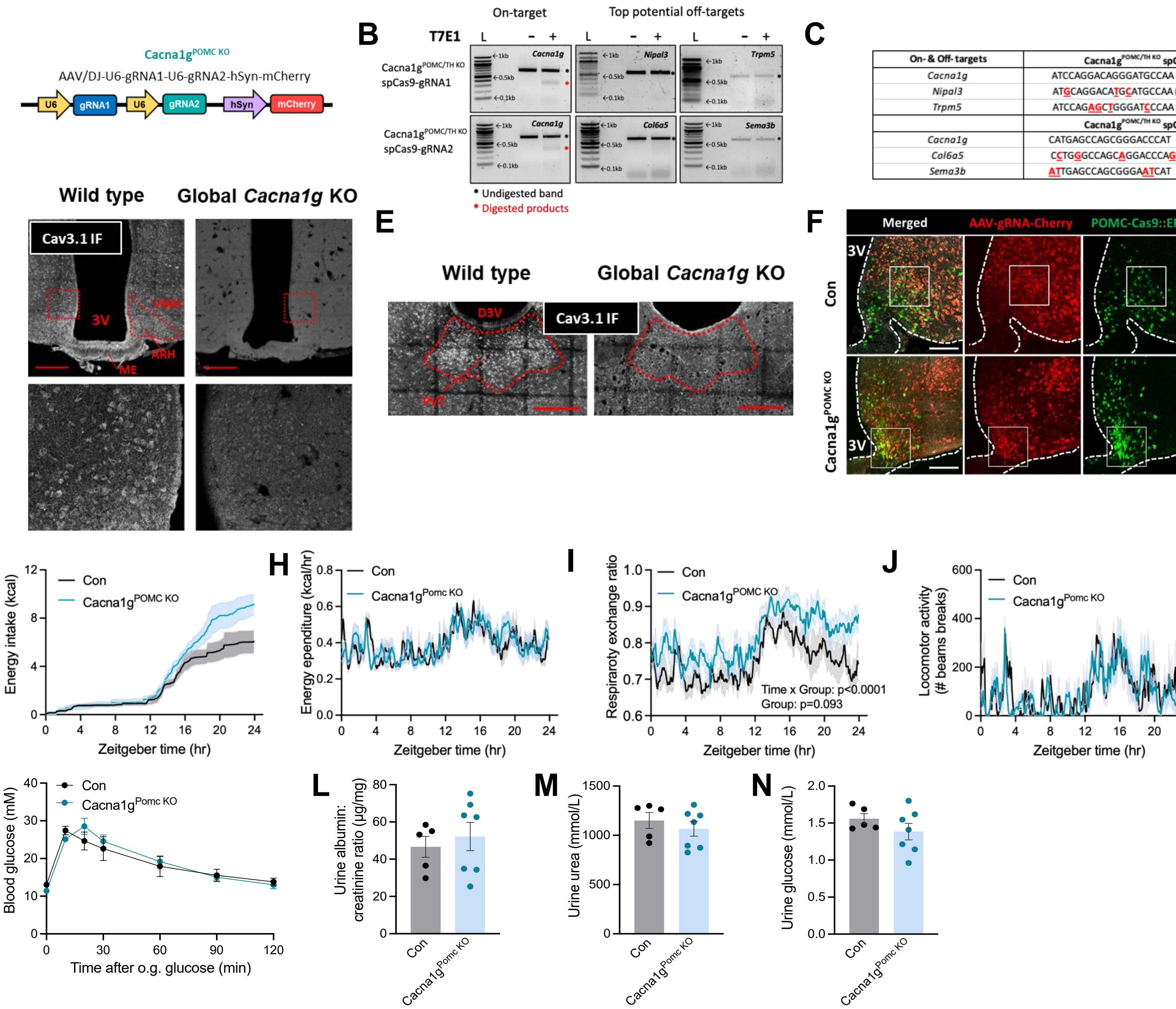

Figure S7: Additional data related to Figure 6

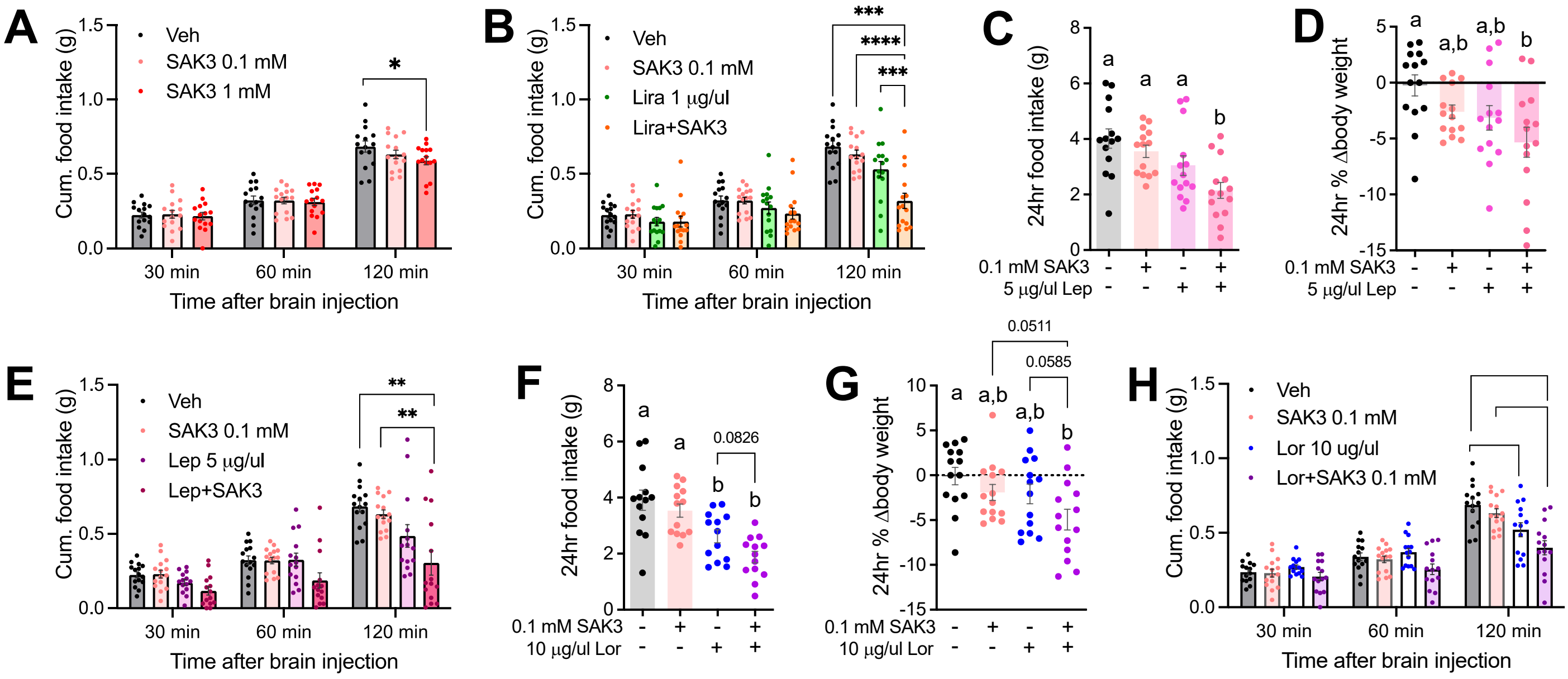

Additional figures in rebuttal

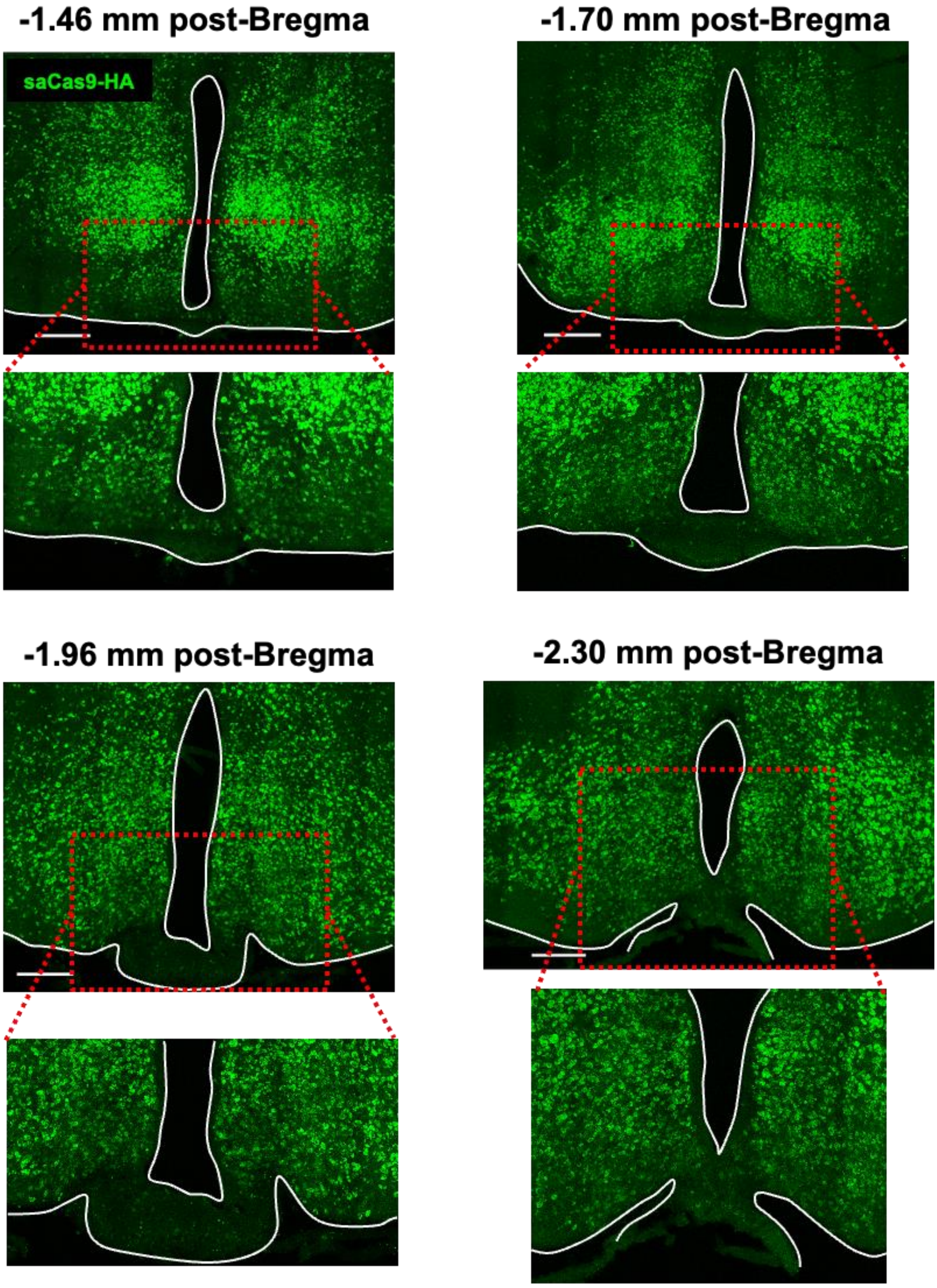

Scale bar: 500µm

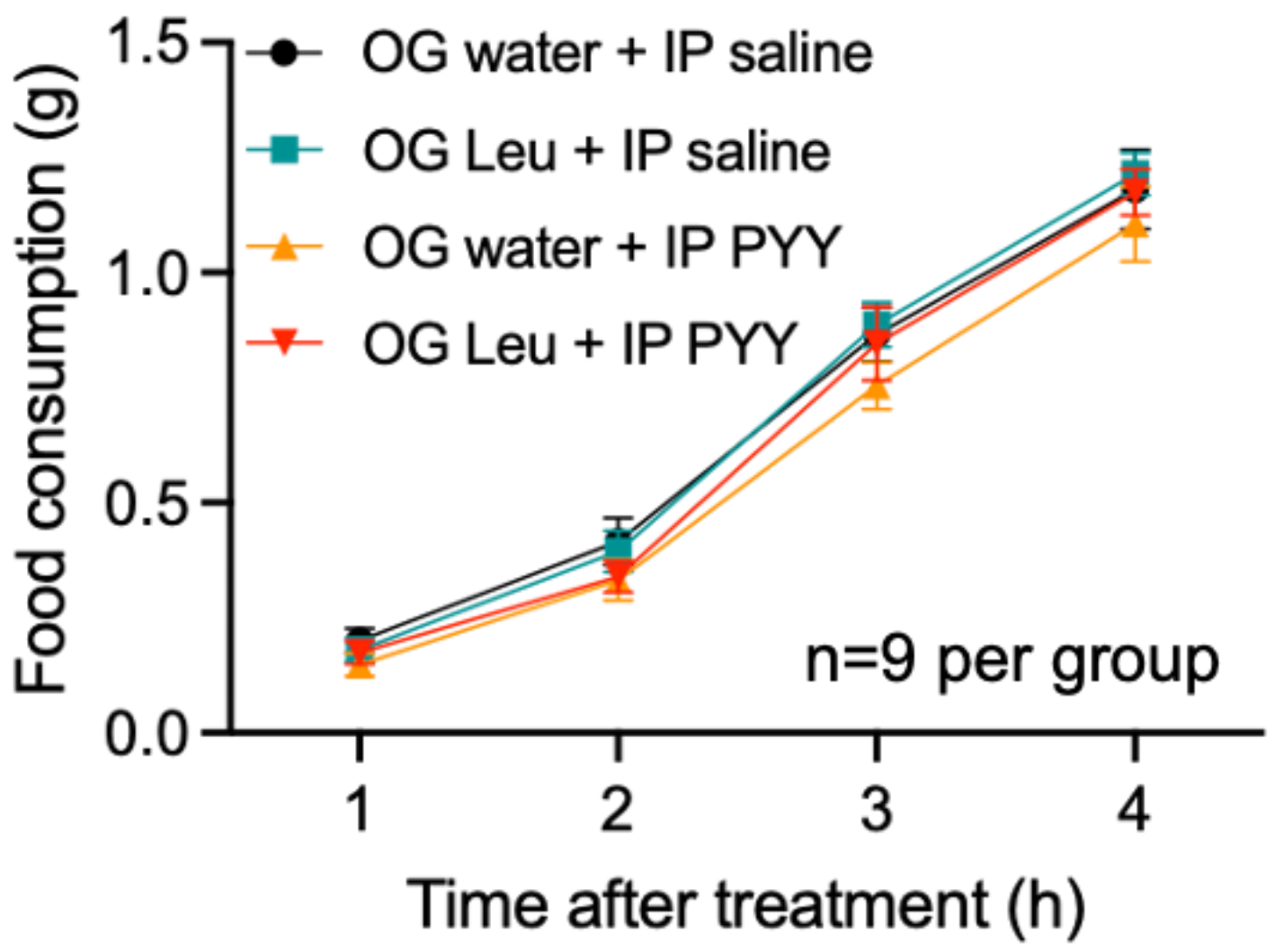

Food consumption (g)
1.5
1.0
0.5
0.0
OG water + IP saline
OG Leu + IP saline
OG water + IP PYY
OG Leu + IP PYY
n=9 per group
1
2
3
4
Time after treatment (h)

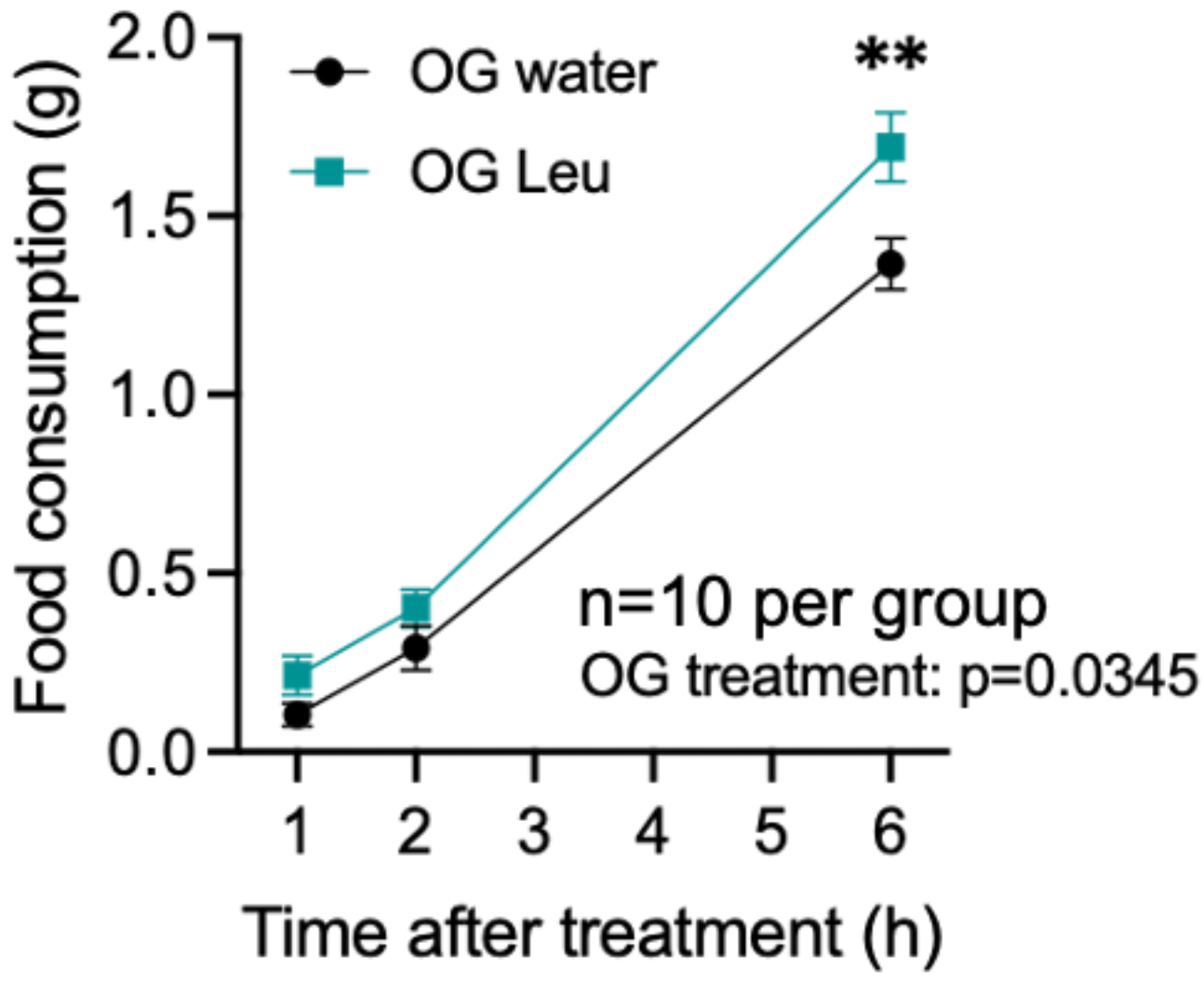

Food consumption (g)
2.0
1.5
1.0
0.5
0.0
OG water
OG Leu
**
n=10 per group
OG treatment: p=0.0345
1
2
3
4
5
6
Time after treatment (h)

Potential leucine binding Site 1: M948 & T949 in hCAV3.1

M 948/ T949

Homo sapiens	NP_061496.2	923	EDWNKVLYNGMASTSSWAALYFIALMTFGNYVLFNLLVAILVEGFQAEEISKREDASGQLSCIQLPVDS	QGGDANK	998
Mus musculus	NP_033913.2	923	EDWNKVLYNGMASTSSWAALYFIALMTFGNYVLFNLLVAILVEGFQAEEIGKREDTSGQLSCIQLPVNS	QGGDATK	998
Canis lupus familiaris	XP_038403587.1	949	EDWNKVLYNGMASTSSWAALYFIALMTFGNYVLFNLLVAILVEGFQAEEISKREDASGQLSCIQLPVDS	QGGDATK	1024
Bos taurus	NP_001180069.2	921	EDWNKVLYNGMASTSSWAALYFIALMTFGNYVLFNLLVAILVEGFQTEEISKREDASGQLSCIQLPVDS	QGGDATK	996
Trichosurus vulpecula	XP_036609936.1	936	EDWNKVLYNGMASTSSWAALYFIALMTFGNYVLFNLLVAILVEGFQTEEISKREDAGGQLSCIQLPVDS	LGGDATK	1011
Danio rerio	XP_021336019.1	946	EDWNKVLYNGMASTSPVAALYFIALMTFGNYVLFNLLVAILVEGFQTEEITKRDDLHGQLSCIQLPIDS[24]	LAGDASK	1045

Potential leucine binding site 2: E789, V815 and R846 in hCAV3.1

E 789 V 815

NP_061496.2	763	EYHEQPEELTNALEISNIVFTSLFALEMLLKLLVYGPFGYIKNPYNIFDGVIVVISVWEIVGQQGGGLSVLRTFRLMRVL	842
NP_033913.2	763	EYHEQPEELTNALEISNIVFTSLFALEMLLKLLVYGPFGYIKNPYNIFDGVIVVISVWEIVGQQGGGLSVLRTFRLMRVL	842
XP_038403587.1	789	EYHEQPEELTNALEISNIVFTSLFALEMLLKLLVYGPFGYIKNPYNIFDGVIVVISVWEIVGQQGGGLSVLRTFRLMRVL	868
NP_001180069.2	761	EYHEQPEELTNALEISNIVFTSLFALEMLLKLLVYGPFGYIKNPYNIFDGVIVVISVWEIVGQQGGGLSVLRTFRLMRVL	840
XP_036609936.1	776	EYHEQPEELTNALEISNIVFTSLFALEMLLKLLVYGPFGYIKNPYNIFDGVIVVISVWEIVGQQGGGLSVLRTFRLMRVL	855
XP_021336019.1	786	EYHEQPDELTNALEISNIVFTSLFVLEMLLKLLVYGPFGYIKNPYNIFDGIIVVISVWEIVGQQGGGLSVLRTFRLMRVL	865

NP_061496.2	843	KLVRFLPALQRQLVVLMKTMDNVATFCMLLMLFIFIFSILGMHLFGCKFASERDGDTLPDRKNFDSLLWAIVTVFQILTQ	922
NP_033913.2	843	KLVRFLPALQRQLVVLMKTMDNVATFCMLLMLFIFIFSILGMHLFGCKFASERDGDTLPDRKNFDSLLWAIVTVFQILTQ	922
XP_038403587.1	869	KLVRFLPALQRQLVVLMKTMDNVATFCMLLMLFIFIFSILGMHLFGCKFASERDGDTLPDRKNFDSLLWAIVTVFQILTQ	948
NP_001180069.2	841	KLVRFLPALQRQLVVLMKTMDNVATFCMLLMLFIFIFSILGMHLFGCKFASERDGDTLPDRKNFDSLLWAIVTVFQILTQ	920
XP_036609936.1	856	KLVRFLPALQRQLVVLMKTMDNVATFCMLLMLFIFIFSILGMHLFGCKFASERDGDTLPDRKNFDSLLWAIVTVFQILTQ	935
XP_021336019.1	866	KLVRFMPALQRQLVVLMKTMDNVATFCMLLMLFIFIFSILGMHLFGCKFGSERDGDTLPDRKNFDSLLWAIVTVFQILTQ	945

R 846

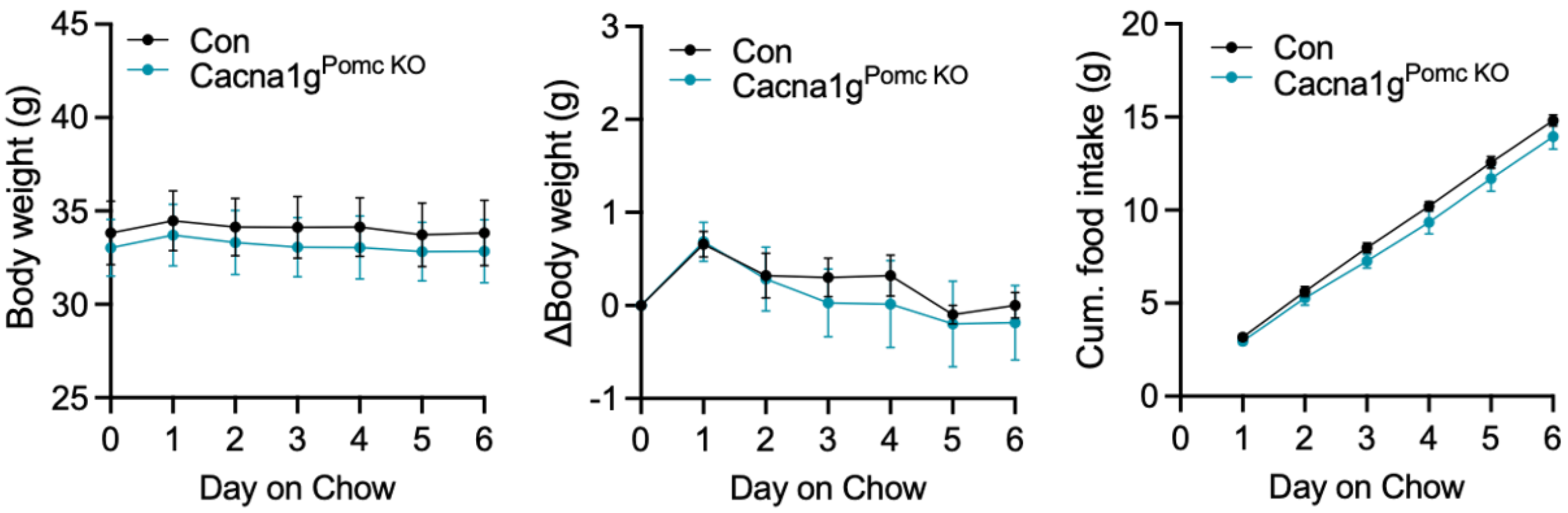
45
40
35
30
25
Body weight (g)
Con
Cacna1g^Pomc KO
0
1
2
3
4
5
6
Day on Chow
3
2
1
0
-1
ΔBody weight (g)
Con
Cacna1g^Pomc KO
0
1
2
3
4
5
6
Day on Chow
20
15
10
5
0
Cum. food intake (g)
Con
Cacna1g^Pomc KO
0
1
2
3
4
5
6
Day on Chow

Supplemental figure S4

Click here to access/download;Supplemental Text and Figures;Figure S4.tiff

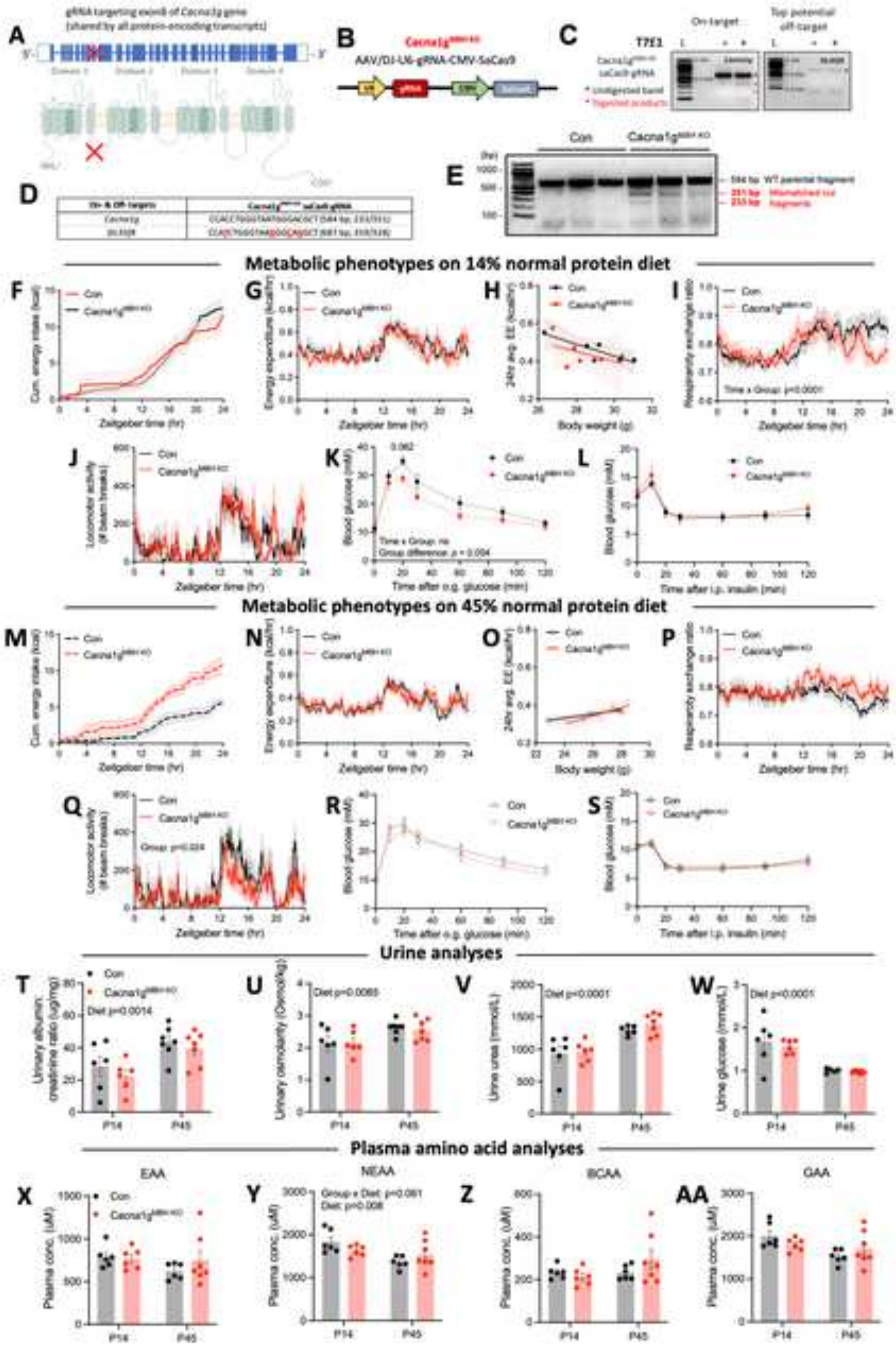

Supplemental figure S5

Click here to access/download;Supplemental Text and Figures;Figure S5.tiff

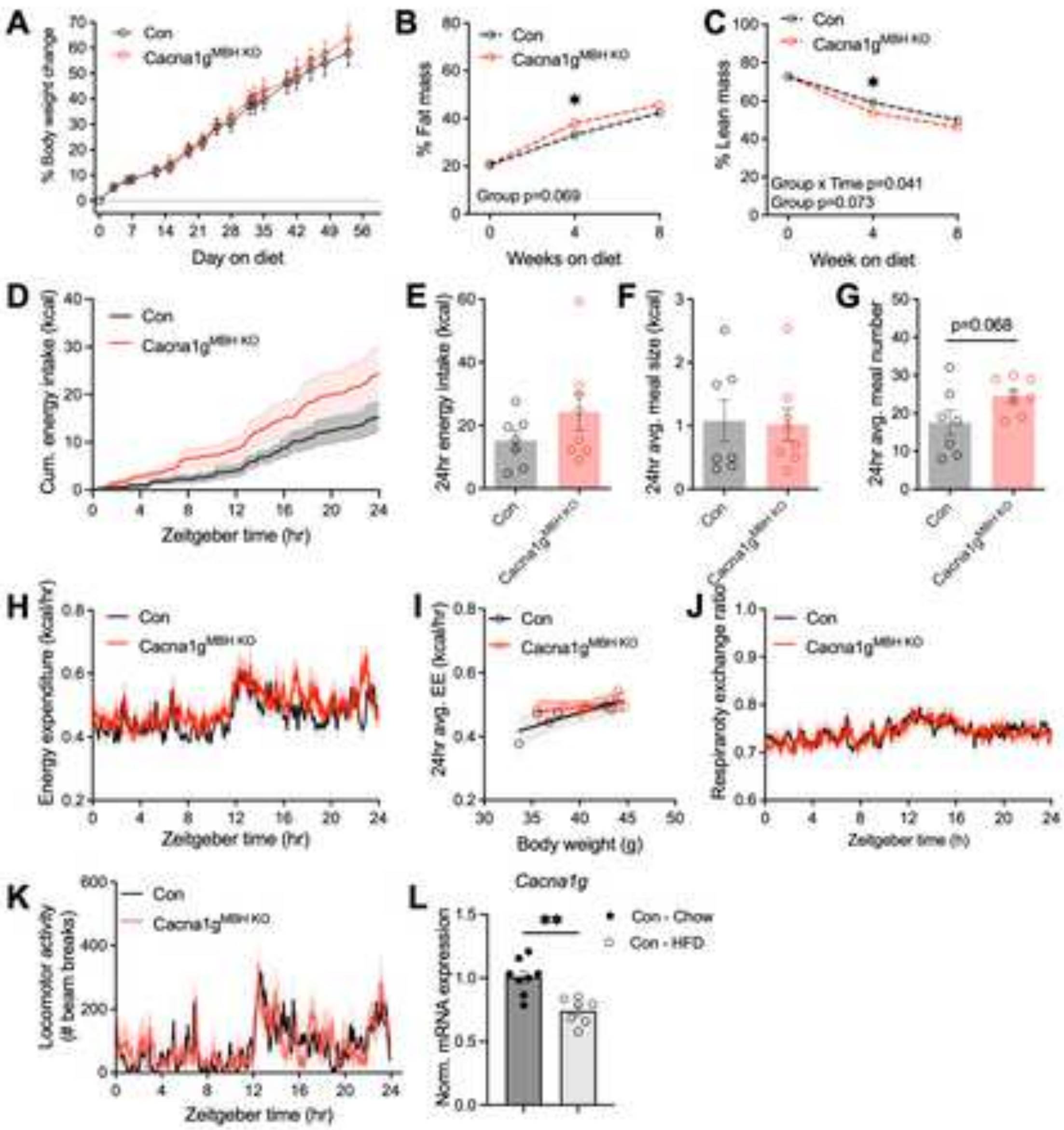

Supplemental figure S6

Click here to access/download;Supplemental Text and Figures;Figure S6.tiff

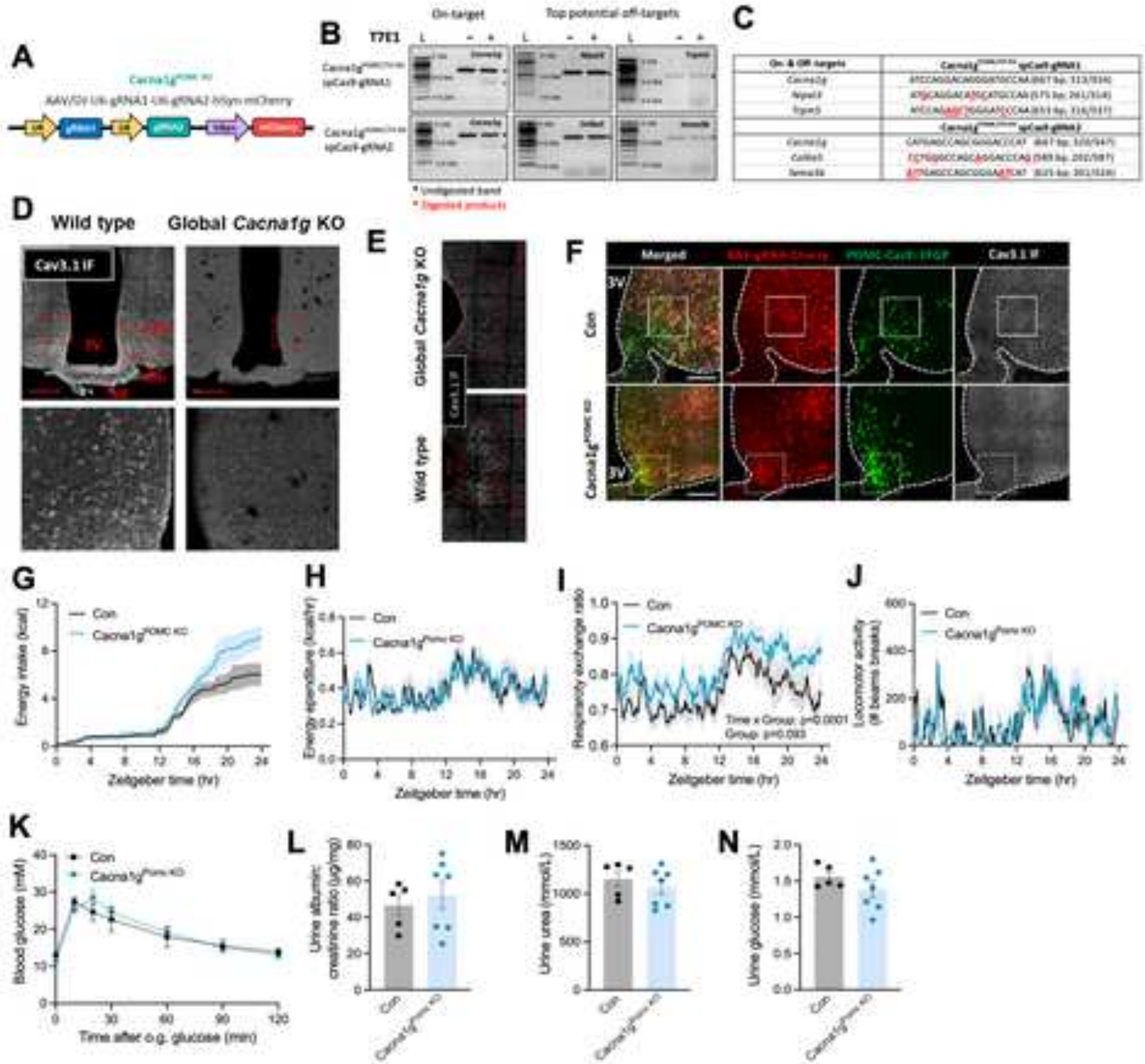

Supplemental figure S7

Click here to access/download;Supplemental Text and Figures;Figure S7.tiff

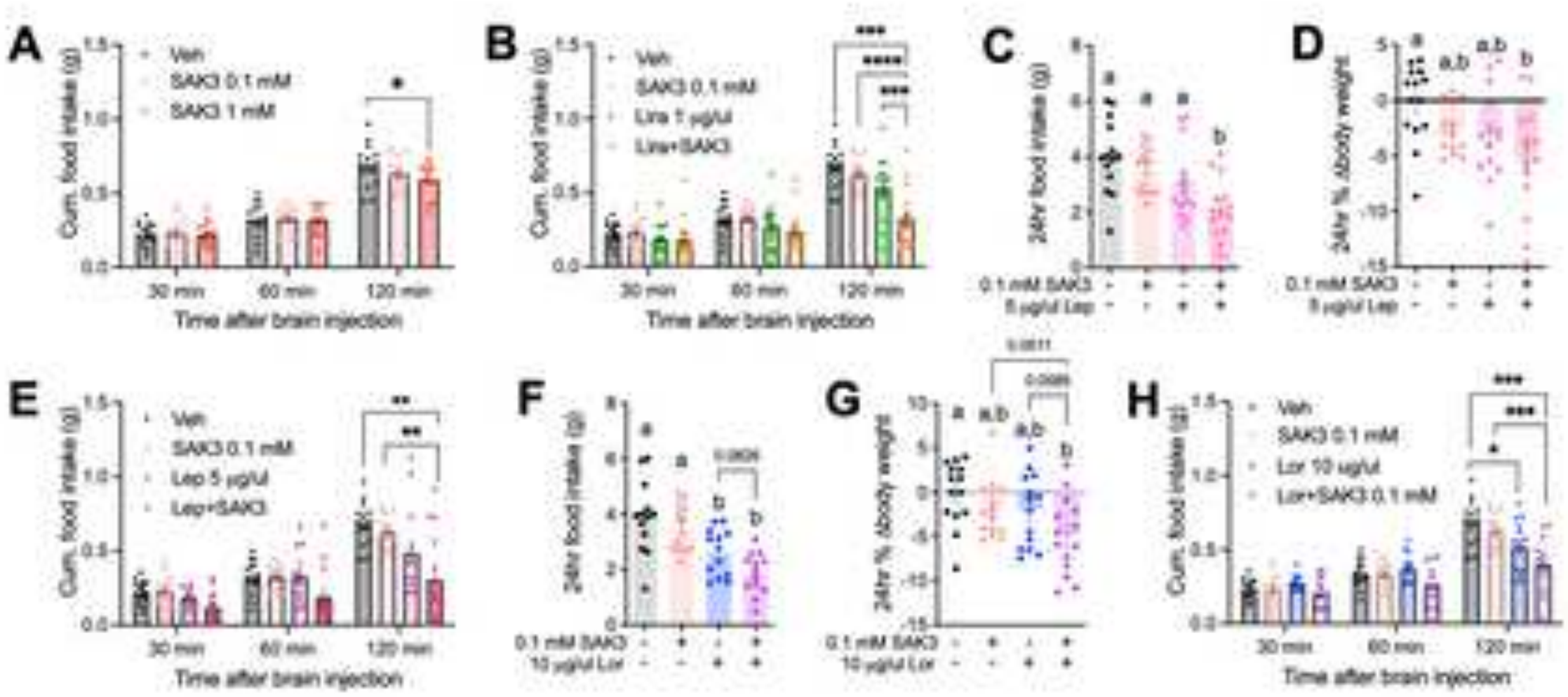

Click here to access/download
Supplemental Videos and Spreadsheets
Table S1. MBH Leu PhosphoTRAP RNAseq data.xlsx

Click here to access/download
Supplemental Videos and Spreadsheets
Table S2. MBH Leu PhosphoTRAP top 20 genes.docx

Click here to access/download

Supplemental Videos and Spreadsheets

Table S3. MBH Leu PhosphoTRAP Plasma Membrane Genes.docx

Table S4

Click here to access/download
Supplemental Videos and Spreadsheets
Table S4. MBH Leu PhosphoTRAP Secreted Protein.docx

Table S5

Click here to access/download

Supplemental Videos and Spreadsheets

Table S5. MBH Leu PhosphoTRAP Ingenuity Pathways Analysis.xlsx

Click here to access/download
Supplemental Videos and Spreadsheets
Table S6. P14, P45 diet composition.docx

Table S7

Click here to access/download

Supplemental Videos and Spreadsheets

Table S7. Cacna1g MBH KO plasma amino acid profile.docx

Click here to access/download
ZIP File
Data S1 - Source data.zip

CellPress

CELL PRESS DECLARATION OF INTERESTS POLICY

Transparency is essential for a reader's trust in the scientific process and for the credibility of published articles. At Cell Press, we feel that disclosure of competing interests is a critical aspect of transparency. Therefore, we require a "declaration of interests" section in which all authors disclose any financial or other interests related to the submitted work that (1) could affect or have the perception of affecting the author's objectivity or (2) could influence or have the perception of influencing the content of the article.

What types of articles does this apply to?
We require that you disclose competing interests for all submitted content by completing and submitting the form below. We also require that you include a "declaration of interests" section in the text of all articles even if there are no interests to declare.

What should I disclose?
We require that you and all authors disclose any personal financial interests (e.g., stocks or shares in companies with interests related to the submitted work or consulting fees from companies that could have interests related to the work), professional affiliations, advisory positions, board memberships (including membership on a journal's advisory board when publishing in that journal), or patent applications and/or registrations that are related to the subject matter of the contribution. As a guideline, you need to declare an interest for (1) any affiliation associated with a payment or financial benefit exceeding $10,000 p.a. or 5% ownership of a company or (2) research funding by a company with related interests. You do not need to disclose diversified mutual funds, 401ks, or investment trusts.

Authors should also disclose relevant financial interests of immediate family members. Cell Press uses the Public Health Service definition of "immediate family member," which includes spouse and dependent children.

Where do I declare competing interests?
Competing interests should be disclosed on this form as well as in a "declaration of interests" section in the manuscript. This section should include financial or other competing interests as well as affiliations that are not included in the author list. Examples of "declaration of interests" language include:

> "AUTHOR is an employee and shareholder of COMPANY."
> "AUTHOR is a founder of COMPANY and a member of its scientific advisory board."

NOTE: Primary affiliations should be included with the author list and do not need to be included in the "declaration of interests" section. Funding sources should be included in the "acknowledgments" section and also do not need to be included in the "declaration of interests" section. (A small number of front-matter article types do not include an "acknowledgments" section. For these articles, reporting of funding sources is not required.)

What if there are no competing interests to declare?
If you have no competing interests to declare, please note that in the "declaration of interests" section with the following wording:

> "The authors declare no competing interests."

April 12, 2024

CellPress

CELL PRESS DECLARATION OF INTERESTS FORM

If submitting materials via Editorial Manager, please complete this form and upload with your initial submission. Otherwise, please email as an attachment to the editor handling your manuscript.

Please complete each section of the form and insert any necessary "declaration of interests" statement in the text box at the end of the form. A matching statement should be included in a "declaration of interests" section in the manuscript.

Institutional affiliations

We require that you list the current institutional affiliations of all authors, including academic, corporate, and industrial, on the title page of the manuscript. ***Please select one of the following:***

☒ All affiliations are listed on the title page of the manuscript.

☐ I or other authors have additional affiliations that we have noted in the "declaration of interests" section of the manuscript and on this form below.

Funding sources

We require that you disclose all funding sources for the research described in this work. ***Please confirm the following:***

☒ All funding sources for this study are listed in the "acknowledgments" section of the manuscript.*

*A small number of front-matter article types do not include an "acknowledgments" section. For these, reporting funding sources is not required.

Competing financial interests

We require that authors disclose any financial interests and any such interests of immediate family members, including financial holdings, professional affiliations, advisory positions, board memberships, receipt of consulting fees, etc., that:

(1) could affect or have the perception of affecting the author's objectivity, *or*
(2) could influence or have the perception of influencing the content of the article.

Please select one of the following:

☒ We, the authors and our immediate family members, have no financial interests to declare.

☒ We, the authors, have noted any financial interests in the "declaration of interests" section of the manuscript and on this form below, and we have noted interests of our immediate family members.

Advisory/management and consulting positions
We require that authors disclose any position, be it a member of a board or advisory committee or a paid consultant, that they have been involved with that is related to this study. We also require that members of our journal advisory boards disclose their position when publishing in that journal. ***Please select one of the following:***

- ☒ We, the authors and our immediate family members, have no positions to declare and are not members of the journal's advisory board.
- ☐ The authors and/or their immediate family members have management/advisory or consulting relationships noted in the "declaration of interests" section of the manuscript and on this form below.

Patents
We require that you disclose any patent applications and/or registrations related to this work by any of the authors or their institutions. ***Please select one of the following:***

- ☒ We, the authors and our immediate family members, have no related patent applications or registrations to declare.
- ☐ We, the authors, have a patent application and/or registration related to this work, which is noted in the "declaration of interests" section of the manuscript and on this form below, and we have noted the patents of immediate family members.

Please insert any "declaration of interests" statements in this space. This exact text should also be included in the "declaration of interests" section of the manuscript. If no authors have a competing interest, please insert the text, "The authors declare no competing interests."

☒ **On behalf of all authors, I declare that I have disclosed all competing interests related to this work. If any exist, they have been included in the "declaration of interests" section of the manuscript.**
